# Factors influencing bird-building collisions in the downtown area of a major North American city

**DOI:** 10.1371/journal.pone.0224164

**Published:** 2019-11-06

**Authors:** Scott R. Loss, Sirena Lao, Joanna W. Eckles, Abigail W. Anderson, Robert B. Blair, Reed J. Turner

**Affiliations:** 1 Department of Natural Resource Ecology and Management, Oklahoma State University, Stillwater, Oklahoma, United States of America; 2 Audubon Minnesota, St. Paul, Minnesota, United States of America; 3 Department of Fisheries, Wildlife, and Conservation Biology, University of Minnesota, St. Paul, Minnesota, United States of America; Auburn University, UNITED STATES

## Abstract

Bird-building collisions are the largest source of avian collision mortality in North America. Despite a growing literature on bird-building collisions, little research has been conducted in downtown areas of major cities, and no studies have included stadiums, which can be extremely large, often have extensive glass surfaces and lighting, and therefore may cause many bird collisions. Further, few studies have assessed the role of nighttime lighting in increasing collisions, despite the often-cited importance of this factor, or considered collision correlates for different seasons and bird species. We conducted bird collision monitoring over four migration seasons at 21 buildings, including a large multi-use stadium, in downtown Minneapolis, Minnesota, USA. We used a rigorous survey methodology to quantify among-building variation in collisions and assess how building features (e.g., glass area, lighting, vegetation) influence total collision fatalities, fatalities for separate seasons and species, and numbers of species colliding. Four buildings, including the stadium, caused a high proportion of all collisions and drove positive effects of glass area and amount of surrounding vegetation on most collision variables. Excluding these buildings from analyses resulted in slightly different collision predictors, suggesting that factors leading some buildings to cause high numbers of collisions are not the exact same factors causing variation among more typical buildings. We also found variation in collision correlates between spring and fall migration and among bird species, that factors influencing collision fatalities also influence numbers of species colliding, and that the proportion, and potentially area, of glass lighted at night are associated with collisions. Thus, reducing bird collisions at large buildings, including stadiums, should be achievable by reducing glass area (or treating existing glass), reducing light emission at night, and prioritizing mitigation efforts for glass surfaces near vegetated areas and/or avoiding use of vegetation near glass.

## Introduction

Up to 1.5 billion birds are killed annually in North America by colliding with vehicles and human-made structures, including buildings, communication towers, and energy infrastructure [1–3]. Bird-building collisions, particularly collisions with windows and other reflective surfaces, are by far the largest source of avian collision mortality, annually causing 365 to 988 million bird fatalities in the United States [4] and 16 to 42 million fatalities in Canada [5]. Bird-building collisions are most frequent in urban areas containing many residential and commercial structures; however, the species most frequently killed, as well as those appearing most vulnerable to population-level impacts of building collision fatalities, are migratory birds that collide during spring and fall while in transit between breeding and nonbreeding grounds (e.g., hummingbirds, warblers, thrushes, and native sparrows) [4, 6].

Rates of bird-building collisions are influenced by many factors that interact across multiple spatial and temporal scales. At small scales, collisions are influenced by features of buildings (e.g., size, height, and window/glass area) [7–8] and their immediate surroundings (e.g., nearby vegetation and greenspace) [9–13]. Such small-scale effects also appear to be mediated by regional patterns of urbanization and greenspace [14]. Collisions also vary through time in relation to bird migratory movements and changes in weather, bird behavior, and human-related factors that influence bird migration, behavior, and habitat use (e.g., use of ornamental vegetation, bird feeders, and artificial light at night, which confuses and attracts nocturnally migrating birds, elevating collision risk) [15–18]. Collisions are also influenced by the abundance of birds near buildings [19–21] and by traits of birds themselves, including visual perceptual ability [22–23] and life history (e.g., residency status, migratory strategy) [24–26].

Despite a growing literature on bird-building collisions, many important information gaps remain. First, few replicated, standardized studies have been conducted in downtown areas of major cities, where per building collision rates peak [4] likely as a result of the large size of buildings and intense nighttime lighting [27–28]. Second, few studies have investigated collisions at large buildings other than skyscrapers (but see [10, 20]), and none have focused on a stadium. Research at stadiums would be beneficial because, in addition to their large size, many of the hundreds of existing and planned stadiums in North America have extensive glassy surfaces and are brightly illuminated by external and internal lighting during spring and/or fall migration periods. Many stadiums thus appear capable of causing high bird collision rates. Third, while nighttime lighting is frequently cited as a factor contributing to building collisions, few formal assessments have been conducted (but see [18, 29]). Fourth, most collision studies, including the most rigorous studies in downtown areas [7, 21, 30], have not accounted for scavenger and human removal of bird carcasses between collision surveys or for imperfect detection of carcasses that are present. Failing to account for these factors causes underestimation of collisions and can mislead comparisons among buildings [31–33]. Further, rates of human removal of bird carcasses (e.g., by cleaning crews) are often much greater in downtown areas than on university campuses or in residential neighborhoods where past removal studies were conducted. Fifth, few studies of bird-building collisions have gone beyond assessing factors influencing total collisions to also investigate collision correlates for different seasons and bird species. Such information would provide valuable insight into developing effective collision reduction approaches that target certain seasons (e.g., fall migration, when collisions peak in most regions) and species (e.g., endangered/declining species with collision correlates that may differ from common species). Finally, although species composition of birds killed at windows appears influenced by features of the surrounding landscape [25], no studies have formally investigated how building and landscape-related factors influence the number of species that collide at a building.

To address these research gaps, we conducted a bird collision monitoring study that covered four migration seasons and included 21 buildings, including a large multi-use stadium, in downtown Minneapolis, Minnesota, USA. We used a rigorous methodology that included daily standardized collision surveys at all buildings and experimental trials to estimate and account for removal and imperfect surveyor detection of bird carcasses. Our research questions were: (1) How do numbers of bird collisions vary among the monitored buildings? (2) What building features (e.g., height, glass area, nighttime lighting, and surrounding vegetation and greenspace) influence collision fatalities, including total fatalities, fatalities in spring and fall, and fatalities for the most frequently colliding species? and (3) What building features influence numbers of species that collide, including overall and in spring and fall?

## Materials and methods

### Study area and design

We conducted bird collision monitoring at 21 buildings in downtown Minneapolis, Minnesota (44.9772 ˚ N, 93.2637˚ W), which is immediately west of the Mississippi River—the largest river system in North America and an important bird migration corridor—and is part of the Minneapolis-St. Paul (Twin Cities) metropolitan region (population = ~3.1 million people). The Twin Cities are located near the intersection of the North Central Hardwoods and Western Corn Belt Plains Level III Ecoregions of the United States [34]; non-urban land cover types surrounding and within the Twin Cities include forests and woodlands dominated by deciduous species, numerous lakes and wetlands, extensive croplands, and limited grassland cover.

Due to interests of the funding organizations, U.S. Bank Stadium formed the initial basis for the research and was therefore non-randomly selected to be studied. This indoor stadium was completed in summer 2016. Concerns about the risk of bird collisions at the stadium were raised in 2012 [35] and repeated in 2013 when the stadium design was revealed to have several elements making it likely to cause bird collisions [36]. These elements include approximately 18,000 m^2^ (i.e., 1.8 ha, or ~37% of the stadium’s vertical surfaces) of highly reflective glass surfaces throughout the building’s exterior—including approximately 6,000 m^2^ of uninterrupted glass on one portion of the stadium’s northwest façade, which faces an open park space with trees and manicured lawns—and the use of LED lighting at night inside, outside, and directed onto the stadium, and in ground-based lighting features on the stadium grounds.

**Fig 1:**
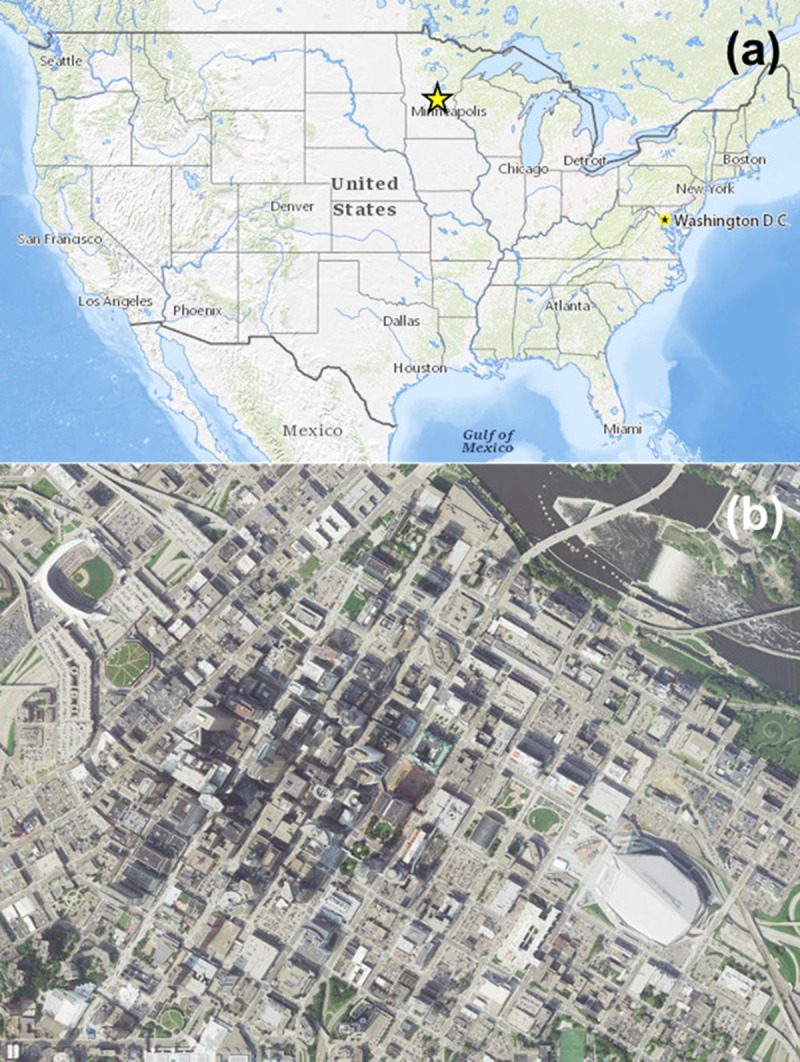
Study area. (a) General location of study area in the United States and (b) location of study area containing 21 buildings, including U.S. Bank Stadium (large, gray, irregularly shaped building in lower right of image), monitored for bird collisions in downtown Minneapolis, Minnesota, USA, 2017–2018; image sources: USGS National Map Viewer base map (a) and NAIP Plus aerial imagery (b).

In addition to the stadium, 20 buildings were selected for monitoring (Fig 1). Sixteen of these were selected from a set of 64 downtown Minneapolis buildings that were monitored for collisions from 2007 to 2016 for Project BirdSafe, a research, outreach, and education program with the goals of increasing awareness of the bird collision issue and working with building managers and policy makers to develop and implement collision reduction guidelines [37]. These 64 buildings were grouped into quintiles (groupings of 0–20%, 20–40%, etc.,) using total collisions observed from 2007 to 2015; we did not use 2016 Project BirdSafe data because fieldwork was ongoing when we began designing the current study in fall 2016. From each quintile, we randomly selected three buildings (15 total) with the constraints that: (1) building perimeters at ground level were 50–100% accessible (this range of percentages balanced the need for building access with the need to include a large enough sample of buildings for each quintile); and (2) buildings captured a broad spatial representation of the downtown area, especially with regard to distance to the Mississippi River, a factor we expected to influence collisions due to the importance of this corridor for migratory birds [38]. Shortly after study initiation, we selected one additional building from the 80-100^th^ percentile because part of one originally-selected building from this quintile was inaccessible in spring 2017. Because the stadium was spatially separate from these other buildings, we also selected four previously unmonitored buildings within 0.7 km of the stadium and under the same access constraint as above. The resultant 20 buildings represented a variety of structures typical to downtown areas; they ranged from 2 to 57 stories and included hotels, apartments, and office buildings (building characteristics in Table 1).

**Table 1:** Characteristics of monitored buildings.

								Prop. vegetation^i^	
Building ID^a^	Quintile^b^	Height (m)^c^	Glass area (m^2^)^d^	Area light (m^2^)^e^	Prop. light^f^	Footprint (m^2^)^g^	Distance to river (m)^h^	50 m buffer	100 m buffer
1 (Stadium)	NA	83	11,319	7,722	0.68	51,863	612	0.16	0.10
2	1	26	980	494	0.50	5,956	955	0.01	0.02
3	5	139	4,255	996	0.23	3,233	998	0.22	0.10
4	5	241	16,913	2,454	0.15	2,415	1,096	0.00	0.01
5	3	19	1,825	232	0.13	2,576	494	0.02	0.02
6	4	127	1,434	624	0.44	4,727	660	0.00	0.01
7	2	95	682	128	0.19	1,029	831	0.00	0.00
8	2	46	782	375	0.48	1,583	999	0.00	0.01
9	5	64	3,476	1,112	0.32	3,835	857	0.03	0.03
10	4	73	452	234	0.52	1,522	761	0.01	0.01
11	4	34	2,165	367	0.17	1,504	553	0.06	0.03
12	3	30	1,947	895	0.46	2,725	538	0.02	0.03
13	1	61	1,651	1,317	0.80	5,762	1,368	0.00	0.00
14	1	26	452	172	0.38	4,294	1,290	0.01	0.01
15	3	171	8,245	1,772	0.21	3,724	741	0.00	0.00
16	2	12	296	23	0.08	1,505	1,407	0.04	0.03
17	5	123	6,537	4,277	0.65	4,615	811	0.19	0.12
18	NA	29	773	233	0.30	1,636	338	0.00	0.01
19	NA	92	3,698	261	0.07	5,461	451	0.03	0.12
20	NA	15	4,476	1,048	0.23	5,779	385	0.04	0.05
21	NA	19	933	377	0.40	2,799	398	0.00	0.00

Characteristics of 21 buildings, including U.S. Bank Stadium, monitored for bird collisions in downtown Minneapolis, Minnesota, USA, 2017–2018.

^a^Unique numeric code for each building used for purposes of current study.

^b^For buildings previously monitored in Project BirdSafe, the quintile into which they were placed for stratified random selection approach in the current study (see text for details); quintiles are based on total collisions observed across 64 buildings originally monitored in that earlier study (1 = 0–20 percentile of observed collisions; 2 = 20–40%; 3 = 40–60%; 4 = 60–80%; 5 = 80–100%; NA indicates buildings with no past history of collision monitoring).

^c^Estimated height of the main roof of the building.

^d^Total estimated area of glass (including windows and other glass surfaces) across all building facades, excluding glass recessed from the main façade for which collision casualties were likely to land on elevated surfaces not covered by surveys.

^e^Area of all windows emitting artificial light during nighttime periods.

^f^Proportion of all glass surfaces emitting artificial light during nighttime periods (calculated by dividing Area light by Glass area).

^g^Horizontal ground area covered by the building (based on building’s outer edge).

^h^Distance from building centroid to nearest edge of the Mississippi River corridor

^i^Proportion of land covered by vegetation within 50 and 100m of building (includes grass/shrub and deciduous/coniferous trees; excludes bare soil, roads and other paved surfaces, and other buildings)

### Collision surveys

We conducted daily collision monitoring at all 21 buildings during spring migration (15 Mar-31 May), early summer (1–30 Jun), and fall migration (15 Aug-31 Oct) of 2017 and 2018. We did not conduct monitoring in July or from November to early-March because relatively few collisions occur during these periods, both in downtown Minneapolis and elsewhere in central North America [3, 37]. There were some days within the above date ranges for which we were unable to survey all or a portion of some buildings due to safety considerations (e.g., construction or maintenance activities) or security measures associated with major events. However, the statistical estimator we used to adjust raw fatality counts for carcass removal and detection rates (see following sub-sections) accounted for this issue by allowing specification of varying time intervals between carcass searches.

We used a standardized survey protocol adapted from [39]. One day prior to each spring and fall season, “clean sweep” surveys were conducted in which we removed all bird carcasses and remains to avoid counting birds from non-surveyed periods. In spring 2017, buildings were split into two fixed routes, and the order in which they were surveyed was shifted by one building each day to account for time-of-day effects such as different patterns of human removal of bird carcasses at different buildings. In June 2017, the two routes were merged for the remainder of the study, and we used a random number generator to select the start building each day—with the exception of several days in fall 2017 when maintenance activities at the stadium required us to start there in order to avoid missing a survey. Throughout the study, we alternated the direction that building perimeters were monitored (clockwise on even dates; counter-clockwise on odd dates) to account for directional effects that could influence carcass detection, such as shading or physical obstructions. Surveys began at approximately sunrise and took 1.5 to 4 hours to complete depending on numbers of birds encountered. On a subset of days, we also conducted midday surveys (start time: 1000–1500 h) and evening surveys (start time: 1600–1800 h) at all buildings.

On all surveys, trained technicians or the authors searched for birds within ~5 m of all publicly accessible portions of building exteriors. For all carcasses or bird parts encountered, the location was marked on a building map and carcasses/remains were placed in a plastic bag and stored in a freezer until species identification was confirmed by the authors. We recorded bird carcasses with signs of dismemberment because, even though some of these could have resulted from predation events, we believed some likely represented collision victims that were scavenged by animals. We also recorded birds found below skyways (i.e., elevated glass walkways connecting to buildings) if it was uncertain whether the bird had collided with the skyway or the building itself. As described under “Bias-adjusted fatality estimates,” we generated separate collision counts that included and excluded these potential predation events and skyway collisions. When we found an injured bird, we attempted to catch it. Captured birds were placed in an uncoated paper bag, and those that recovered sufficiently were released later the same day in parks outside of downtown Minneapolis. Birds that did not recover sufficiently to be released were submitted to a wildlife rehabilitation center.

For the stadium, which experienced a large volume of foot traffic by the public, stadium staff, and contractors, we implemented an additional protocol for carcasses encountered by staff and contractors. Specifically, we asked the coordinator of stadium operations to periodically remind staff and contractors about the collision study and direct that any dead birds encountered be left in place when possible. In cases where it was deemed necessary to remove a bird, the carcass was to be submitted to central operations staff and stored in a freezer until collected by the authors. Given the difficulty of communicating this protocol to the hundreds of full-time, temporary, and touring staff that worked at the stadium over the two years of the study, this approach undoubtedly missed some human-removed bird carcasses. However, the design of our carcass removal experiment (see following sub-section) allowed us to account for both scavenger and human removal of carcasses at all buildings, including the stadium.

Because all fieldwork was conducted in publicly accessible areas of building exteriors, no specific access permissions were required. The study did not involve endangered species but did include many bird species protected under the U.S. Migratory Bird Treaty Act; therefore, permission to handle and collect these birds was obtained under U.S. Fish and Wildlife Service Scientific Collecting Permits (#MB05120C-1 and #MB54075B-1) and a Minnesota Department of Natural Resources Salvage Permit (#20412). Animal procedures were also approved by the Institutional Animal Care and Use Committee at Oklahoma State University (#AG-17-6).

### Experimental trials to quantify human and scavenger removal of carcasses

To quantify and account for human removal and animal scavenging of bird carcasses between surveys, we conducted experimental removal trials at all buildings and in all monitoring seasons. To minimize variation in visual and olfactory cues available to scavengers, the vast majority of trials used fully intact carcasses that likely resulted from a collision during the previous inter-survey period; these birds were left in place for trials. A small number of trials used carcasses that were collected during previous surveys, stored in a freezer, and thawed prior to the trial; however, these were also fully intact with fresh plumage and no signs of decomposition. All birds were marked as removal trial carcasses by affixing a tag to one leg. In addition to recording the above data associated with collision surveys, we recorded a unique alphanumeric code to track the status of each trial carcass on subsequent surveys. Selection of carcasses for inclusion in removal trials was non-random and based on the need for an adequate sample of trial carcasses for each building and season. Typically, the first carcass found at each building in each season was left in place for a removal trial, and additional trial carcasses were selected on varying schedules for different buildings, depending on observed numbers of collision fatalities. For example, at buildings with few collision fatalities observed, a higher proportion of carcasses were left in place than at buildings with many fatalities. Preliminary observations from Project BirdSafe indicated that bird carcasses in downtown Minneapolis are primarily removed by humans. Nonetheless, we sought to avoid bias in removal estimates that arises through “swamping” of animal scavengers (i.e., using more trial carcasses than can be removed by scavengers) [40] by ensuring there was never more than one trial carcass in place at any individual building façade or 11 carcasses simultaneously in place across the study area. Notably, this maximum of 11 trial carcasses occurred only once on a morning we documented 48 bird collisions; thus, the number of trial carcasses we used was well below the maximum number of carcasses the scavenger community could potentially encounter on a single day. Trial carcasses included a variety of species commonly killed by window collisions and represented a range of colorations (from drably colored sparrows to brightly colored warblers and buntings) and body sizes and masses (from hummingbirds and warblers to doves and woodcocks).

Once removal trial carcasses were marked, surveyors noted their presence or absence on each successive morning survey up to seven days after trial initiation, at which point remaining carcasses were retrieved and stored in a freezer or discarded if the carcass had substantially decomposed. We followed scavenging definitions in [33]. Specifically, carcasses were considered present if all or some of the carcass remains were detectable in the same place, or if they had been moved, within the survey area (i.e., within ~5 m of the building). Carcasses were considered removed if no detectable remains persisted within the survey area.

### Experimental trials to quantify surveyor detection of carcasses

To quantify and account for imperfect detection of bird carcasses present during collision surveys, we conducted experimental surveyor detection trials for all buildings and seasons. For each trial, a bird carcass collected in the current study, during Project BirdSafe, or incidentally outside of formal monitoring, was tagged on one leg with a unique alphanumeric code identifying it as a detection trial carcass, and placed by the trial coordinator (a technician or one of the authors) within a building’s survey area 0.5–1 hr before the start of a survey. Locations for trials were selected non-randomly to ensure adequate replication for each season and to capture a variety of surfaces on which carcasses were found (e.g., rocks, bare soil, pavement). Carcasses were also selected non-randomly to capture a range of colorations and body sizes/masses similar to that captured in the removal trials. At each trial location, a carcass was placed on the ground with the ventral side downward to conceal the tag. Throughout the study, surveyors were reminded that detection trials could occur at any time, but only the trial coordinator was aware of the date and location of specific trials. Upon encountering a detection trial carcass, surveyors picked it up, recorded the identification code, and alerted the trial coordinator that they had found it. When a detection trial carcass was not found, the trial coordinator returned to the placement location to determine if it had been removed. If the carcass was still present, we determined the surveyor had failed to detect it, but if the carcass was removed, we assumed it was unavailable for surveyors to detect and excluded the trial from detection rate calculations. Trial carcasses that were found were either disposed of, or if still in good condition, collected for reuse in future detection trials.

### Measuring potential correlates of bird-building collisions

We measured several variables to assess factors influencing bird-building collisions. For all building façades (i.e., discrete faces of buildings oriented in different directions), we used ImageJ [41] to measure glass area (including windows and other glass surfaces) based on digital photographs with a known-length reference object and taken in the daytime at an angle as close to perpendicular as possible to minimize image distortion. We calculated total glass area for each building by summing façade-level measurements. We also used ImageJ to estimate the area of each building’s windows that emitted light at night. We took at least three digital photographs of each building façade during nighttime hours, with at least one photo taken on a weekday and one taken on a weekend. All photos were taken at least one hour after sunset between 2045 and 2345 hr from 5 Sep 2017 to 5 Sep 2018. For each image, we calculated the area of windows that emitted any light. Because we observed night-to-night lighting variation, we averaged lighted area estimates across all dates for each building. We also generated an estimate of the proportion of building glass lighted (hereafter “proportion lighted”) by dividing lighted window area by total glass area. Finally, we characterized building height and horizontal ground area (i.e., footprint) because these size-related factors have previously been shown to influence collisions [14]. For height, we obtained maximum building height from either publicly accessible online sources (for 18 buildings) or using the 3D Building layer and 3D path ruler in Google Earth Pro 7.3.2.5491 (for 3 buildings). We used a building polygon shapefile in ArcGIS 10.1 [42] to calculate building footprints.

In addition to the above building features, we calculated three variables representing the interaction between buildings and their surrounding environment. We used ArcGIS 10.1 and 1-m resolution 2015 land cover data for the Twin Cities region [43] to estimate the distance of each building to the Mississippi River based on building centroids. We used this same land cover data to estimate the proportion of land covered by vegetation—including grass, shrubs, and deciduous and coniferous tree canopy; and excluding bare soil, other buildings, and roads and other paved surfaces—within 50 and 100 m of the outer edge of each building. These distance buffers were selected because previous literature has suggested vegetation cover within 50 m can influence bird-building collisions [14], because we also sought to consider potential effects of vegetation cover at a scale broader than that captured by the 50 m buffer, and because buffers larger than 100 m overlapped substantially due to the proximity of many buildings to each other. Substantial land cover changes have occurred in areas surrounding the stadium since its construction began in 2015, the most recent year for which high-resolution land cover data were available. To incorporate these changes in calculations of vegetation cover proportions, we used ArcGIS’s built-in aerial imagery base map, which reflected land cover in Jan 2018, and we manually digitized a polygon shape file of new land covers near the stadium. We converted this shape file to a raster file and merged it with the 2015 land cover layer with the ArcGIS “mosaic to new raster” tool.

### Bias-adjusted fatality estimates

We generated bias-adjusted collision fatality estimates and conducted statistical analyses in R version 3.6.1 [44]. For each building, we first calculated raw counts of both fatal and non-fatal collisions across all morning, midday, and evening surveys. We generated low and high counts based on exclusion or inclusion, respectively, of birds potentially resulting from predation events (for fatal collisions) or collisions with skyways (for fatal and non-fatal collisions). We used fatal collision counts to generate adjusted fatality estimates that account for human and scavenger removal of carcasses between surveys and for observer detection probability of carcasses present during surveys. We generated these bias-adjusted estimates using the GenEst statistical estimator [45], which allows modeling of carcass persistence and detection probabilities as a function of one or more covariates. This estimator also accounts for varying time intervals between surveys when estimating carcass persistence probability, which allowed us to account for: (1) missed surveys due to the above-described access issues for some buildings and days (a survey was considered missed if ≥50% of the building perimeter was not surveyed), and (2) varying time intervals between successive surveys for days when only morning surveys were conducted versus days when morning, midday, and evening surveys were conducted.

Using GenEst and data from carcass removal trials, we modeled carcass persistence probability for each building, and we treated the substrate on which trial birds were placed as a covariate (categories: rocks; natural, including grass, mulch, planters, and bare soil; and artificial, including concrete, metal, and other artificial surfaces) because the surface a bird lands on after colliding should influence the rate of detection and removal, especially by humans [33, 46]. Using data from surveyor detection trials, we modeled observer detection probability. Estimation of observer detection probability in GenEst includes the parameter *k*, which is the change in searcher efficiency with each successive search (range of *k* = 0–1; 0 represents a scenario where carcasses missed on the first trial cannot be found on a successive survey; 1 represents a scenario where searcher efficiency stays constant regardless of carcass age and/or the number of times a carcass was missed). GenEst estimates *k* if carcasses are left in place for surveyors to detect on subsequent trials; however, since we collected carcasses after all detection trials, we set *k* = 0.9, which represents an assumption that carcasses are detectable after each day but with slightly reduced detectability due to deterioration. We again treated substrate as a covariate but did not generate building-specific observer detection estimates because we had limited replication at some buildings, and there was no evidence suggesting that detection was influenced by building-related factors other than the surrounding substrates. Estimates of carcass persistence and observer detection probability were combined to model building- and substrate-specific estimates—along with 95% confidence intervals (CIs)—of the overall probability that a bird carcass resulting from a fatal collision was detected on the following survey. We generated adjusted fatality estimates by dividing both low and high raw counts of fatal collisions by the detection probability estimates for each building, with weighting to account for the proportion of each substrate in the survey area around each building. This procedure resulted in both low and high bias-adjusted fatality estimates (and associated 95% CI’s) for each building. Data used for GenEst bias-adjusted fatality estimates are in S1 Dataset; metadata and additional documentation for GenEst analyses are in S1 Appendix.

Notably, bias-adjusted estimates did not incorporate non-fatal collisions because removal and detection rates for live birds are likely different than for dead birds and it was infeasible to conduct removal and detection trials with live birds. Nonetheless, to present the full number of collisions, we summarized low and high raw counts of non-fatal collisions for each building. We also summarized numbers of carcasses found and submitted by stadium staff; however, we note that removal trials and bias-adjusted estimates should account for these birds under the assumption that staff were equally likely to remove birds marked for removal trials and those that collided but were not included in trials (see Results for validation of this assumption).

### Statistical analyses of factors influencing collision fatalities and numbers of species colliding

We only analyzed how fatal collisions were influenced by building-related factors because there was a strong correlation between low raw counts of fatal collisions and low raw counts of non-fatal collisions (Pearson’s r = 0.90) and also between high raw counts of fatal and non-fatal collisions (r = 0.89). This indicates that results should remain unchanged regardless of whether fatal or non-fatal collisions are assessed. The low raw count of fatal collisions and high raw count of fatal collisions were also strongly correlated (r = 0.99), so we based analysis on low fatal collision counts (i.e., those excluding potential predation events and skyway collisions; hereafter, low raw counts). We also conducted an analysis using bias-adjusted fatality estimates to determine if correlates differed from the raw count analysis. We based this analysis on the high adjusted estimates of fatal collisions (hereafter, high adjusted estimates) because these were not as strongly related to the low raw counts (r = 0.85) as the low adjusted estimates were to the low raw counts (r = 0.94). For analyses of both low raw counts and high adjusted estimates, we used generalized linear models (GLMs) with a negative binomial error distribution (function “glm.nb” in the MASS package) because preliminary analyses indicated that fatality count data were over-dispersed, and likelihood ratio tests showed that negative binomial models fit the data significantly better than poisson models. In addition to analyzing factors influencing total collision fatalities, we also conducted separate analyses for fatalities in spring and fall, and for total fatalities across seasons for each of the five most frequently colliding bird species (see Results). These season- and species-specific analyses were also conducted using negative binomial GLMs, and we used low raw counts because we did not have enough removal and detection trial replicates at each building to generate bias-adjusted estimates by season and species. Finally, and again using negative binomial GLMs, we assessed factors influencing the number of species colliding at each building (i.e., counts of numbers of species, not fatalities), including across the entire study and separately for spring and fall. This analysis combined fatal and non-fatal collisions because numbers of species fatally colliding was strongly correlated with total species colliding (r>0.99). For all analyses, collision response variables included data for both 2017 and 2018 because there was no significant difference between years in either fatal collisions (*t* = -1.86; df = 20; *p* = 0.08) or total collisions (*t* = -1.70; df = 20; *p* = 0.11) at each building.

For all analyses, we began with an initial set of eight predictor variables (building height, glass area, lighted window area, proportion lighted, footprint, distance to Mississippi River, and proportion of land covered by vegetation within 50 m and 100 m). Preliminary analyses indicated strong correlations (r>|0.7|) between three variable pairs: glass area and building height (r = 0.75); lighted window area and footprint (r = 0.85); and percent vegetated cover within 50 and 100 m (r = 0.80) (S1 Table). To avoid multicollinearity, we only retained the variable from each pair that was more strongly correlated to the response variable of interest. Glass area and lighted window area were correlated with each other, but just below the 0.7 criterion (r = 0.698); we retained both variables for analysis because few previous studies have separately considered the role of these two factors. Following removal of correlated variables, we used the “stepAIC” function in the R package “MASS” to implement a backwards elimination approach to model selection, beginning with a global additive model (i.e., containing all uncorrelated variables), which retained variables when their removal resulted in an increase in ΔAIC of greater than 2. For variables included in the top model selected using this procedure, we also assessed model coefficients, and we only drew inferences from variables that had non-standardized coefficient estimates with 95% confidence intervals that did not overlap zero. All data used for statistical analyses are in S2 and S3 Datasets, and R code for analyses is in S2 and S3 Appendices.

## Results

### Raw counts and species composition of collisions

Across all buildings, seasons, and species, the low raw count (excluding possible predation events and skyway collisions) was exactly 1,000 fatal and non-fatal bird collisions (per building range = 2–305 total collisions) (Table 2). The vast majority of these (86.8%) were found during morning surveys, of which we conducted far more (372 surveys) than midday (58 surveys; 7.8% of collisions) and evening surveys (57 surveys; 5.4% of collisions). Four buildings including the stadium caused 74.3% (743) of collisions. Of all collisions, 22% (220) were non-fatal (i.e., birds we found stunned and/or saw fly away; per building range = 0–70 non-fatal collisions) and 78% (780) were fatal (i.e., carcasses or remains; per building range = 1–254 fatal collisions); the same four buildings caused 74.0% (577) of all fatal collisions. Across both years, we observed nearly four times more collisions in fall (758) than spring (209), with 33 collisions in June. Including an additional 167 collisions (153 fatal; 14 non-fatal) that were potential predation events and skyway collisions resulted in a high raw count of 1,167 collisions; the same building rankings and seasonal patterns also emerged for high counts.

**Table 2:** Collision counts, results of removal and detection trials, and bias-adjusted fatality estimates for all buildings.

	Raw counts^b^			Bias trials^d^			Bias-adjusted fatalities^e^	
Building Id^a^	Fatal	Non-fatal	# of species^c^	Removal	Detection	Detection prob.	Low	High
17	254–264	51–51	44	43	5	0.59 (0.48–0.69)	431 (370–525)	448 (384–545)
4	91–113	27–30	38	32	6	0.31 (0.18–0.45)	297 (202–493)	369 (251–613)
1 (Stadium)	155–159	70–70	42	27	23	0.70 (0.56–0.80)	222 (192–274)	228 (197–281)
3	77–112	18–20	35	33	1	0.48 (0.36–0.61)	158 (126–211)	231 (184–307)
8	4–8	0–1	4	5	2	0.04 (0.00–0.62)	114 (6–4000)	228 (12–8000)
9	59–64	8–10	24	24	14	0.70 (0.58–0.81)	83 (72–102)	90 (78–111)
19	29–34	4–4	17	14	3	0.43 (0.25–0.62)	67 (46–115)	79 (54–135)
12	25–26	5–7	13	10	4	0.53 (0.33–0.72)	47 (34–76)	48 (36–79)
20	23–28	9–10	15	9	2	0.51 (0.28–0.72)	45 (32–83)	54 (39–101)
15	11–15	5–5	9	11	1	0.29 (0.14–0.50)	37 (21–76)	51 (29–104)
13	6–8	4–4	9	8	4	0.24 (0.08–0.50)	24 (12–72)	32 (16–96)
2	9–18	1–3	9	6	3	0.39 (0.18–0.64)	22 (13–50)	45 (27–101)
6	14–45	4–4	10	14	4	0.64 (0.48–0.79)	21 (17–29)	70 (57–94)
16	1–1	1–1	1	6	1	0.05 (0.00–0.61)	20 (1–1000)	20 (1–1000)
21	5–7	2–2	5	8	9	0.45 (0.24–0.69)	11 (7–20)	15 (10–29)
5	5–11	5–6	6	8	6	0.65 (0.44–0.81)	7 (6–11)	16 (13–24)
10	4–4	3–3	6	5	6	0.56 (0.24–0.80)	7 (4–16)	7 (4–16)
11	3–6	2–2	5	7	2	0.51 (0.24–0.74)	5 (4–12)	11 (8–24)
14	1–6	1–1	2	3	3	0.18 (0.02–0.65)	5 (1–58)	33 (9–350)
7	2–2	0–0	2	5	3	0.57 (0.29–0.78)	3 (2–7)	3 (2–7)
18	2–2	0–0	2	8	3	0.61 (0.37–0.79)	3 (2–5)	3 (2–5)
Totals	780–933	220–234	75	286	105	-	1629 (1170–7235)	2081 (1413–12022)

Collision counts, results of removal and detection trials, and bias-adjusted bird fatality estimates for 21 buildings, including U.S. Bank Stadium, monitored in downtown Minneapolis, Minnesota, 2017–2018. Table includes raw counts of fatal and non-fatal collisions; information about bias trials that were used to generate detection probability estimates accounting for both carcass removal and imperfect detection of window-killed bird carcasses; and bias-adjusted fatality estimates based on application of detection probability estimates to raw fatal collision counts. Buildings are ranked in descending order based on the low bias-adjusted fatality estimate (parentheses indicate 95% confidence intervals).

^a^Unique numeric code for each building used for purposes of current study.

^b^Raw counts for fatal and non-fatal collisions at each building; low and high values are counts that respectively exclude and include birds potentially resulting from predation events (for fatal collisions) and collisions with skyways between buildings (for fatal and non-fatal collisions).

^c^Number of species observed as collision casualties across the entire study, including both fatal and non-fatal collisions.

^d^Number of carcass removal trials conducted to quantify animal scavenger and human removal of carcasses, number of detection trials conducted to quantify surveyor detection probability for carcasses present in search area (excludes detection trials where trial carcasses were removed before surveyors had a chance to encounter them), and estimated probability of detecting a window-killed carcass that falls in the survey area (detection probability accounts for both removal and detection probability).

^e^Bias-adjusted fatality estimates based on application of detection probability estimates to raw fatal collision counts; low and high adjusted estimates were generated using the low and high fatal collision counts.

**Fig 2:**
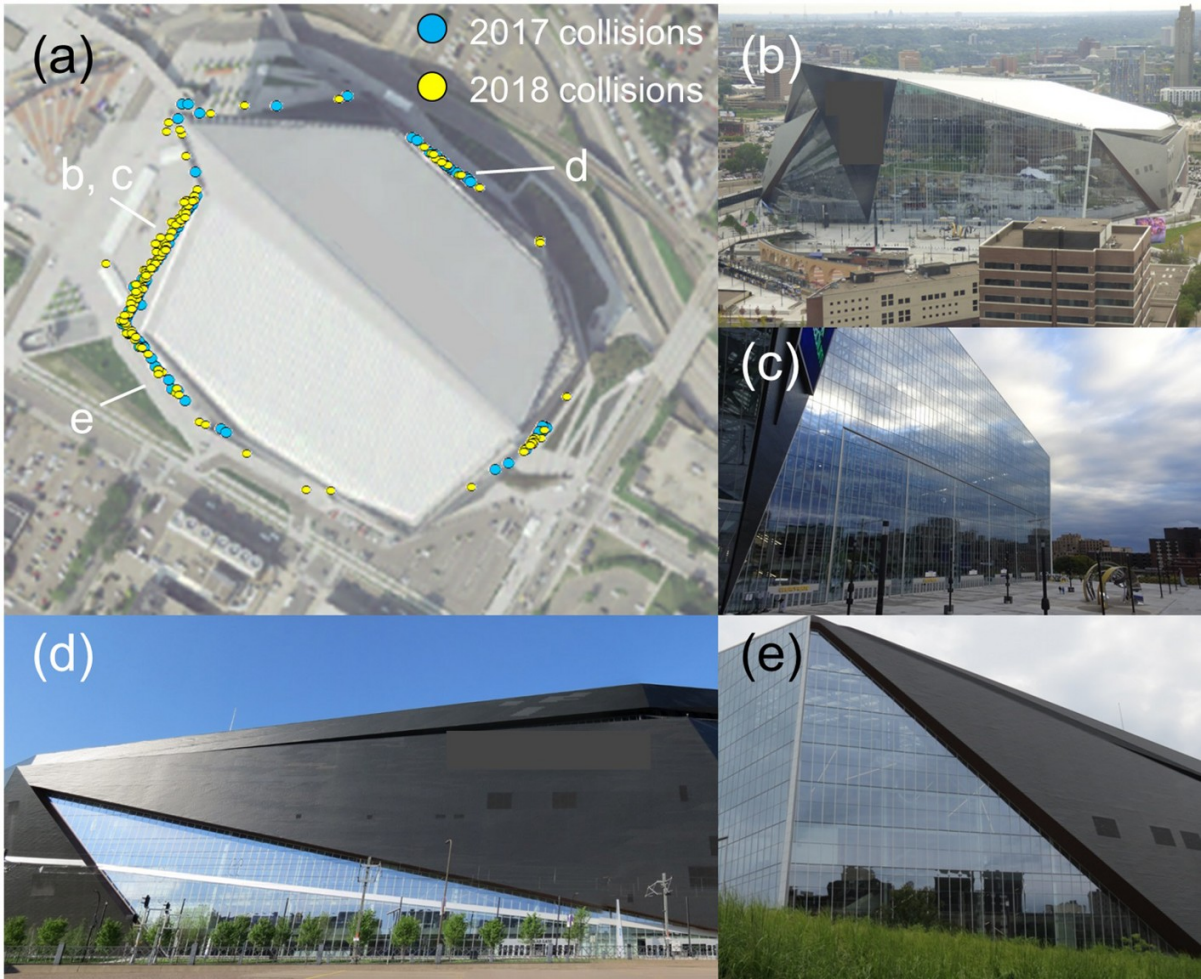
Bird collisions at U.S. Bank stadium. (a) Locations of 229 bird collisions (159 fatal collisions; 70 non-fatal collisions; 95 collisions in 2017; 134 in 2018) observed during monitoring at U.S. Bank Stadium in downtown Minneapolis, Minnesota, USA, 2017–2018; Points include carcasses potentially resulting from predation events and bird collisions with skyways (i.e., the high raw counts described in the text). (b, c) the largest unbroken span of glass (~6,000 m^2^) where 52% of all collisions at the stadium occurred; (d) a glass surface on the northeast façade where 11% of collisions occurred; (e) a glass surface on the southwest façade where 17% of collisions occurred. Image sources: USGS National Map Viewer NAIP Plus aerial imagery (a); the authors (b-e).

In addition to a few buildings causing the majority of collisions, a small number of façades caused most of the collisions at several buildings. For example, we documented collisions around the stadium’s entire perimeter, but 52% of all collisions occurred at the ~6,000 m^2^ expanse of glass on the northwest façade, 17% occurred at one glass surface on the southwest façade, and 11% occurred at one glass surface on the northeast façade (Fig 2). In addition to collisions observed at the stadium during surveys, 62 bird carcasses (20 in 2017; 42 in 2018) were submitted by stadium staff. Supporting our assumption that staff removed birds marked for removal trials at a rate similar to carcasses not in trials—and therefore that removal trials accounted for staff-removed birds—5 of the 62 submitted carcasses were removal trial birds.

Among the 1,000 collision records that excluded possible predation events and skyway collisions, we identified 75 bird species as collision casualties (per building range = 1–44; Table 2), including 72 fatally injured species (per building range = 1–37). Five species accounted for 48.9% of all collisions: White-throated Sparrow (*Zonotrichia albicollis*) (14.1%), Nashville Warbler (*Leiothlypis ruficapilla*) (10.8%), Ovenbird (*Seiurus aurocapilla*) (9.8%), Common Yellowthroat (*Geothlypis trichas*) (7.4%), and Tennessee Warbler (*Leiothlypis peregrina*) (6.8%) (Table 3; see S2 and S3 Tables for counts of all species overall and by season). The same species were the top colliders in fall, although Ovenbird and Common Yellowthroat switched the third and fourth rankings. During spring, Ovenbird, White-throated Sparrow, Tennessee Warbler and American Woodcock (*Scolopax minor*) were the top four colliders, followed by three species tied for fifth: Black-billed Cuckoo (*Coccyzus erythropthalmus*), Northern Waterthrush (*Parkesia noveboracensis*), and Dark-eyed Junco (*Junco hyemalis*).

**Table 3:** Top ten most frequently colliding bird species.

All seasons		Spring (15 Mar-31 May)	
Species	Count	Species	Count
White-throated Sparrow	141	Ovenbird	37
Nashville Warbler	108	White-throated Sparrow	34
Ovenbird	98	Tennessee Warbler	15
Common Yellowthroat	74	Unknown bird^a^	13
Tennessee Warbler	68	American Woodcock	8
Dark-eyed Junco	33	Black-billed Cuckoo	7
Unknown bird^a^	32	Northern Waterthrush	7
Black-and-white Warbler	29	Dark-eyed Junco	7
Ruby-throated Hummingbird	26	Black-and-white Warbler	6
Northern Waterthrush	22	Yellow-bellied Sapsucker	5
Summer (1–30 Jun)		Fall (15 Aug-31 Oct)	
Species	Count	Species	Count
House Sparrow	6	White-throated Sparrow	107
Black-billed Cuckoo	5	Nashville Warbler	104
Yellow-billed Cuckoo	4	Common Yellowthroat	66
House Finch	4	Ovenbird	61
Common Yellowthroat	3	Tennessee Warbler	53
Unknown bird^a^	2	Dark-eyed Junco	26
Chipping Sparrow	1	Ruby-throated Hummingbird	23
Virginia Rail	1	Black-and-white Warbler	23
Mourning Warbler	1	Lincoln's Sparrow	19
Red-eyed Vireo	1	Red-breasted Nuthatch	18

Top ten most frequently colliding bird species (includes fatal and non-fatal collisions) across all collision surveys at all 21 monitored buildings, including U.S. Bank Stadium, in downtown Minneapolis, Minnesota, USA, 2017–2018. The “All seasons” count excludes mid-summer and winter periods when no collision monitoring occurred (see S2 and S3 Tables for counts of all species observed as collision casualties, including overall and by season, respectively).

^a^Birds that could not be identified to any taxonomic level, typically due to dismemberment and/or severe decomposition, distant viewing, and/or poor quality documentation photos.

### Bias-adjusted fatality rates and comparisons among buildings

We conducted 286 removal trials and 105 surveyor detection trials, not counting detection trials where a carcass was removed before a surveyor could detect it. Combining GenEst-derived estimates of carcass persistence probability (which was modeled for each building and as a function of substrate) and observer detection probability (which was modeled across buildings and as a function of substrate), resulted in overall estimates of detection probability that varied among buildings from 4% to 70% (mean = 45%).When applying building-specific detection probabilities to fatal collision counts, we generated bias-adjusted fatal collision estimates that varied among buildings from 3 to 431 (median = 24; mean = 78) based on low fatality counts and 3 to 448 (median = 48; mean = 99) based on high fatality counts. Based on low adjusted estimates, the stadium had the third highest fatality estimate behind buildings #4 and #17. Based on the high adjusted estimates, the stadium ranked fourth behind buildings #3, #4, and #17. When adding non-fatal collisions (either low or high counts) to any bias-adjusted estimates, the stadium always ranked third in total collisions behind #4 and #17.

### Factors influencing collision fatalities and numbers of species colliding

**Fig 3:**
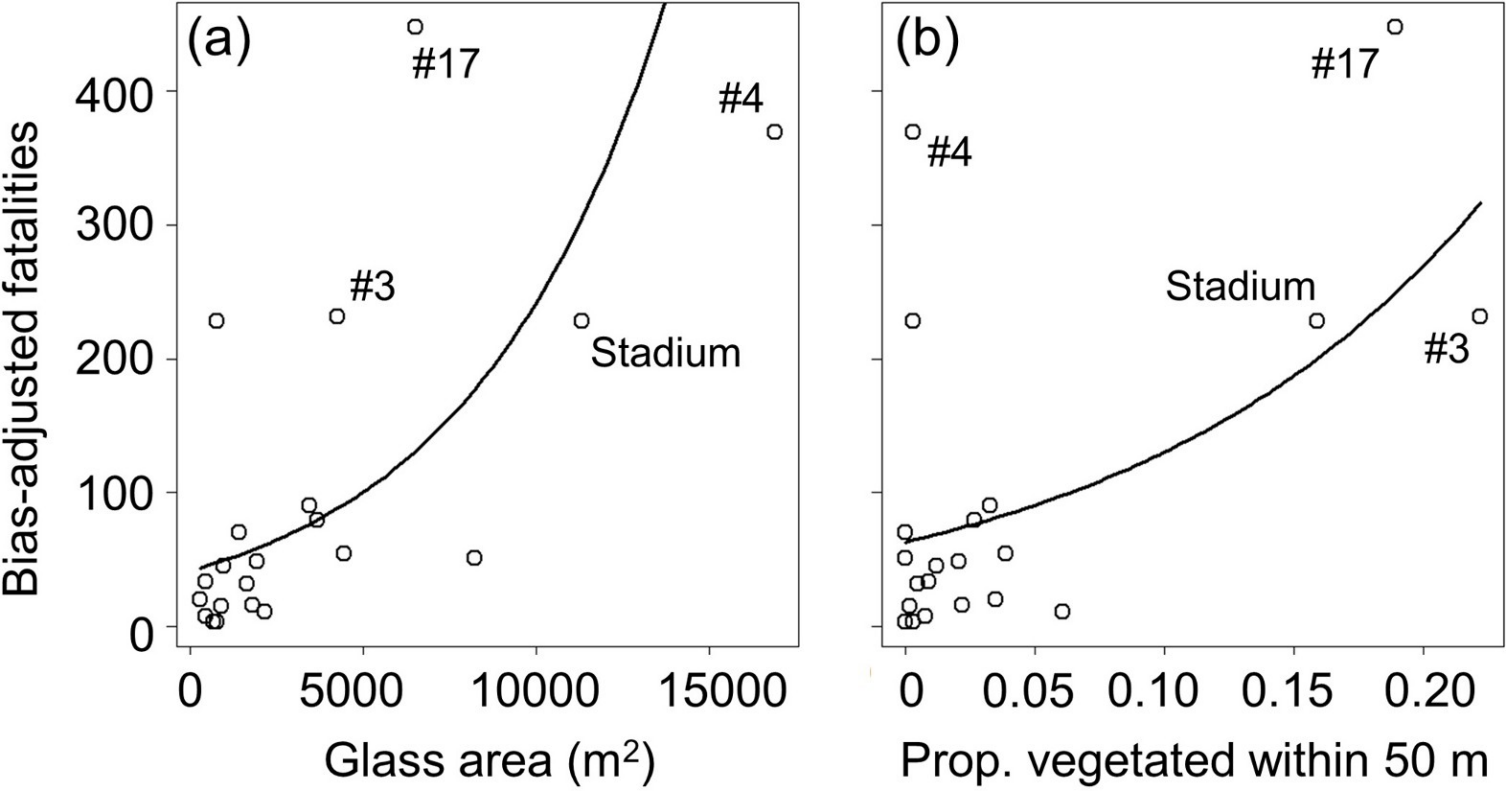
Correlates of numbers of collision fatalities (all buildings). Relationships between high bias-adjusted estimates of bird collision fatalities (see text for description of this fatality estimate) and (a) glass area, and (b) proportion of land covered by vegetation within 50 m. The four buildings estimated to cause the greatest numbers of fatalities, including the stadium, are labelled (numbers represent unique numeric codes used for purposes of current study); For results based on 17 buildings with these 4 potential outliers removed, see text and S1 Fig.

**Fig 4:**
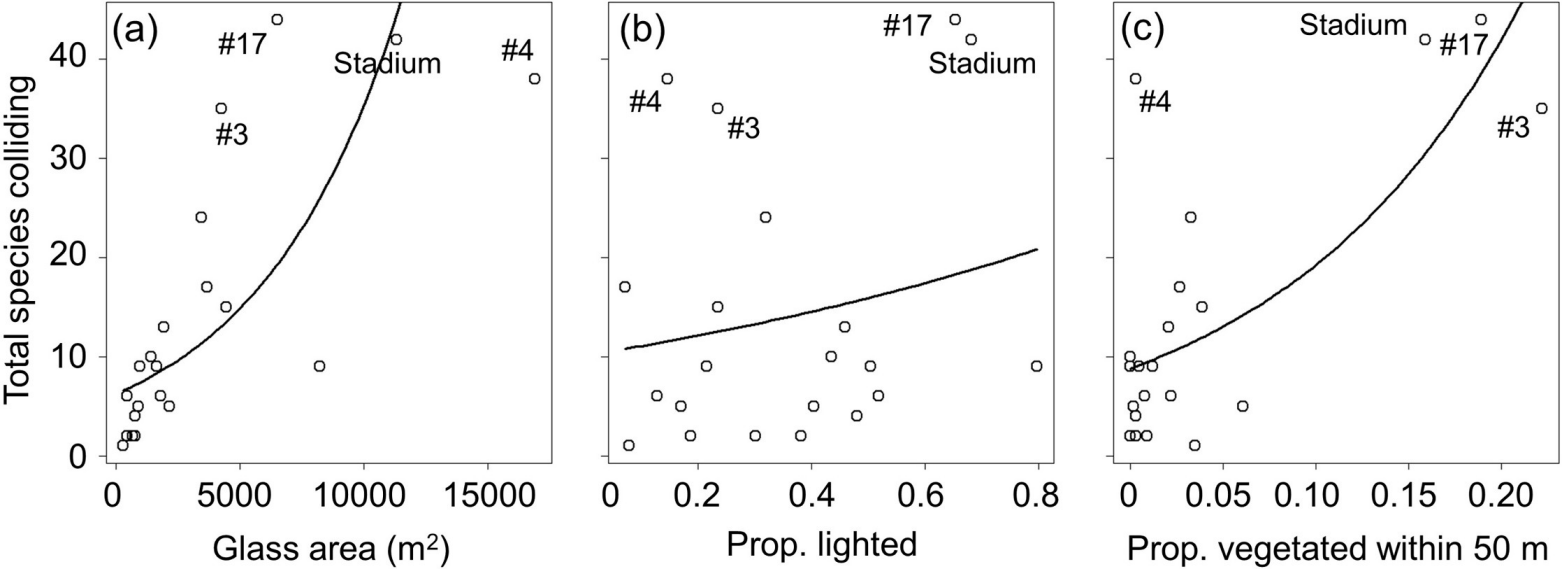
Correlates of numbers of species colliding (all buildings). Relationships between total numbers of species observed as casualties (including both fatal and non-fatal collisions) and (a) glass area, (b) proportion of glass area with lighting emitted at night, and (c) proportion of land covered by vegetation within 50 m. The four buildings estimated to cause the greatest numbers of collisions, including the stadium, are labelled (numbers represent unique numeric codes used for purposes of current study); For results based on 17 buildings with these 4 potential outliers removed, see text and S2 Fig.

After excluding variables that appeared in top models but had non-standardized coefficients with 95% CI’s overlapping zero, the top model for most collision variables included only glass area and proportion of vegetated cover within 50 m (standardized coefficient estimates for strongly supported variables in Table 4). In all instances, these factors had a positive effect (i.e., increasing collisions with increasing glass area and vegetation), including for total low fatality counts, high adjusted fatality estimates (Fig 3), spring and fall fatalities, and fatalities for the three most frequently colliding species (White-throated Sparrow, Nashville Warbler, Ovenbird). For Common Yellowthroat, the top model included positive effects of glass area and vegetation within 100 m, and for Tennessee Warbler, the top model included only a positive effect of vegetation within 50 m. For total numbers of species colliding, the top model included positive effects of glass area and vegetation within 50 m, as well as a positive effect of the proportion of glass lighted at night (Fig 4). The top model for numbers of species colliding in spring and fall also included positive effects of glass area and vegetation (within 100 m for spring; 50 m for fall), and the model for spring also included a positive effect of proportion lighted. For most response variables, standardized coefficient estimates (Table 4) illustrated that effects of glass area and vegetation were of approximately similar magnitude when both factors were supported. The effect of proportion lighted was slightly less than effects of glass area and vegetation for the response variables with all three factors supported.

**Table 4:** Standardized coefficient estimates for variables in supported models for analyses including all 21 buildings.

							Prop. vegetation	
	Height	Glass area	Prop. light	Area light	Footprint	Distance to river	50 m buffer	100 m buffer
*Collision fatalities (all)*								
Total low raw count^a^	-	0.012	-	-	-	-	0.012	-
Total high adj. estimate^b^	-	0.005	*0*.*003*	-	-	-	0.003	-
Spring low raw count^c^	-	0.036	-	-	-	-	0.048	-
Fall low raw count^d^	-	0.019	-	-	-	-	0.016	-
*Collision fatalities (species)*^*e*^								
White-throated Sparrow	-	0.051	-	-	-	-	0.089	-
Nashville Warbler	-	0.113	-	-	-	*-0*.*054*	0.107	-
Ovenbird	-	0.096	-	-	-	-	0.093	-
Common Yellowthroat	-	0.110	*0*.*041*	-	-	-	-	0.169
Tennessee Warbler	-	-			-	-	0.230	-
*Number of species*^*f*^								
All seasons	-	0.066	0.033	*-0*.*042*	*-*	*-0*.*020*	0.039	-
Spring	-	0.120	0.090	*-0*.*075*	-	-	-	0.117
Fall	-	0.049	-	-	-	-	0.049	-

Standardized coefficient estimates for variables included in strongly supported models for analyses of building-related variables associated with bird collisions based on monitoring at 21 buildings, including U.S. Bank Stadium, in downtown Minneapolis, Minnesota, USA, 2017–2018. Analyses were conducted for total collision fatalities across all seasons and for spring and fall, for total fatalities for the five species most frequently observed as collision casualties, and for numbers of species observed to collide across all seasons and for spring and fall. For results based on subset of 17 buildings with potential outliers excluded (stadium, #3, #4, and #17), see text and S4 Table. Coefficients in italics had non-standardized coefficient estimates with 95% CI’s that overlapped zero.

^a^Analysis response variable was raw counts of total fatal collision casualties excluding birds potentially resulting from predation events and collisions with skyways connecting buildings.

^b^Analysis response variable was bias-adjusted estimates of fatal collisions adjusted to account for removal of bird carcasses by humans and animal scavengers and for imperfect detection of carcasses present during surveys (this version of the bias-adjusted estimate was based on the high raw count of fatal collisions, which included birds potentially resulting from predation events and collisions with skyways connecting buildings).

^c^Analysis response variable was raw counts of spring fatal collision casualties excluding birds potentially resulting from predation events and collisions with skyways connecting buildings.

^d^Analysis response variable was raw counts of fall fatal collision casualties excluding birds potentially resulting from predation events and collisions with skyways connecting buildings.

^e^Analysis response variables were low raw counts of fatal collision casualties for individual species, excluding birds potentially resulting from predation events and collisions with skyways connecting buildings.

^f^Analysis response variables were total numbers of identifiable species observed as fatal and non-fatal collision casualties at each building.

Visual inspection of the above relationships (Figs 3 and 4) suggests that four large buildings with extensive glass area and/or nearby vegetation (#3, #4, #17, and the stadium) largely drove the importance of these factors for nearly all analyses. To determine if additional factors influence collisions for a set of buildings more representative of most of those in downtown areas, we removed the above four buildings and re-ran analyses (data used for these analyses are in S3 Dataset, and R code is in S3 Appendix). For this subset of 17 buildings, only glass area and lighted window area were correlated (r = 0.77); because there was another variable that captured lighting (proportion lighted), we removed lighted window area from these analyses to avoid multicollinearity. For the subset of 17 buildings, and after setting aside variables that had non-standardized coefficients with 95% CI’s overlapping zero, the top model for total low fatality counts included positive effects of glass area and vegetation within 100 m (S1 Fig; standardized coefficient estimates in S4 Table). The model for the high adjusted estimates did not converge, even when manually changing the number of model iterations (possibly due to low replication relative to the broad range of fatality estimates); therefore, we were unable to identify correlates for this response variable for the subset of 17 buildings. The top model for spring fatalities included positive effects of proportion lighted and vegetation within both 50 m and 100m. The top model for fall fatalities included glass area and vegetation within 100 m. For both White-throated Sparrow and Ovenbird, the top model included only positive effects of glass area, and the top model for Common Yellowthroat contained this same effect and positive effects of vegetation within 50 and 100 m. The top model for Nashville Warbler included positive effects of building height, building footprint, and vegetation within 50 m, and for Tennessee Warbler, the null model ranked highest, indicating that none of the variables we measured explained collision fatalities for this species. The top model for total numbers of species colliding included positive effects of glass area, proportion lighted, and vegetation within 100 m (S2 Fig). The top model for species colliding in spring included positive effects of proportion lighted and vegetation within 100 m, and the model for fall included positive effects of glass area and vegetation within 100 m.

## Discussion

In a study of 21 buildings over four migration seasons in downtown Minneapolis, Minnesota, we documented substantial variation among buildings in numbers of bird collisions, with four large buildings causing the majority of collisions, including a large multi-use stadium, which ranked third for most estimates. These same four buildings drove the positive effects of glass area and the proportion of surrounding land covered by vegetation on nearly all collision response variables. Focusing on 17 buildings more representative of most of those in major downtown areas resulted in slightly different predictors of collisions emerging, which suggests that factors leading some buildings to cause exceptionally high numbers of bird collisions are not the exact same factors causing collision variation among a more typical set of buildings. Across both sets of analyses, we also found evidence that the proportion of glass lighted at night influences bird collision fatalities in spring, as well as the number of species colliding overall and in spring.

### Collision comparisons among buildings

Collision numbers varied greatly among buildings, with four buildings (three high-rise office buildings and U.S. Bank stadium) causing 74% of observed fatal collisions (based on low raw fatality counts) and 68% of estimated fatalities (based on low bias-adjusted estimates). Estimated fatality rates for these top buildings, which ranged from 79 to 216 fatalities/yr (111 fatalities/yr at the stadium), not only exceeded other buildings in this study, but also exceed the estimated range of fatality rates at the majority of U.S. high rise buildings (5–77 birds/yr as estimated with collision data from 11 cities) [4]. Fatality rates exceeding those of our top buildings have in some cases been shown at other extremely large and/or glassy buildings such as: the McCormick Place Convention Center in Chicago, Illinois (four inter-connected buildings along the Lake Michigan shoreline with an average of 1,028 fatalities/yr from 1978 to 2012) [4, 47]; the Yonge Corporate Centre in Toronto, Canada (three office buildings with >800 fatalities in 2010) [48], and the vehicle assembly building at the John F. Kennedy Space Center in Florida (a 160 m tall, 32,376m^2^, mostly windowless structure with an average of 421 fatalities/yr from 1980 to 1991) [49]. These examples, as well as the top-ranked buildings in our study, seem to represent some of the highest bird collision fatality rates documented to date; indeed, these types of buildings were excluded from a U.S. estimate of bird-building collision mortality due to their high outlier status [4]. Ours and the above studies indicate that major bird collision reductions can be achieved by focusing mitigation efforts on a small number of especially problematic buildings.

We are unaware of other collision studies at stadiums; thus, direct comparisons between U.S. Bank Stadium and other similar structures are not yet possible. Nonetheless, given research showing that large, glassy buildings nearly always cause large numbers of bird collisions, we expect that similar glassy stadiums would also cause substantial collision mortality. Even less-glassy stadiums with extensive lighting may cause numerous collisions because intense nighttime lighting confuses nocturnally migrating birds, altering their flight paths, bringing them closer to the ground, and elevating collision risk [27]. Recognizing the risk posed to birds, there have been some efforts to incorporate bird-friendly design elements into new stadiums. For example, the Fiserv Forum basketball arena in Milwaukee, Wisconsin, was designed to reduce bird collisions by minimizing the use of reflective and see-through glass [50]. Retroactive treatment of existing stadiums should also reduce collisions, and regardless of the approach used—whether it be installation of bird-friendly glass, application of film, markers, netting, or other materials over existing glass, or some other approach—in-field monitoring and validation of the effectiveness of different approaches is needed to clarify which mitigation steps work best for different types of glass, buildings, and surroundings (e.g., heavily vegetated vs. non-vegetated). Notably, our results for U.S. Bank Stadium suggest that a major reduction in collisions can be achieved by focusing mitigation on one or more particularly problematic spans of glass (Fig 2).

Although we accounted for removal of carcasses by humans and scavengers, as well as imperfect detection of carcasses present during surveys, the true number of fatalities was greater than our bias-adjusted estimates. These estimates only represent the monitoring period (15 Mar-30 Jun; 15 Aug-31 Oct), and although collisions are less frequent in other seasons [16, 37], additional collisions undoubtedly occurred during unmonitored seasons at most buildings. We also missed an unknown number of non-fatal collisions where birds flew away before the next survey. An unknown number of these birds, and of non-fatal collisions we did observe, likely died later or experienced sublethal effects that impaired their behavior, susceptibility to predation, and/or ability to continue migration and eventually reproduce [51]. Notably, the percentage of such birds that survive is virtually unknown in the scientific literature due to difficulties of tracking birds after non-fatal collisions. Finally, at most buildings, additional bird carcasses likely fell in inaccessible locations, such as above-ground platforms and areas of roofs beneath windows.

### Factors influencing collision fatalities and numbers of species colliding

When considering all 21 buildings, glass area and vegetation within 50 m were included in top models for most collision response variables. Because buildings with extensive glass area also tended to be tall, and because buildings with extensive vegetation within 50 m also tended to have abundant vegetation within 100 m, we were unable to isolate the effects of these factors. Our results nevertheless suggest that large glassy buildings with extensive nearby vegetation or park space cause the greatest numbers of collisions. Past studies at a variety of building types have also shown increases in bird collisions with greater building height [4, 19], area and/or percentage of windows or glass [7, 19, 25], and vegetation near buildings [7, 11–12]. The effect of glass area likely arises due to several factors, including greater confusion of birds due to larger amounts of reflective and/or see-through surfaces, especially in large unbroken expanses [52], and an increase in light emission increasing numbers of nocturnal migrants attracted to buildings (see lighting discussion below). The effect of vegetation likely occurs due to its attractiveness to birds as a source of food and cover, especially for migratory birds resting and refueling during stopovers in an otherwise heavily urbanized landscape. Vegetation may also exacerbate reflection effects; birds may be less able to perceive glass as a barrier when it reflects vegetation and/or more likely to fly toward glass if they perceive they are flying toward vegetation [10].

Nearly all studies of bird-building collision correlates have assessed collisions across the entire monitoring period (usually spring and fall, or fall only) and for all birds combined. We provide evidence that collision correlates can vary among seasons and species, a conclusion supported by the limited past research that has assessed species-specific correlates [25]. When outlier buildings were excluded, spring fatalities were best predicted by proportion lighted and vegetation within 50 and 100 m, while fall fatalities and total fatalities were best predicted by glass area and vegetation within 100 m. For species analyses including all buildings, glass area and vegetation within 50 m were each supported for 4 of 5 species; however, Common Yellowthroat fatalities were predicted by vegetation within 100 m. A unique pattern also emerged for Nashville Warbler when outlier buildings were excluded; fatalities for this species were positively influenced by building height, footprint, and vegetation within 50 m. These results suggest that Nashville Warbler habitat use, flight behavior, and/or collision avoidance may be more closely tied to factors associated with building size than other species, and that Common Yellowthroat may be more likely to be attracted near buildings when nearby vegetation covers an area larger than that captured by a 50 m distance buffer. Finally, Tennessee Warbler was the only species for which fatalities were not associated with glass area and for which no variables predicted fatalities in the outlier-excluded analysis. These results suggest that factors other than glass area and the other variables we measured could influence collisions for this species. More broadly, the above types of species-specific collision correlates could also arise due to other biological and ecological factors that vary among species, including morphology (e.g., wing-loading) and flight maneuverability, migration timing (relative to both time of day and season), and visual capacity to detect reflective and transparent surfaces at different distances, during different times of day, and under different lighting conditions. Regardless of the mechanisms, our findings suggest that results of studies focused on one migration season, all seasons combined, and/or all birds combined should not necessarily be extrapolated across all seasons and species. Further, management measures based on correlates identified in such studies may not be equally effective for all species and seasons, and species- and season-specific approaches may be necessary to achieve the greatest reduction in collisions.

Factors associated with total numbers of species colliding were nearly identical to those influencing total collision fatalities. Both glass area and vegetation were associated with both response variables regardless of whether outlier buildings were included, although as discussed below, a positive effect of proportion lighted was also supported for numbers of species colliding. We are uncertain if these factors independently influence both numbers of fatalities and numbers of species colliding, or if they only explain number of species colliding because more species are represented by chance with a greater number of fatalities. We hypothesize that glass area and vegetation could directly influence numbers of species colliding; this could occur if large buildings with extensive glass and nearby vegetation attract a greater diversity of birds as a result of being surrounded by a greater diversity of land covers and/or vegetation that provides diverse food and cover. Past research provides evidence for this explanation; a study in Toronto found that forest-dwelling bird species collided more at buildings surrounded by extensive greenspace while open woodland-dwelling species collided more at buildings surrounded by extensive urbanization [25]. Thus, greater variation in land cover at large, glassy buildings could result in attraction and collision of a larger diversity of species with varying habitat affinities.

Notably, habitat loss is one of the greatest threats to bird populations, and as human development and urbanization expand, urban vegetation and greenspaces provide many benefits to birds, including resident birds and migratory birds passing through urban areas. However, our results are consistent with past research suggesting that vegetation near windows elevates collision rates. Taken together, these conclusions stress the need to prioritize mitigation strategies related to reducing window collisions (e.g., window films and markers) versus those reducing urban vegetation. Further, such collision mitigation steps may be most important for buildings and glass surfaces surrounded by extensive vegetation and greenspace.

Caution should be taken in interpreting our results, as the large number of analyses with assessment of variable importance based on 95% confidence intervals of coefficient estimates increases the risk of Type I error (i.e., apparently significant effects arising by chance alone). Further, although characteristics of the outlier buildings appear to influence which collision correlates were identified and therefore provide insight into collision risk factors for these structures, greater replication of large, glassy, and irregularly shaped buildings (including stadiums) would more conclusively identify bird collision risk factors that are generalizable to multiple contexts. This increased replication could be achieved through coordinated and standardized collision monitoring in multiple cities (e.g., following [14]), meta-analyses of published and unpublished datasets, and creation of a bird collision database to facilitate data sharing among researchers, conservation organizations, and building designers (see also [53]).

### Evidence for effects of nighttime lighting on bird-building collisions

The proportion of glass emitting light at night appeared in top models for spring collision fatalities (analysis excluding outliers) and numbers of species colliding overall and in spring (both all-building and outlier-excluded analyses). Lighted window area was not supported for any collision variables in the all-building analysis, and we did not include this factor in the outlier-excluded analysis because it was correlated with glass area. However, we expected lighted window area to also be associated with collisions because it was correlated with glass area—which predicted most collision variables—and because past studies have shown a positive relationship between bird-building collisions and a light emission index that is similar to lighted window area in combining building size with the percentage of buildings or windows emitting light [18, 54]. We tested this possibility by re-running all analyses either with glass area removed (all-building analysis) or replaced by lighted window area (outlier-excluded analysis); this resulted in lighted window area being included in the top model for nearly all collision variables. Nevertheless, we are unable to isolate the effects of these two factors because the buildings in our study that had extensive glass area also tended to have an extensive area of lighted windows at night.

We expected lighted window area to be relevant to bird collisions, as this factor should indicate the amount and/or brightness of light pollution birds experience near buildings, and thus the degree to which they are confused, disoriented, and/or attracted to buildings [29]. However, the apparent effect of proportion of glass lighted on some collision response variables was somewhat surprising because any given proportion value represents a different amount of light emission depending on building size and glass area. The proportion lighted variable could indirectly capture the contiguousness of lighted area on buildings; in other words, lighted areas may be closer together and/or occur in larger unbroken spans when proportions of lighting are greater. This increased contiguity of lighting could pose greater perceptual challenges to birds, such that they experience greater disorientation or attraction or have greater difficulty detecting and avoiding glass, an effect analogous to that of contiguous expanses of glass [52]. Future research could isolate effects of glass area, lighted window area, and proportion of glass lighted by monitoring buildings that vary independently in regard to these factors or by experimentally changing amounts of light emitted from buildings with different amounts of glass area and measuring collision rates with different treatments. Even in lieu of research clearly documenting causation, we argue there is sufficient circumstantial evidence regarding nighttime lighting effects on bird-building collisions to expand efforts to reduce light pollution in downtown areas and other settings.

We are uncertain why proportion lighted was associated with numbers of species colliding but not total collision fatalities, and with fatalities in spring but not fall. The former pattern could occur if lighting has the greatest effect during migration periods (e.g., particular times of the night or year) with a high diversity, but not necessarily the greatest number, of migrating birds. Lighting could disproportionately influence spring fatalities if this season has a higher frequency of weather conditions that exacerbate light pollution effects (e.g., low cloud ceilings; heavy precipitation) and/or if the mix of species migrating during spring is collectively more sensitive to light pollution. Further research into the mechanisms behind light pollution effects on migratory birds, including for different seasons and species, would help clarify the role of lighting in bird collisions.

## Conclusions

We illustrated substantial variation in bird collision rates in a major U.S. downtown area. A few large, glassy buildings with extensive surrounding vegetation—including a stadium and three high-rise office buildings—caused the majority of collisions and drove the importance of glass area and vegetation in explaining collision fatality rates. Excluding these buildings revealed slightly different collision correlates, although glass area and vegetation still predicted several collision variables. This result suggests that factors causing some buildings to cause exceptionally large numbers of collisions are not the exact same factors causing more modest collision variation among buildings that are more representative of most of those in downtown areas.

Our results suggest management approaches that can reduce bird collisions at both new and existing buildings. Reducing numbers of collisions and numbers of species colliding should be achievable by reducing light emission at night, reducing the area of untreated glass, and avoiding the use of vegetation near glassy surfaces. Mitigation strategies for existing buildings include treatments that provide visual markers and/or reduce reflective and see-through effects of glass (e.g., window film applications); such treatments are likely to be especially important for buildings that emit extensive lighting at night and are in close proximity to extensive vegetation and greenspaces. Collisions should also be reducible by considering such features in the design and construction of new buildings, including stadiums and the many other large and/or glassy structures that are otherwise likely to cause large numbers of bird collisions. Finally, further field-testing and peer-reviewed research is needed to provide rigorous validation of bird-friendly construction approaches and measures to reduce collisions at existing buildings. Such management and research regarding approaches to reduce bird-building collisions will be crucial for mitigating this major threat to bird populations.

## Supporting information

S1 Table Correlation matrix. Correlation matrix for all predictor variables assessed.
(DOCX)

S2 Table Total species collision counts. Total collision counts, including both fatal and non-fatal collisions, for all species observed as collision casualties.
(DOCX)

S3 Table Seasonal species collision counts. Collision counts by monitoring season, including both fatal and non-fatal collisions, for all species observed as collision casualties.
(DOCX)

S4 Table Supported variables (outliers excluded). Standardized coefficient estimates for variables in supported models for analyses excluding outlier buildings.
(DOCX)

S1 Fig Correlates of numbers of collision fatalities (outliers excluded). Relationships between low raw counts of collision fatalities and supported variables for analysis excluding outlier buildings.
(DOCX)

S2 Fig Correlates of numbers of species colliding (outliers excluded). Relationships between total numbers of species colliding and supported variables for analysis excluding outlier buildings.
(DOCX)

S1 Dataset Data used for GenEst fatality estimates. Input data for GenEst analysis to generate estimates of fatal collisions adjusted to account for removal of bird carcasses by humans and animal scavengers and for imperfect detection of carcasses present during surveys (metadata and analysis description in S1 Appendix); U.S. Bank Stadium is building 1.
(XLSX)

S2 Dataset Data used for analyses including all buildings. Input data for analyses of building-related variables associated with bird collisions (based on all 21 buildings); U.S. Bank Stadium is building 1.
(XLSX)

S3 Dataset Data used for analyses with outliers excluded. Input data for analyses of building-related variables associated with bird collisions (based on 17 buildings with 4 outliers excluded).
(XLSX)

S1 Appendix Metadata for S1 Dataset. Metadata for S1 Dataset used to estimate bias-adjusted fatality rates with GenEst, and additional documentation for GenEst analysis.
(DOCX)

S2 Appendix R code for analyses including all buildings. R code for analyses of building-related variables associated with bird collisions (based on all 21 buildings; data in S2 Dataset).
(DOCX)

S3 Appendix R code for analyses with outliers excluded. R code for analyses of building-related variables associated with bird collisions (based on 17 buildings with 4 outliers excluded; data in S3 Dataset).
(DOCX)
