# Supplementary material for: Factors influencing bird-building collisions in the downtown area of a major North American city
Source: PLoS One. 2019 Nov 6;14(11):e0224164. doi: 10.1371/journal.pone.0224164 (PMC6834121; doi:10.1371/journal.pone.0224164)
Supplement: S1 Table — Correlation matrix for all predictor variables assessed. (DOCX) [file pone.0224164.s001.docx]

**S1 Table.** **Correlation matrix.** Pearson’s correlation values for all pairs of predictor variables assessed in analyses of building-related factors associated with bird collisions based on monitoring at 21 buildings, including U.S. Bank Stadium, in downtown Minneapolis, Minnesota, USA, 2017-2018.

|  |  |  |  |  |  |  | Prop. vegetation^g^ | |
| --- | --- | --- | --- | --- | --- | --- | --- | --- |
|  | Height (m)^a^ | Glass area (m^2^)^b^ | Prop. light^c^ | Area light^d^ (m^2^) | Footprint (m^2^)^e^ | Distance to river (m)^f^ | 50 m buffer | 100 m buffer |
| Height (m) | 1.000 | - | - | - | - | - | - | - |
| Glass area (m^2^) | 0.752 | 1.000 | - | - | - | - | - | - |
| Prop. light | -0.087 | -0.041 | 1.000 | - | - | - | - | - |
| Area light | 0.357 | 0.698 | 0.474 | 1.000 | - | - | - | - |
| Footprint (m^2^) | 0.043 | 0.436 | 0.411 | 0.850 | 1.000 | - | - | - |
| Distance to river (m) | 0.172 | 0.013 | 0.170 | -0.043 | -0.118 | 1.000 | - | - |
| Prop. vegetation (50 m) | 0.209 | 0.310 | 0.193 | 0.596 | 0.426 | 0.012 | 1.000 | - |
| Prop. vegetation (100 m) | 0.193 | 0.332 | 0.059 | 0.546 | 0.444 | -0.164 | 0.797 | 1.000 |

^a^Estimated height of the main roof of the building

^b^Total estimated area of glass (including windows and other glass surfaces) on all building facades combined, excluding glass recessed from the main façade for which collision casualties were likely to land on elevated surfaces not covered by surveys

^c^Proportion of all glass surfaces emitting artificial light during nighttime periods (calculated by dividing Area light by Glass area)

^d^Area of all windows emitting artificial light during nighttime periods

^e^Horizontal ground area covered by the building (based on building’s outer edge)

^f^Distance from building centroid to nearest edge of the Mississippi River corridor

^g^Proportion of land covered by vegetation within 50 and 100m of building (includes grass/shrub and deciduous/coniferous trees; excludes bare soil, roads and other paved surfaces, and other buildings)
