# Supplementary material for: Factors influencing bird-building collisions in the downtown area of a major North American city
Source: PLoS One. 2019 Nov 6;14(11):e0224164. doi: 10.1371/journal.pone.0224164 (PMC6834121; doi:10.1371/journal.pone.0224164)
Supplement: S2 Table — Total collision counts, including both fatal and non-fatal collisions, for all species observed as collision casualties. (DOCX) [file pone.0224164.s002.docx]

**S2 Table. Total species collision counts.** Counts of all species observed as collision casualties, including both fatal and non-fatal collisions, across all collision surveys at all 21 buildings, including U.S. Bank Stadium, in downtown Minneapolis, Minnesota, USA, 2017-2018.

| Species | Count |  | Species | Count |  | Species | Count |
| --- | --- | --- | --- | --- | --- | --- | --- |
| White-throated Sparrow | 141 |  | Magnolia Warbler | 9 |  | American Tree Sparrow | 2 |
| Nashville Warbler | 108 |  | Gray Catbird | 7 |  | Blackpoll Warbler | 2 |
| Ovenbird | 98 |  | Canada Warbler | 7 |  | Fox Sparrow | 2 |
| Common Yellowthroat | 74 |  | Bay-breasted Warbler | 7 |  | Eastern Wood-pewee | 2 |
| Tennessee Warbler | 68 |  | Palm Warbler | 7 |  | Winter Wren | 2 |
| Dark-eyed Junco | 33 |  | Ruby-crowned Kinglet | 6 |  | Blackburnian Warbler | 2 |
| Unknown bird^a^ | 32 |  | Virginia Rail | 6 |  | Northern Parula | 2 |
| Black-and-white Warbler | 29 |  | Black-throated Green Warbler | 5 |  | American Coot | 2 |
| Ruby-throated Hummingbird | 26 |  | House Finch | 5 |  | Pied-billed Grebe | 1 |
| Northern Waterthrush | 22 |  | Hermit Thrush | 5 |  | Cape May Warbler | 1 |
| Lincoln's Sparrow | 21 |  | White-breasted Nuthatch | 5 |  | White-winged Crossbill | 1 |
| Red-breasted Nuthatch | 18 |  | Marsh Wren | 5 |  | Eastern Bluebird | 1 |
| Unknown warbler^b^ | 17 |  | Yellow-billed Cuckoo | 4 |  | Downy Woodpecker | 1 |
| House Sparrow | 15 |  | Wood Thrush | 4 |  | Red-necked Phalarope | 1 |
| Brown Creeper | 13 |  | Golden-winged Warbler | 4 |  | Cliff Swallow | 1 |
| Mourning Warbler | 13 |  | Northern Flicker | 4 |  | Sedge Wren | 1 |
| American Redstart | 12 |  | Song Sparrow | 4 |  | Wilson's Warbler | 1 |
| American Woodcock | 12 |  | Chipping Sparrow | 3 |  | Mourning Dove | 1 |
| Black-billed Cuckoo | 12 |  | Yellow Warbler | 3 |  | Common Grackle | 1 |
| Swamp Sparrow | 12 |  | American Robin | 3 |  | Belted Kingfisher | 1 |
| Unknown sparrow^c^ | 11 |  | Red-eyed Vireo | 3 |  | Yellow-bellied Flycatcher | 1 |
| Black-capped Chickadee | 10 |  | Unk. *Empidonax* flycatcher^d^ | 3 |  | Blue Jay | 1 |
| Chestnut-sided Warbler | 9 |  | House Wren | 3 |  | Baltimore Oriole | 1 |
| Orange-crowned Warbler | 9 |  | Sora | 3 |  | Blue-headed Vireo | 1 |
| Rock Dove | 9 |  | Indigo Bunting | 3 |  | Grasshopper Sparrow | 1 |
| Yellow-rumped Warbler | 9 |  | Swainson's Thrush | 3 |  | Unknown hummingbird^e^ | 1 |
| Yellow-bellied Sapsucker | 9 |  | Savannah Sparrow | 3 |  | TOTAL | 1000 |

^a^Birds that could not be identified to any taxonomic level, typically due to dismemberment, severe decomposition, distant viewing and/or poor quality documentation photos

^b^Warblers that could not be identified to species level, typically due to dismemberment, severe decomposition, distant viewing and/or poor quality documentation photos

^c^Sparrows that could not be identified to species level, typically due to dismemberment, severe decomposition, distant viewing and/or poor quality documentation photos

^d^Flycatchers in the genus *Empidonax* that could not be identified to species level, typically due to confusing plumage or inability to collect morphological measurements that facilitate species identification for this bird group

^e^Hummingbirds that could not be identified to species level due to confusing plumage or inability to collect morphological measurements that facilitate species identification for this bird group
