# Supplementary material for: Factors influencing bird-building collisions in the downtown area of a major North American city
Source: PLoS One. 2019 Nov 6;14(11):e0224164. doi: 10.1371/journal.pone.0224164 (PMC6834121; doi:10.1371/journal.pone.0224164)
Supplement: S3 Table — Collision counts by monitoring season, including both fatal and non-fatal collisions, for all species observed as collision casualties. (DOCX) [file pone.0224164.s003.docx]

**S3 Table. Seasonal species collision counts.** Counts of all species observed as collision casualties, including both fatal and non-fatal collisions, during spring, summer, and fall surveys at all 21 monitored buildings, including U.S. Bank Stadium, in downtown Minneapolis, Minnesota, USA, 2017-2018.

| Spring (15 Mar-31 May) | | Summer (1-30 Jun) | | Fall (15 Aug-31 Oct) | |
| --- | --- | --- | --- | --- | --- |
| Species | Count | Species | Count | Species | Count |
| Ovenbird | 37 | House Sparrow | 6 | White-throated Sparrow | 107 |
| White-throated Sparrow | 34 | Black-billed Cuckoo | 5 | Nashville Warbler | 104 |
| Tennessee Warbler | 15 | Yellow-billed Cuckoo | 4 | Common Yellowthroat | 66 |
| Unknown bird^a^ | 13 | House Finch | 4 | Ovenbird | 61 |
| American Woodcock | 8 | Common Yellowthroat | 3 | Tennessee Warbler | 53 |
| Black-billed Cuckoo | 7 | Unknown bird^a^ | 2 | Dark-eyed Junco | 26 |
| Northern Waterthrush | 7 | Chipping Sparrow | 1 | Ruby-throated Hummingbird | 23 |
| Dark-eyed Junco | 7 | Virginia Rail | 1 | Black-and-white Warbler | 23 |
| Black-and-white Warbler | 6 | Mourning Warbler | 1 | Lincoln's Sparrow | 19 |
| Yellow-bellied Sapsucker | 5 | Red-eyed Vireo | 1 | Red-breasted Nuthatch | 18 |
| Common Yellowthroat | 5 | Unknown sparrow^c^ | 1 | Unknown bird^a^ | 17 |
| House Sparrow | 5 | American Woodcock | 1 | Unknown warbler^b^ | 16 |
| Swamp Sparrow | 4 | Cliff Swallow | 1 | Northern Waterthrush | 15 |
| Northern Flicker | 4 | Indigo Bunting | 1 | Mourning Warbler | 12 |
| Nashville Warbler | 4 | Lincoln's Sparrow | 1 | Brown Creeper | 11 |
| Unknown sparrow^c^ | 3 | - | - | Black-capped Chickadee | 9 |
| American Redstart | 3 | - | - | Orange-crowned Warbler | 9 |
| Ruby-throated Hummingbird | 3 | - | - | Magnolia Warbler | 9 |
| Gray Catbird | 3 | - | - | Chestnut-sided Warbler | 9 |
| Brown Creeper | 2 | - | - | American Redstart | 9 |
| Hermit Thrush | 2 | - | - | Swamp Sparrow | 8 |
| White-breasted Nuthatch | 2 | - | - | Yellow-rumped Warbler | 8 |
| Swainson's Thrush | 2 | - | - | Rock Dove | 8 |
| Wood Thrush | 2 | - | - | Unknown sparrow^c^ | 7 |
| Indigo Bunting | 2 | - | - | Bay-breasted Warbler | 7 |
| Virginia Rail | 2 | - | - | Canada Warbler | 6 |
| Marsh Wren | 2 | - | - | Palm Warbler | 6 |
| Canada Warbler | 1 | - | - | Ruby-crowned Kinglet | 5 |
| Red-necked Phalarope | 1 | - | - | Black-throated Green Warbler | 5 |
| Downy Woodpecker | 1 | - | - | Gray Catbird | 4 |
| Eastern Wood-pewee | 1 | - | - | Yellow-bellied Sapsucker | 4 |
| Ruby-crowned Kinglet | 1 | - | - | Song Sparrow | 4 |
| Unk. *Empidonax* flycatcher^d^ | 1 | - | - | House Sparrow | 4 |
| Unknown warbler^b^ | 1 | - | - | American Robin | 3 |
| Golden-winged Warbler | 1 | - | - | White-breasted Nuthatch | 3 |
| Yellow-rumped Warbler | 1 | - | - | Virginia Rail | 3 |
| Grasshopper Sparrow | 1 | - | - | Savannah Sparrow | 3 |
| Rock Dove | 1 | - | - | Hermit Thrush | 3 |
| House Wren | 1 | - | - | Golden-winged Warbler | 3 |
| Baltimore Oriole | 1 | - | - | American Woodcock | 3 |
| Black-capped Chickadee | 1 | - | - | Sora | 3 |
| Chipping Sparrow | 1 | - | - | Marsh Wren | 3 |
| Lincoln's Sparrow | 1 | - | - | Winter Wren | 2 |
| Yellow Warbler | 1 | - | - | Unk. *Empidonax* flycatcher^d^ | 2 |
| Palm Warbler | 1 | - | - | Yellow Warbler | 2 |
| Red-eyed Vireo | 1 | - | - | Blackburnian Warbler | 2 |
| Eastern Bluebird | 1 | - | - | House Wren | 2 |
| - | - | - | - | Northern Parula | 2 |
| - | - | - | - | Wood Thrush | 2 |
| - | - | - | - | American Tree Sparrow | 2 |
| - | - | - | - | American Coot | 2 |
| - | - | - | - | Blackpoll Warbler | 2 |
| - | - | - | - | Fox Sparrow | 2 |
| - | - | - | - | Yellow-bellied Flycatcher | 1 |
| - | - | - | - | White-winged Crossbill | 1 |
| - | - | - | - | Unknown hummingbird^e^ | 1 |
| - | - | - | - | Eastern Wood-pewee | 1 |
| - | - | - | - | Common Grackle | 1 |
| - | - | - | - | Chipping Sparrow | 1 |
| - | - | - | - | Red-eyed Vireo | 1 |
| - | - | - | - | Sedge Wren | 1 |
| - | - | - | - | House Finch | 1 |
| - | - | - | - | Mourning Dove | 1 |
| - | - | - | - | Wilson's Warbler | 1 |
| - | - | - | - | Belted Kingfisher | 1 |
| - | - | - | - | Pied-billed Grebe | 1 |
| - | - | - | - | Swainson's Thrush | 1 |
| - | - | - | - | Cape May Warbler | 1 |
| - | - | - | - | Blue Jay | 1 |
| - | - | - | - | Blue-headed Vireo | 1 |
|  | **209** |  | **33** |  | **758** |

^a^Birds that could not be identified to any taxonomic level, typically due to dismemberment, severe decomposition, distant viewing and/or poor quality documentation photos

^b^Warblers that could not be identified to species level, typically due to dismemberment, severe decomposition, distant viewing and/or poor quality documentation photos

^c^Sparrows that could not be identified to species level, typically due to dismemberment, severe decomposition, distant viewing and/or poor quality documentation photos

^d^Flycatchers in the genus *Empidonax* that could not be identified to species level, typically due to confusing plumage or inability to collect morphological measurements that facilitate species identification for this bird group

^e^Hummingbirds that could not be identified to species level due to confusing plumage or inability to collect morphological measurements that facilitate species identification for this bird group
