# Supplementary material for: Factors influencing bird-building collisions in the downtown area of a major North American city
Source: PLoS One. 2019 Nov 6;14(11):e0224164. doi: 10.1371/journal.pone.0224164 (PMC6834121; doi:10.1371/journal.pone.0224164)
Supplement: S4 Table — Standardized coefficient estimates for variables in supported models for analyses excluding outlier buildings. (DOCX) [file pone.0224164.s004.docx]

**S4 Table. Supported variables (outliers excluded).** Standardized coefficient estimates for variables included in strongly supported models for analyses of building-related variables associated with bird collisions based on monitoring at a subset of 17 of 21 buildings (i.e., with exclusion of potential outliers: stadium, #3, #4, and #17) monitored in downtown Minneapolis, Minnesota, USA, 2017-2018. Analyses were conducted for total collision fatalities across all seasons and for spring and fall, for total collision fatalities for the five species most frequently observed as collision casualties, and for numbers of species colliding across all seasons and for spring and fall. For results based on all 21 buildings monitored, see Table 4.

|  |  |  |  |  |  |  | Prop. vegetation | |
| --- | --- | --- | --- | --- | --- | --- | --- | --- |
|  | Height | Glass area | Prop. light | Area light | Footprint | Distance to river | 50 m buffer | 100 m buffer |
| *Collision fatalities (all)* |  |  |  |  |  |  |  |  |
| Total low raw count^a^ | - | 0.042 | - | - | - | - | - | 0.026 |
| Total high adj.estimate^b^ | - | - | - | - | - | - | - | - |
| Spring low raw count^c^ | - | - | 0.299 | - | - | - | 0.213 | 0.249 |
| Fall low raw count^d^ | - | 0.068 | - | - | - | - | - | 0.056 |
| *Collision fatalities (species)^e^* |  |  |  |  |  |  |  |  |
| White-throated Sparrow | - | 0.484 | - | - | - | - | - | - |
| Nashville Warbler | 0.830 | - | - | - | 0.587 | - | 0.977 | - |
| Ovenbird | - | 0.446 | - | - | - | - | - | - |
| Common Yellowthroat | - | 1.713 | - | - | - | - | 0.981 | 1.501 |
| Tennessee Warbler | - | - | - | - | - | - | - | - |
| *Number of species^f^* |  |  |  |  |  |  |  |  |
| All seasons | - | 0.073 | 0.075 | - | - | - | - | 0.070 |
| Spring | - | - | 0.198 | - | - | - | - | 0.252 |
| Fall | - | 0.102 | - | - | - | - | - | 0.087 |

^a^Analysis response variable was raw counts of total fatal collision casualties excluding birds potentially resulting from predation events and collisions with skyways connecting buildings

^b^Analysis response variable was bias-adjusted estimates of fatal collisions adjusted to account for removal of bird carcasses by humans and animal scavengers and for imperfect detection of carcasses present during surveys (this version of the bias-adjusted estimate was based on the high raw count of fatal collisions, which included birds potentially resulting from predation events and collisions with skyways connecting buildings); results not shown because statistical model did not converge (see Results in main text).

^c^Analysis response variable was raw counts of spring fatal collision casualties excluding birds potentially resulting from predation events and collisions with skyways connecting buildings

^d^Analysis response variable was raw counts of fall fatal collision casualties excluding birds potentially resulting from predation events and collisions with skyways connecting buildings

^e^Analysis response variables were low raw counts of fatal collision casualties for individual species, excluding birds potentially resulting from predation events and collisions with skyways connecting buildings

^f^Analysis response variables were total numbers of identifiable species observed as fatal and non-fatal collision casualties at each building
