# Supplementary material for: Factors influencing bird-building collisions in the downtown area of a major North American city
Source: PLoS One. 2019 Nov 6;14(11):e0224164. doi: 10.1371/journal.pone.0224164 (PMC6834121; doi:10.1371/journal.pone.0224164)
Supplement: S1 Fig — Relationships between low raw counts of collision fatalities and supported variables for analysis excluding outlier buildings. (DOCX) [file pone.0224164.s005.docx]

**S1 Fig. Correlates of numbers of collision fatalities (outliers excluded)**. Relationships between low raw counts of collision fatalities^a^ and (a) glass area, and (b) proportion of land covered by vegetation within 100 m; for analysis excluding outlier buildings (stadium, #3, #4, and #17). For results based on all 21 monitored buildings, see text and Fig 3.


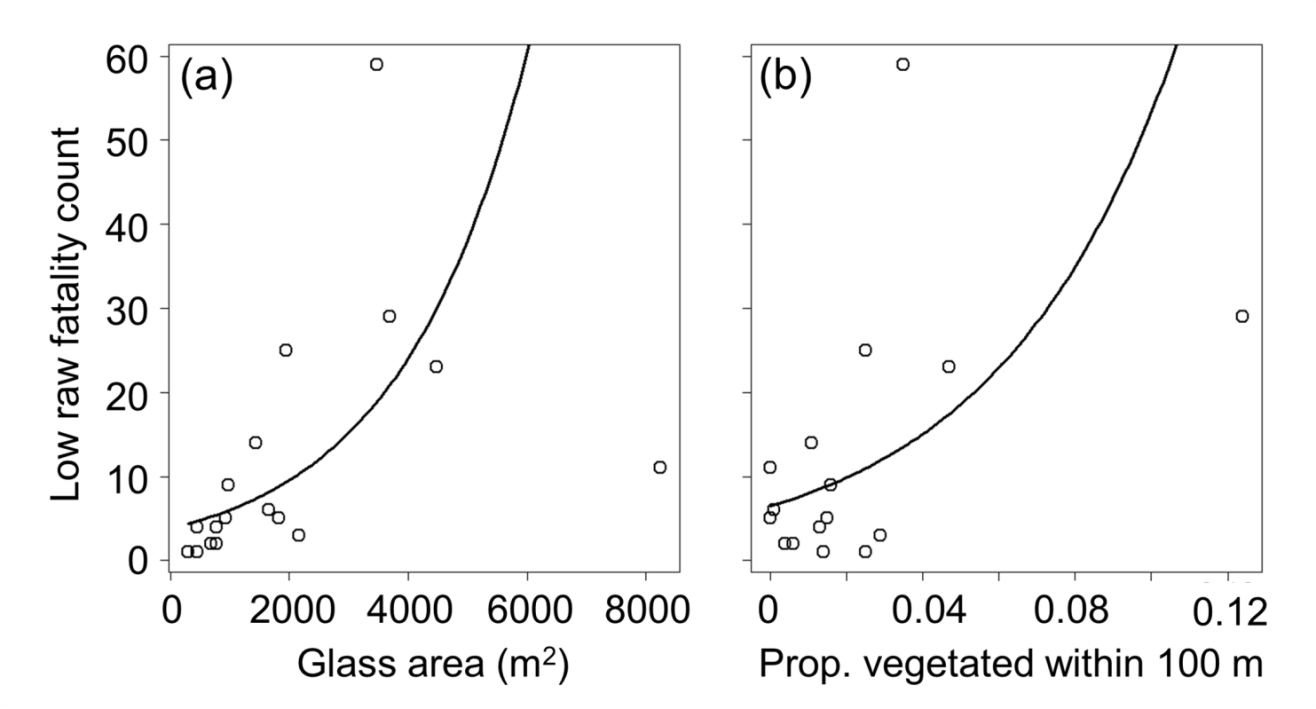


^a^Analysis response variable was raw counts of total fatal collision casualties excluding birds potentially resulting from predation events and collisions with skyways connecting buildings. Although the complementary analysis including all 21 buildings (Fig. 3) was based on adjusted fatality estimates (i.e., estimates that account for removal of bird carcasses by humans and animal scavengers, and for imperfection detection of carcasses present during surveys), figures are based on raw counts because the model using bias-adjusted estimates did not converge with the four potential outlier buildings removed.
