# Supplementary material for: Factors influencing bird-building collisions in the downtown area of a major North American city
Source: PLoS One. 2019 Nov 6;14(11):e0224164. doi: 10.1371/journal.pone.0224164 (PMC6834121; doi:10.1371/journal.pone.0224164)
Supplement: S2 Fig — Relationships between total numbers of species colliding and supported variables for analysis excluding outlier buildings. (DOCX) [file pone.0224164.s006.docx]

**S2 Fig. Correlates of numbers of species colliding (outliers excluded).** Based on a subset of 17 of 21 buildings (four potential high outliers removed, including stadium, #3, #4, and #17) monitored in downtown Minneapolis, MN, USA, 2017-2018, relationships between total numbers of species observed as casualties (including both fatal and non-fatal collisions) and (a) glass area, (b) proportion of glass area with lighting emitted at night, and (c) proportion of land covered by vegetation within 100 m. For results based on all 21 monitored buildings, see text and Figure 4.

**
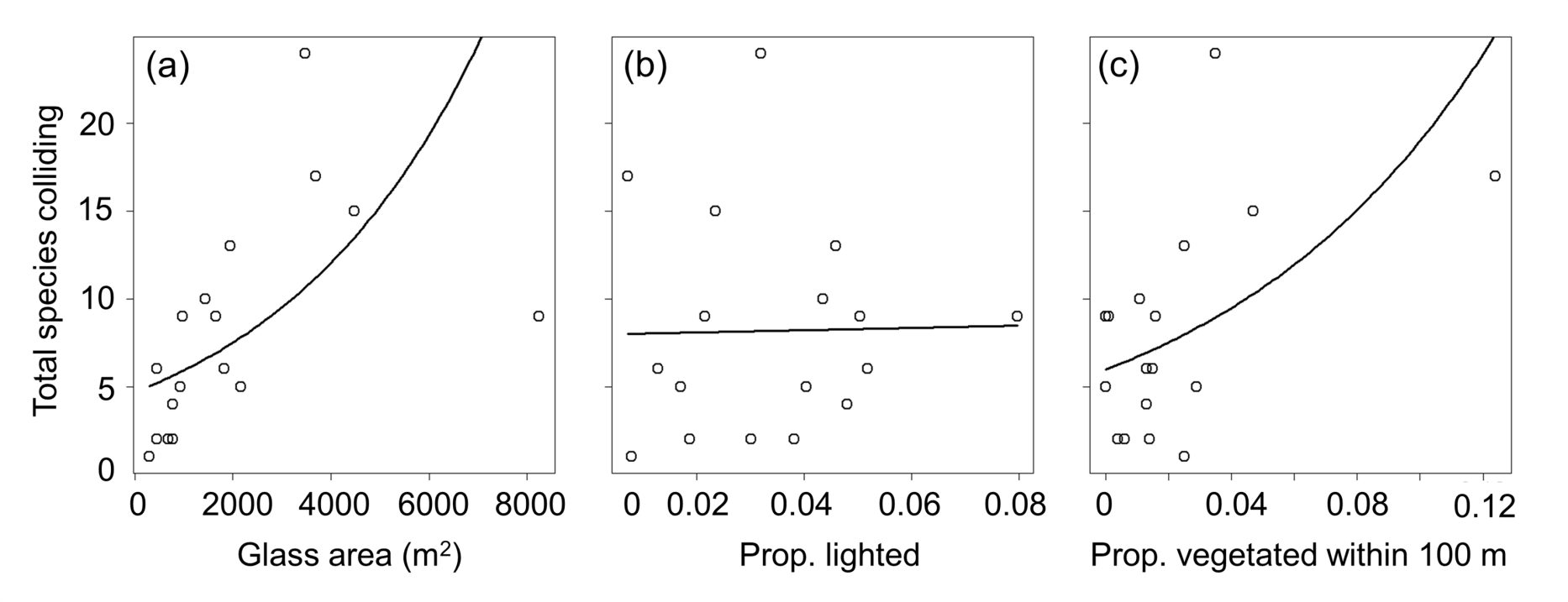
**
