# Supplementary material for: Factors influencing bird-building collisions in the downtown area of a major North American city
Source: PLoS One. 2019 Nov 6;14(11):e0224164. doi: 10.1371/journal.pone.0224164 (PMC6834121; doi:10.1371/journal.pone.0224164)
Supplement: S1 Appendix — Metadata for S1 Dataset used to estimate bias-adjusted fatality rates with GenEst, and additional documentation for GenEst analysis. (DOCX) [file pone.0224164.s010.docx]

**S1 Appendix. Metadata for S1 Dataset.** Metadata for S1 Dataset used to estimate bias-adjusted fatality rates using GenEst fatality estimator, and additional documentation for GenEst analysis.

**S1 Dataset is one Microsoft Excel workbook including the following five worksheets:**

CarcassObservations

DWP

GenEst_CarcassPersistence

GenEst_SearchEfficiency

GenEst_SearchSchedule

**Description:**

The five worksheets contain data used to estimate bias-adjusted fatality estimates, which account for animal and human removal of carcasses between surveys and imperfect detection of carcasses present on surveys.

- Worksheets starting with ‘GenEst’ contain data uploaded directly to GenEst package GUI in R (Dalthorp et al. 2018).
  - GenEst_CarcassPersistence contains data for removal trials used to estimate carcass persistence.
  - GenEst_SearchEfficiency contains data for searcher detection trials used to estimate searcher efficiency.
  - GenEst_SearchSchedule contains data to account for varying number of surveys at each building; used with GenEst_CarcassPersistence and GenEst_SearchEfficiency to estimate detection probability at each building.
- CarcassObservations contains data with raw (unadjusted) fatality counts.
- DWP contains density-weighted proportion data for approximate percentage of carcasses expected to occur at different substrate types at each building (estimated using scavenger removal data and proportion of surface types around the perimeter of each building)

**Column definitions:**

CarcassObservations worksheet:

- **BuildingID:** Unique identifier for each building surveyed.
- **LowCO:** Low raw count of carcasses (excludes carcasses identified as possible skyway collisions and parts of carcasses that may have resulted from predation events instead of collisions).
- **HighCO:** High raw count of carcasses (includes all observed carcasses).

DWP worksheet:

- **BuildingID:** Unique identifier for each building surveyed.
- **Artificial:** Approximate proportion of artificial substrates (e.g., concrete, metal) at given building.
- **Natural:** Approximate proportion of “natural” substrates (e.g., planters, dirt) at given building.
- **Rocks:** Approximate proportion of rocky substrates at given building.

GenEst_CarcassPersistence worksheet:

- **SRT_ID:** Unique identifier for each dead bird used for scavenger removal trials.
- **LastPresent**: Number of days since placement that carcass was observed to be present. A numeric value from 0 to the value of FirstAbsent.
- **FirstAbsent**: Number of days since placement that carcass was observed to be missing.

A numeric value from the value of LastPresent to Inf, where Inf represents carcasses that remained present for at least 7 days, after which surveyors collected the carcass.

- **BuildingID**: Unique identifier for each building surveyed.
- **Substrate:** Material on which carcass was placed.
  - Artificial: concrete, metal, or building surfaces (e.g., granite)
  - Natural: planters, dirt, grass
  - Rocks: rock beds
- **Season:** Monitoring season during which trial occurred
  - Spring: 15 Mar – 31 May
  - Summer: 1 Jun – 30 Jun
  - Fall: 15 Aug – 31 Oct

GenEst_SearchEfficiency worksheet:

- **SD_ID:** Unique identifier for carcasses placed for searcher detection trials.
- **BuildingID:** Unique identifier for each building surveyed.
- **Substrate:** Material on which carcass was placed.
  - Artificial: concrete, metal, or building surfaces (e.g., granite)
  - Natural: planters, dirt, grass
  - Rocks: rock beds
- **Surveyor**: Unique identifier for surveyor tested.
- **Detection**: Indicates whether carcass was detected.
  - 0: carcass not detected
  - 1: carcass detected
  - NA: carcass not detected but absent at time of retrieval (assumed to have been removed before surveyor had the opportunity to detect the carcass)
- **Season:** Monitoring season during which trial occurred
  - Spring: 15 Mar – 31 May
  - Summer: 1 Jun – 30 Jun
  - Fall: 15 Aug – 31 Oct

GenEst_SearchSchedule worksheet:

- SearchDate: Date of survey.
- Columns 2-22 represent different buildings surveyed. Row values are either 1 or 0, indicating whether the building was searched (1) or not (0) on the given date.

**GenEst** **parameters and notes:**

- Required general inputs:
  - Number of iterations: 1000
  - Confidence level: 0.95
- Searcher efficiency *k* parameter fixed to 0.9 (𝑘 represents proportional change in searcher efficiency with each successive search, where 0 indicates that carcasses missed on the first search cannot be found in a later search, and 1 indicates constant search efficiency regardless of carcass age). We did not have GenEst estimate *k* based on our search data because we did not typically leave carcasses in place for subsequent searches.
- We did not use GenEst to calculate final fatality estimates because we lacked substrate data for carcass observations. We instead estimated fatalities for each building by:
  1. Multiplying detection probability by the density-weighted proportion of each substrate
  2. Adding the result for each substrate to obtain a weighted detection probability
  3. Dividing carcass observations by the weighted detection probability

**Literature cited:**

Dalthorp D, Madsen L, Huso M, Rabie P, Wolpert R, Studyvin J, et al. 2018. GenEst statistical models—A generalized estimator of mortality: U.S. Geological Survey Techniques and Methods, book 7, chap. A2, 13 p., https://doi.org/10.3133/tm7A2
