# Supplementary material for: Factors influencing bird-building collisions in the downtown area of a major North American city
Source: PLoS One. 2019 Nov 6;14(11):e0224164. doi: 10.1371/journal.pone.0224164 (PMC6834121; doi:10.1371/journal.pone.0224164)
Supplement: S2 Appendix — R code for analyses of building-related variables associated with bird collisions (based on all 21 buildings; data in S2 Dataset). (DOCX) [file pone.0224164.s011.docx]

**S2 Appendix**. **R code for analyses including all buildings.** R code for analyses of building-related variables associated with bird collisions based on monitoring at 21 buildings in downtown Minneapolis, Minnesota, USA. Data used for these analyses are in S2 Dataset.

###Analyses below require the following packages to be installed and loaded:

#lme4

#MASS

#pscl

#AICcmodavg

#QuantPsyc(to generate standardized coefficients)

###Load data

BuildingCompareALL <- read.table("MN_Building_Collision­­Analysis_ALL_BUILDINGS.txt", header = TRUE) #Loads data to R data frame

attach(BuildingCompareALL) #Attaches data to R data frame so do not need to reference data object name in remainder of analysis

str(BuildingCompareALL) #Displays data

###KEY for variable names referenced in this file:

#BuildingNumber - Building ID code assigned for study purposes (US Bank Stadium = 1)

#Quintile - The quintile each building was originally placed into based on total number of collisions observed for Project Birdsafe (2007-2015); this information was used to select 16 study buildings from a larger set of 64 buildings monitored for Project BirdSafe (NA indicates newly selected buildings with no past monitoring history)

#HeightFt - Estimated height of main roof (in feet)

#HeightM - Estimated height of main roof (in meters)

#GlassAreaSqFt - Total estimated area of glass on all building facades (square ft)

#GlassAreaSqM - Total estimated area of glass on all building facades (square m)

#AreaLight - Area of lighted windows across entire building (square m)

#PropLit - Proportion of glass surfaces lit across entire building (calculated by dividing AreaLight by GlassArea)

#Footprint - Horizontal ground area covered by the building (square m); calculated using the building polygon file and ArcGIS's Calculate Geometry option in the shape file's attribute table

#DistanceRiver - Distance (m) from building centroid to nearest edge of Mississippi River corridor (calculated using Euclidian Distance Tool in GIS's Spatial Analyst Tools Directory)

#Vegetation50 - Proportion of vegetated land within 50 m of building edge (includes grass/shrub, deciduous tree cover, and coniferous tree cover; excludes buildings, roads/paved surfaces, and bare soil)

#Vegetation100 - Proportion of vegetated land within 100 m of building edge (includes grass/shrub, deciduous tree cover, and coniferous tree cover; excludes buildings, roads/paved surfaces, and bare soil)

#LowRawFatal2017 - 2017 raw counts of fatal collisions (count is considered low because it excludes birds that possibly collided with skyways connecting buildings, not buildings themselves, and potential predation events, not collisions)

#LowRawFatal2018 - 2018 raw counts of fatal collisions (count is considered low because it excludes birds that possibly collided with skyways connecting buildings, not buildings themselves, and potential predation events, not collisions)

#LowRawAll2017 - 2017 raw counts of all fatal and non-fatal collisions (count is considered low because it excludes birds that possibly collided with skyways connecting buildings, not buildings themselves, and potential predation events, not collisions)

#LowRawAll2018 - 2018 raw counts of all fatal and non-fatal collisions (count is considered low because it excludes birds that possibly collided with skyways connecting buildings, not buildings themselves, and potential predation events, not collisions)

#LowRawNonFatal - Total raw counts of non-fatal collision sacross both 2017 and 2018 (count is considered low because it excludes birds that possibly collided with skyways connecting buildings, not buildings themselves, and potential predation events, not collisions)

#HighRawNonFatal - Total raw counts of non-fatal collisions across both 2017 and 2018 (count is considered high because it includes birds that possibly collided with skyways connecting buildings, not buildings themselves, and potential predation events, not collisions)

#LowRawFatal - Total raw count of fatal collisions across both 2017 and 2018 (count is considered low because it excludes birds that possibly collided with skyways connecting buildings, not buildings themselves, and potential predation events, not collisions)

#HighRawFatal - Total raw count of fatal collisions across both 2017 and 2018 (count is considered high because it includes birds that possibly collided with skyways connecting buildings, not buildings themselves, and potential predation events, not collisions)

#LowAdjustedFatal - Median estimate of bias-adjusted fatalities (i.e., accounting for searcher detection rate and human and animal removal of carcasses) across both 2017 and 2018 based on low raw count of fatal collisions

#HighAdjustedFatal - Median estimate of bias-adjusted fatalities (i.e., accounting for searcher detection rate and human and animal removal of carcasses) across both 2017 and 2018 based on high raw count of fatal collisions

#SpringLowRawFatal - Low raw count of all spring fatal collisions across both 2017 and 2018 (count is considered low because it excludes birds that possibly collided with skyways connecting buildings, not buildings themselves, and potential predation events, not collisions)

#FallLowRawFatal - Low raw count of all fall fatal collisions across both 2017 and 2018 (count is considered low because it excludes birds that possibly collided with skyways connecting buildings, not buildings themselves, and potential predation events, not collisions)

#WTSPLowRawFatal - Low raw count of all White-throated Sparrow fatal collisions across both 2017 and 2018 (count is considered low because it excludes birds that possibly collided with skyways connecting buildings, not buildings themselves, and potential predation events, not collisions)

#NAWALowRawFatal - Low raw count of all Nashville Warbler fatal collisions across both 2017 and 2018 (count is considered low because it excludes birds that possibly collided with skyways connecting buildings, not buildings themselves, and potential predation events, not collisions)

#OVENLowRawFatal - Low raw count of all Ovenbird fatal collisions across both 2017 and 2018 (count is considered low because it excludes birds that possibly collided with skyways connecting buildings, not buildings themselves, and potential predation events, not collisions)

#COYELowRawFatal - Low raw count of all Common Yellowthroat fatal collisions across both 2017 and 2018 (count is considered low because it excludes birds that possibly collided with skyways connecting buildings, not buildings themselves, and potential predation events, not collisions)

#TEWALowRawFatal - Low raw count of all Tennessee Warbler fatal collisions across both 2017 and 2018 (count is considered low because it excludes birds that possibly collided with skyways connecting buildings, not buildings themselves, and potential predation events, not collisions)

#SpeciesFatal - Total number of species observed as fatal collisions across across both 2017 and 2018

#SpeciesNonFatal - Total number of species observed as non-fatal collisions across across both 2017 and 2018

#SpeciesAll - Total number of species observed as fatal and non-fatal collisions across across both 2017 and 2018

#SpeciesAllSpring - Total number of species observed as spring fatal collisions during both 2017 and 2018

#SpeciesAllFall - Total number of species observed as fall fatal collisions during both 2017 and 2018

###Correlation tests for various bird collision response variables

cor(LowRawFatal, LowRawNonFatal) #r = 0.90

cor(HighRawFatal, HighRawNonFatal)#r = 0.89

cor(LowRawFatal, HighRawFatal) #r = 0.99

cor(LowRawFatal, HighAdjustedFatal) #r = 0.85

cor(LowRawFatal, LowAdjustedFatal) #0.94

###T-test to determine if collisions differ between 2017 and 2018

t.test(LowRawFatal2017, LowRawFatal2018, alternative = "two.sided", paired = TRUE) # t=-1.86, df = 20, p = 0.08

t.test(LowRawAll2017, LowRawAll2018, alternative = "two.sided", paired = TRUE) # t=-1.70 df = 20, p = 0.11

##NO DIFFERENCE IN FATAL COLLISIONS OR ALL COLLISIONS BETWEEN YEARS, SO FATALITIES FOR BOTH YEARS INCLUDED AS RESPONSE VARIABLE

###Conduct correlation analyses to identify strongly correlated variable pairs for later model selection analyses

AllPredictors <- BuildingCompareALL[,c(4, 6, 7, 8, 9, 10, 11, 12)] ###Makes data frame with all 8 predictor variables (HeightM, GlassAreaSqM, AreaLight, PropLit, Footprint, DistanceRiver, Vegetation50, Vegetation100)

head(AllPredictors,6) #Print 1st 6 rows to check new data frame

cor(AllPredictors) #Runs 8x8 correlation matrix using all of the predictors

###HeightM and GlassAreaSqM strongly correlated (r=0.75)

###GlassAreaSqM and AreaLight nearly stronglycorrelated (0.698)

###AreaLight and Footprint strongly correlated (0.85)

###Vegetation50 and Vegetation100 strongly correlated (r=0.80)

##############################################################################

###(1) Model selection analysis (Low Raw Count of TOTAL Fatal Collisions as Dep. Variable)

##############################################################################

###(A) Determine whether to use Poisson or Negative binomial statistical distribution based on null model and likelihood ratio test(Requires lme4, pscl, and MASS packages);Description of methods for running Likelihood ratio tests and calculating Chi-Square statistics/probabilities is at: http://stats.stackexchange.com/questions/127505/compare-poisson-and-negative-binomial-regression-with-lr-test

LowRawFatal_POISSON <- glm(LowRawFatal ~ 1, family = "poisson") ##Runs Poisson model on the null model (Requires lme4 package)

LowRawFatal_NEGBIN <- glm.nb(LowRawFatal ~ 1) ##Runs Negative Binomial model on the null model (requires MASS package)

###Likelihood ratio test to determine if Negative Binomial fits better than Poisson

2 * (logLik(LowRawFatal_NEGBIN) - logLik(LowRawFatal_POISSON)) ##Returns Chi-square statistic for comparison of two models

pchisq(2 * (logLik(LowRawFatal_NEGBIN) - logLik(LowRawFatal_POISSON)), df = 1, lower.tail = FALSE) ##Returns probability of chi-square statistic for comparison of two models

#Negative binomial supported

###(B) For strongly correlated predictor variable pairs, identify which more strongly associated with response variable

###HeightM and GlassAreaSqM

cor(HeightM, LowRawFatal) ## r = 0.43

cor(GlassAreaSqM, LowRawFatal) ## r = 0.60

##Retain GlassAreaSqM (exclude HeightM)

###GlassAreaSqM and AreaLight

cor(GlassAreaSqM, LowRawFatal) ## r = 0.60

cor(AreaLight, LowRawFatal) ## r = 0.80

##Retain both GlassAreaSqm and AreaLight because of frequent inclusion of window area in past studies just barely uncorrelated with area lit (r=0.698) depending on rounding

###AreaLight and Footprint

cor(AreaLight, LowRawFatal) ## r = 0.80

cor(Footprint, LowRawFatal) ## r = 0.45

##Retain AreaLight exclude Footprint)

###Vegetation50 and Vegetation100

cor(Vegetation50, LowRawFatal) ## r = 0.76

cor(Vegetation100, LowRawFatal) ## r = 0.73

##Retain Vegetation50 (exclude Vegetation100)

###(C) Conduct model selection exercise using remaining non-correlated predictor variables

###NULL MODEL

LowRawFatal_NULL<- glm.nb(LowRawFatal ~ 1)

###GLOBAL MODEL (Additive terms only)

LowRawFatal_GLOBAL <- glm.nb(LowRawFatal ~ GlassAreaSqM + PropLit + AreaLight + DistanceRiver + Vegetation50) ####Excludes HeightM, Footprint, Vegetation100 for strong correlations

###RUN STEPWISE BACKWARD AIC FUNCTION (Requires MASS Package)

LowRawFatalSTEP <- stepAIC(LowRawFatal_GLOBAL, scope = list(lower = ~1), Trace = FALSE)

LowRawFatalSTEP

###Results in 2 variables remaining (GlassAreaSqM and Vegetation50)

###Inspect Glass Area + Vegetation50 Model

LowRawFatal_GLASS_VEG50 <- glm.nb(LowRawFatal ~ GlassAreaSqM + Vegetation50)

LowRawFatal_GLASS_VEG50

summary(LowRawFatal_GLASS_VEG50) ##Generates coefficient estimates along with Standard errors

confint(LowRawFatal_GLASS_VEG50) ##Generates 95% confidence intervals of coefficient estimates

lm.beta(LowRawFatal_GLASS_VEG50) ##Generatess standardized coefficient values (requires QuantPsyc package)

###(D)Plot effects of supported variables

par(mfrow = c(1,1), mai = c(0.5, 0.6, 0.5, 0.6))

LowRawFatal_GLASS <- glm.nb(LowRawFatal ~ GlassAreaSqM)

plot(GlassAreaSqM, LowRawFatal, xlab = "", ylab = "", col = "black", cex = 2, cex.axis = 2.0, lwd = 2.5)

curve(predict(LowRawFatal_GLASS, data.frame(GlassAreaSqM=x), type="resp"),add=TRUE, col="black", lwd = 3)

LowRawFatal_VEG50 <- glm.nb(LowRawFatal ~ Vegetation50)

plot(Vegetation50, LowRawFatal, xlab = "", ylab = "", col = "black", cex = 2, cex.axis = 2.0, lwd = 2.5)

curve(predict(LowRawFatal_VEG50, data.frame(Vegetation50=x), type="resp"),add=TRUE, col="black", lwd = 3)

#################################################################################(2) Model selection analysis (High Median Bias-adjusted Estimates as Dependent Variable)

##############################################################################

###(A) Determine whether to use Poisson or Negative binomial statistical distribution based on null model and likelihood ratio test(Requires lme4, pscl, and MASS packages);Description of methods for running Likelihood ratio tests and calculating Chi-Square statistics/probabilities is at: http://stats.stackexchange.com/questions/127505/compare-poisson-and-negative-binomial-regression-with-lr-test

HighAdjustedFatal_POISSON <- glm(HighAdjustedFatal ~ 1, family = "poisson") ##Runs Poisson model on the null model (Requires lme4 package)

HighAdjustedFatal_NEGBIN <- glm.nb(HighAdjustedFatal ~ 1) ##Runs Negative Binomial model on the null model (requires MASS package)

##Likelihood ratio test to determine if Negative Binomial fits better than Poisson

2 * (logLik(HighAdjustedFatal_NEGBIN) - logLik(HighAdjustedFatal_POISSON)) ##Returns Chi-square statistic for comparison of two models

pchisq(2 * (logLik(HighAdjustedFatal_NEGBIN) - logLik(HighAdjustedFatal_POISSON)), df = 1, lower.tail = FALSE) ##Returns probability of chi-square statistic for comparison of two models

#Negative binomial supported

###(B) For strongly correlated predictor variable pairs, identify which more strongly associated with response variable

###HeightM and GlassAreaSqM

cor(HeightM, HighAdjustedFatal) ## r = 0.62

cor(GlassAreaSqM, HighAdjustedFatal) ## r = 0.69

##Retain GlassAreaSqM (exclude HeightM)

###GlassAreaSqM and AreaLight

cor(GlassAreaSqM, HighAdjustedFatal) ## r = 0.69

cor(AreaLight, HighAdjustedFatal) ## r = 0.623

##Retain both GlassAreaSqm and AreaLight because of frequent inclusion of window area in past studies just barely uncorrelated with area lit (r=0.698) depending on rounding

###AreaLight and Footprint

cor(AreaLight, HighAdjustedFatal) ## r = 0.63

cor(Footprint, HighAdjustedFatal) ## r = 0.24

##Retain AreaLight (exclude Footprint)

###Vegetation50 and Vegetation100

cor(Vegetation50, HighAdjustedFatal) ## r = 0.61

cor(Vegetation100, HighAdjustedFatal) ## r = 0.56

##Retain Vegetation50 (exclude Vegetation100)

###(C) Conduct model selection exercise using remaining non-correlated predictor variables

###NULL MODEL

HighAdjustedFatal_NULL<- glm.nb(HighAdjustedFatal ~ 1)

###GLOBAL MODEL (Additive terms only)

HighAdjustedFatal_GLOBAL <- glm.nb(HighAdjustedFatal ~ GlassAreaSqM + PropLit + AreaLight + DistanceRiver + Vegetation50) ###Excludes HeightM, Footprint, Vegetation100 for strong correlations

###RUN STEPWISE BACKWARD AIC FUNCTION (Requires MASS Package)

HighAdjustedFatalSTEP <- stepAIC(HighAdjustedFatal_GLOBAL, scope = list(lower = ~1), Trace = FALSE)

HighAdjustedFatalSTEP

###Results in 3 variables remaining (PropLit, GlassAreaSqM, and Vegetation50)

###Inspect Glass Area + PropLit + Vegetation50 Model

HighAdjustedFatal_GLASS_PROPLit_VEG50 <- glm.nb(HighAdjustedFatal ~ GlassAreaSqM + PropLit + Vegetation50)

HighAdjustedFatal_GLASS_PROPLit_VEG50

summary(HighAdjustedFatal_GLASS_PROPLit_VEG50) ##Generates coefficient estimates along with Standard errors

confint(HighAdjustedFatal_GLASS_PROPLit_VEG50) ##Generates 95% confidence intervals of coefficient estimates

lm.beta(HighAdjustedFatal_GLASS_PROPLit_VEG50) ##Generatess standardized coefficient values (requires QuantPsyc package)

##Above model indicates 95% CI of AreaLight overlaps zero so do not further include it

###(D)Plot effects of supported variables

par(mfrow = c(1,1), mai = c(0.5, 0.6, 0.5, 0.6))

HighAdjustedFatal_GLASS <- glm.nb(HighAdjustedFatal ~ GlassAreaSqM)

plot(GlassAreaSqM, HighAdjustedFatal, xlab = "", ylab = "", col = "black", cex = 2, cex.axis = 2.0, lwd = 2.5)

curve(predict(HighAdjustedFatal_GLASS, data.frame(GlassAreaSqM=x), type="resp"),add=TRUE, col="black", lwd = 3)

HighAdjustedFatal_VEG50 <- glm.nb(HighAdjustedFatal ~ Vegetation50)

plot(Vegetation50, HighAdjustedFatal, xlab = "", ylab = "", col = "black", cex = 2, cex.axis = 2.0, lwd = 2.5)

curve(predict(HighAdjustedFatal_VEG50, data.frame(Vegetation50=x), type="resp"),add=TRUE, col="black", lwd = 3)

##############################################################################

###(3) Model selection analysis (Low Raw Count of SPRING Fatal Collisions as Depend. Var.)

##############################################################################

###(A) Determine whether to use Poisson or Negative binomial statistical distribution based on null model and likelihood ratio test(Requires lme4, pscl, and MASS packages);Description of methods for running Likelihood ratio tests and calculating Chi-Square statistics/probabilities is at: http://stats.stackexchange.com/questions/127505/compare-poisson-and-negative-binomial-regression-with-lr-test

SpringLowRawFatal_POISSON <- glm(SpringLowRawFatal ~ 1, family = "poisson") ##Runs Poisson model on the null model (Requires lme4 package)

SpringLowRawFatal_NEGBIN <- glm.nb(SpringLowRawFatal ~ 1) ##Runs Negative Binomial model on the null model (requires MASS package)

##Likelihood ratio test to determine if Negative Binomial fits better than Poisson

2 * (logLik(SpringLowRawFatal_NEGBIN) - logLik(SpringLowRawFatal_POISSON)) ##Returns Chi-square statistic for comparison of two models

pchisq(2 * (logLik(SpringLowRawFatal_NEGBIN) - logLik(SpringLowRawFatal_POISSON)), df = 1, lower.tail = FALSE) ##Returns probability of chi-square statistic for comparison of two models

#Negative Binomial Supported

###(B) For strongly correlated predictor variable pairs, identify which more strongly associated with response variable

###HeightM and GlassAreaSqM

cor(HeightM, SpringLowRawFatal) ## r = 0.32

cor(GlassAreaSqM, SpringLowRawFatal) ## r = 0.41

##Retain GlassAreaSqM (exclude HeightM)

###GlassAreaSqM and AreaLight

cor(GlassAreaSqM, SpringLowRawFatal) ## r = 0.41

cor(AreaLight, SpringLowRawFatal) ## r = 0.64

##Retain both GlassAreaSqm and AreaLight because of frequent inclusion of window area in past studies just barely uncorrelated with area lit (r=0.698) depending on rounding

###AreaLight and Footprint

cor(AreaLight, SpringLowRawFatal) ## r = 0.64

cor(Footprint, SpringLowRawFatal) ## r = 0.23

##Retain AreaLight (exclude Footprint)

###Vegetation50 and Vegetation100

cor(Vegetation50, SpringLowRawFatal) ## r = 0.66

cor(Vegetation100, SpringLowRawFatal) ## r = 0.65

##Retain Vegetation50 (exclude Vegetation100)

###(C) Conduct model selection exercise using remaining non-correlated predictor variables

###NULL MODEL

SpringLowRawFatal_NULL<- glm.nb(SpringLowRawFatal ~ 1)

###GLOBAL MODEL (Additive terms only)

SpringLowRawFatal_GLOBAL <- glm.nb(SpringLowRawFatal ~ GlassAreaSqM + PropLit + AreaLight + DistanceRiver + Vegetation50) ###Excludes HeightM, Footprint, Vegetation100 for strong correlations

###RUN STEPWISE BACKWARD AIC FUNCTION (Requires MASS Package)

SpringLowRawFatalSTEP <- stepAIC(SpringLowRawFatal_GLOBAL, scope = list(lower = ~1), Trace = FALSE)

SpringLowRawFatalSTEP

###Results in 2 variables remaining (GlassAreaSqM, Vegetation50)

###Investigate Glass Area + Vegetation50 Model

SpringLowRawFatal_GLASS_VEG50 <- glm.nb(SpringLowRawFatal ~ GlassAreaSqM + Vegetation50)

SpringLowRawFatal_GLASS_VEG50

summary(SpringLowRawFatal_GLASS_VEG50) ##Generates coefficient estimates along with Standard errors

confint(SpringLowRawFatal_GLASS_VEG50) ##Generates 95% confidence intervals of coefficient estimates

lm.beta(SpringLowRawFatal_GLASS_VEG50) ##Generatess standardized coefficient values (requires QuantPsyc package)

###(D)Plot effects of supported variables

par(mfrow = c(1,1), mai = c(0.5, 0.6, 0.5, 0.6))

SpringLowRawFatal_GLASS <- glm.nb(SpringLowRawFatal ~ GlassAreaSqM)

plot(GlassAreaSqM, SpringLowRawFatal, xlab = "", ylab = "", col = "black", cex = 2, cex.axis = 2.0, lwd = 2.5)

curve(predict(SpringLowRawFatal_GLASS, data.frame(GlassAreaSqM=x), type="resp"),add=TRUE, col="black", lwd = 3)

SpringLowRawFatal_VEG50 <- glm.nb(SpringLowRawFatal ~ Vegetation50)

plot(Vegetation50, SpringLowRawFatal, xlab = "", ylab = "", col = "black", cex = 2, cex.axis = 2.0, lwd = 2.5)

curve(predict(SpringLowRawFatal_VEG50, data.frame(Vegetation50=x), type="resp"),add=TRUE, col="black", lwd = 3)

##############################################################################

###(4) Model selection analysis (Low Raw Count of FALL Fatal Collisions as Depend. Var.)

##############################################################################

###(A) Determine whether to use Poisson or Negative binomial statistical distribution based on null model and likelihood ratio test(Requires lme4, pscl, and MASS packages);Description of methods for running Likelihood ratio tests and calculating Chi-Square statistics/probabilities is at: http://stats.stackexchange.com/questions/127505/compare-poisson-and-negative-binomial-regression-with-lr-test

FallLowRawFatal_POISSON <- glm(FallLowRawFatal ~ 1, family = "poisson") ##Runs Poisson model on the null model (Requires lme4 package)

FallLowRawFatal_NEGBIN <- glm.nb(FallLowRawFatal ~ 1) ##Runs Negative Binomial model on the null model (requires MASS package)

##Likelihood ratio test to determine if Negative Binomial fits better than Poisson

2 * (logLik(FallLowRawFatal_NEGBIN) - logLik(FallLowRawFatal_POISSON)) ##Returns Chi-square statistic for comparison of two models

pchisq(2 * (logLik(FallLowRawFatal_NEGBIN) - logLik(FallLowRawFatal_POISSON)), df = 1, lower.tail = FALSE) ##Returns probability of chi-square statistic for comparison of two models

##Negative Binomial supported

###(B) For strongly correlated predictor variable pairs, identify which more strongly associated with response variable

###HeightM and GlassAreaSqM

cor(HeightM, FallLowRawFatal) ## r = 0.46

cor(GlassAreaSqM, FallLowRawFatal) ## r = 0.66

##Retain GlassAreaSqM (exclude HeightM)

###GlassAreaSqM and AreaLight

cor(GlassAreaSqM, FallLowRawFatal) ## r = 0.66

cor(AreaLight, FallLowRawFatal) ## r = 0.84

##Retain both GlassAreaSqm and AreaLight because of frequent inclusion of window area in past studies just barely uncorrelated with area lit (r=0.698) depending on rounding

###AreaLight and Footprint

cor(AreaLight, FallLowRawFatal) ## r = 0.84

cor(Footprint, FallLowRawFatal) ## r = 0.52

##Retain AreaLight (exclude Footprint)

###Vegetation50 and Vegetation100

cor(Vegetation50, FallLowRawFatal) ## r = 0.79

cor(Vegetation100, FallLowRawFatal) ## r = 0.74

##Retain Vegetation50 (exclude Vegetation100)

###(C) Conduct model selection exercise using remaining non-correlated predictor variables

###NULL MODEL

FallLowRawFatal_NULL<- glm.nb(FallLowRawFatal ~ 1)

###GLOBAL MODEL (Additive terms only)

FallLowRawFatal_GLOBAL <- glm.nb(FallLowRawFatal ~ GlassAreaSqM + PropLit + AreaLight + DistanceRiver + Vegetation50) ###Excludes HeightM, Footprint, Vegetation100 for strong correlations

###RUN STEPWISE BACKWARD AIC FUNCTION (Requires MASS Package)

FallLowRawFatalSTEP <- stepAIC(FallLowRawFatal_GLOBAL, scope = list(lower = ~1), Trace = FALSE)

FallLowRawFatalSTEP

###Results in 2 variables remaining (GlassAreaSqM and Vegetation50)

###Window Area + Vegetation50 Model

FallLowRawFatal_GLASS_VEG50 <- glm.nb(FallLowRawFatal ~ GlassAreaSqM + Vegetation50)

FallLowRawFatal_GLASS_VEG50

summary(FallLowRawFatal_GLASS_VEG50) ##Generates coefficient estimates along with Standard errors

confint(FallLowRawFatal_GLASS_VEG50) ##Generates 95% confidence intervals of coefficient estimates

lm.beta(FallLowRawFatal_GLASS_VEG50) ##Generatess standardized coefficient values (requires QuantPsyc package)

###(D)Plot effects of supported variables

par(mfrow = c(1,1), mai = c(0.5, 0.6, 0.5, 0.6))

FallLowRawFatal_GLASS <- glm.nb(FallLowRawFatal ~ GlassAreaSqM)

plot(GlassAreaSqM, FallLowRawFatal, xlab = "", ylab = "", col = "black", cex = 2, cex.axis = 2.0, lwd = 2.5)

curve(predict(FallLowRawFatal_GLASS, data.frame(GlassAreaSqM=x), type="resp"),add=TRUE, col="black", lwd = 3)

FallLowRawFatal_VEG50 <- glm.nb(FallLowRawFatal ~ Vegetation50)

plot(Vegetation50, FallLowRawFatal, xlab = "", ylab = "", col = "black", cex = 2, cex.axis = 2.0, lwd = 2.5)

curve(predict(FallLowRawFatal_VEG50, data.frame(Vegetation50=x), type="resp"),add=TRUE, col="black", lwd = 3)

##############################################################################

###(5)Model selection analysis (Low Raw Count of TOTAL White-throated Sparrow Fatal Collisions as Dep. var)

##############################################################################

###(A) Determine whether to use Poisson or Negative binomial statistical distribution based on null model and likelihood ratio test(Requires lme4, pscl, and MASS packages);Description of methods for running Likelihood ratio tests and calculating Chi-Square statistics/probabilities is at: http://stats.stackexchange.com/questions/127505/compare-poisson-and-negative-binomial-regression-with-lr-test

WTSPLowRawFatal_POISSON <- glm(WTSPLowRawFatal ~ 1, family = "poisson") ##Runs Poisson model on the null model (Requires lme4 package)

WTSPLowRawFatal_NEGBIN <- glm.nb(WTSPLowRawFatal~ 1) ##Runs Negative Binomial model on the null model (requires MASS package)

##Likelihood ratio test to determine if Negative Binomial fits better than Poisson

2 * (logLik(WTSPLowRawFatal_NEGBIN) - logLik(WTSPLowRawFatal_POISSON)) ##Returns Chi-square statistic for comparison of two models

pchisq(2 * (logLik(WTSPLowRawFatal_NEGBIN) - logLik(WTSPLowRawFatal_POISSON)), df = 1, lower.tail = FALSE) ##Returns probability of chi-square statistic for comparison of two models

#Negative binomial supported

###(B) For strongly correlated predictor variable pairs, identify which more strongly associated with response variable

###HeightM and GlassAreaSqM

cor(HeightM, WTSPLowRawFatal) ## r = 0.31

cor(GlassAreaSqM, WTSPLowRawFatal) ## r = 0.33

##Retain GlassAreaSqM (exclude HeightM)

###GlassAreaSqM and AreaLight

cor(GlassAreaSqM, WTSPLowRawFatal) ## r = 0.33

cor(AreaLight, WTSPLowRawFatal) ## r = 0.58

##Retain both GlassAreaSqm and AreaLight because of frequent inclusion of window area in past studies just barely uncorrelated with area lit (r=0.698) depending on rounding

###AreaLight and Footprint

cor(AreaLight, WTSPLowRawFatal) ## r = 0.58

cor(Footprint, WTSPLowRawFatal) ## r = 0.19

##Retain AreaLight (exclude Footprint)

###Vegetation50 and Vegetation100

cor(Vegetation50, WTSPLowRawFatal) ## r = 0.79

cor(Vegetation100, WTSPLowRawFatal) ## r = 0.71

##Retain Undevloped50 (exclude Vegetation100)

###(C) Conduct model selection exercise using remaining non-correlated predictor variables

###NULL MODEL

WTSPLowRawFatal_NULL<- glm.nb(WTSPLowRawFatal ~ 1)

###GLOBAL MODEL (Additive terms only)

WTSPLowRawFatal_GLOBAL <- glm.nb(WTSPLowRawFatal ~ GlassAreaSqM + PropLit + AreaLight + DistanceRiver + Vegetation50) ###Excludes HeightM, Footprint, Vegetation100 for strong correlations

###RUN STEPWISE BACKWARD AIC FUNCTION (Requires MASS Package)

WTSPLowRawFatalSTEP <- stepAIC(WTSPLowRawFatal_GLOBAL, scope = list(lower = ~1), Trace = FALSE)

WTSPLowRawFatalSTEP

###Results in 2 variables remaining (GlassAreaSqM and Vegetation50)

###Insepct Glass Area + Vegetation50 Model

WTSPLowRawFatal_GLASS_VEG50 <- glm.nb(WTSPLowRawFatal ~ GlassAreaSqM + Vegetation50)

WTSPLowRawFatal_GLASS_VEG50

summary(WTSPLowRawFatal_GLASS_VEG50) ##Generates coefficient estimates along with Standard errors

confint(WTSPLowRawFatal_GLASS_VEG50) ##Generates 95% confidence intervals of coefficient estimates

lm.beta(WTSPLowRawFatal_GLASS_VEG50) ##Generatess standardized coefficient values (requires QuantPsyc package)

###(D)Plot effects of supported variables

par(mfrow = c(1,1), mai = c(0.5, 0.6, 0.5, 0.6))

WTSPLowRawFatal_GLASS <- glm.nb(WTSPLowRawFatal ~ GlassAreaSqM)

plot(GlassAreaSqM, WTSPLowRawFatal, xlab = "", ylab = "", col = "black", cex = 2, cex.axis = 2.0, lwd = 2.5)

curve(predict(WTSPLowRawFatal_GLASS, data.frame(GlassAreaSqM=x), type="resp"),add=TRUE, col="black", lwd = 3)

WTSPLowRawFatal_VEG50 <- glm.nb(WTSPLowRawFatal ~ Vegetation50)

plot(Vegetation50, WTSPLowRawFatal, xlab = "", ylab = "", col = "black", cex = 2, cex.axis = 2.0, lwd = 2.5)

curve(predict(WTSPLowRawFatal_VEG50, data.frame(Vegetation50=x), type="resp"),add=TRUE, col="black", lwd = 3)

##############################################################################

###(6)Model selection analysis (Low Raw Count of TOTAL Nashville Warbler Fatal Collisions as Dep var

##############################################################################

###(A) Determine whether to use Poisson or Negative binomial statistical distribution based on null model and likelihood ratio test(Requires lme4, pscl, and MASS packages);Description of methods for running Likelihood ratio tests and calculating Chi-Square statistics/probabilities is at: http://stats.stackexchange.com/questions/127505/compare-poisson-and-negative-binomial-regression-with-lr-test

NAWALowRawFatal_POISSON <- glm(NAWALowRawFatal ~ 1, family = "poisson") ##Runs Poisson model on the null model (Requires lme4 package)

NAWALowRawFatal_NEGBIN <- glm.nb(NAWALowRawFatal ~ 1) ##Runs Negative Binomial model on the null model (requires MASS package)

##Likelihood ratio test to determine if Negative Binomial fits better than Poisson

2 * (logLik(NAWALowRawFatal_NEGBIN) - logLik(NAWALowRawFatal_POISSON)) ##Returns Chi-square statistic for comparison of two models

pchisq(2 * (logLik(NAWALowRawFatal_NEGBIN) - logLik(NAWALowRawFatal_POISSON)), df = 1, lower.tail = FALSE) ##Returns probability of chi-square statistic for comparison of two models

#Negative binomial supported

###(B) For strongly correlated predictor variable pairs, identify which more strongly associated with response variable

###HeightM and GlassAreaSqM

cor(HeightM, NAWALowRawFatal) ## r = 0.37

cor(GlassAreaSqM, NAWALowRawFatal) ## r = 0.62

##Retain GlassAreaSqM (exclude HeightM)

###GlassAreaSqM and AreaLight

cor(GlassAreaSqM, NAWALowRawFatal) ## r = 0.62

cor(AreaLight, NAWALowRawFatal) ## r = 0.92

##Retain both GlassAreaSqm and AreaLight because of frequent inclusion of window area in past studies just barely uncorrelated with area lit (r=0.698) depending on rounding

###AreaLight and Footprint

cor(AreaLight, NAWALowRawFatal) ## r = 0.92

cor(Footprint, NAWALowRawFatal) ## r = 0.66

##Retain AreaLight (exclude Footprint)

###Vegetation50 and Vegetation100

cor(Vegetation50, NAWALowRawFatal) ## r = 0.76

cor(Vegetation100, NAWALowRawFatal) ## r = 0.72

##Retain Vegetation50 (exclude Vegetation100)

###(C) Conduct model selection exercise using remaining non-correlated predictor variables

###NULL MODEL

NAWALowRawFatal_NULL<- glm.nb(NAWALowRawFatal ~ 1)

###GLOBAL MODEL (Additive terms only)

NAWALowRawFatal_GLOBAL <- glm.nb(NAWALowRawFatal ~ GlassAreaSqM + PropLit + AreaLight + DistanceRiver + Vegetation50) ###Excludes HeightM, Footprint, Vegetation100 for strong correlations

###RUN STEPWISE BACKWARD AIC FUNCTION (Requires MASS Package)

NAWALowRawFatalSTEP <- stepAIC(NAWALowRawFatal_GLOBAL, scope = list(lower = ~1), Trace = FALSE)

NAWALowRawFatalSTEP

###Results in 3 variables remaining (GlassAreaSqM, DistanceRiver, and Vegetation50)

###Inspect Glass Area + DistanceRiver + Vegetation50 Model

NAWALowRawFatal_GLASS_DISTRIV_VEG50 <- glm.nb(NAWALowRawFatal ~ GlassAreaSqM + DistanceRiver + Vegetation50) ###Excludes HeightM and Vegetation50 for strong correlations with GlassAreaSqM and Vegetation50 respectively

NAWALowRawFatal_GLASS_DISTRIV_VEG50

summary(NAWALowRawFatal_GLASS_DISTRIV_VEG50) ##Generates coefficient estimates along with Standard errors

confint(NAWALowRawFatal_GLASS_DISTRIV_VEG50) ##Generates 95% confidence intervals of coefficient estimates

lm.beta(NAWALowRawFatal_GLASS_DISTRIV_VEG50) ##Generatess standardized coefficient values (requires QuantPsyc package)

##Above model indicates 95% CI of DistRiver overlaps zero so do not further include it

###(D)Plot effects of supported variables

par(mfrow = c(1,1), mai = c(0.5, 0.6, 0.5, 0.6))

NAWALowRawFatal_GLASS <- glm.nb(NAWALowRawFatal ~ GlassAreaSqM)

plot(GlassAreaSqM, NAWALowRawFatal, xlab = "", ylab = "", col = "black", cex = 2, cex.axis = 2.0, lwd = 2.5)

curve(predict(NAWALowRawFatal_GLASS, data.frame(GlassAreaSqM=x), type="resp"),add=TRUE, col="black", lwd = 3)

NAWALowRawFatal_VEG50 <- glm.nb(NAWALowRawFatal ~ Vegetation50)

plot(Vegetation50, NAWALowRawFatal, xlab = "", ylab = "", col = "black", cex = 2, cex.axis = 2.0, lwd = 2.5)

curve(predict(NAWALowRawFatal_VEG50, data.frame(Vegetation50=x), type="resp"),add=TRUE, col="black", lwd = 3)

##############################################################################

###(7)Model selection analysis (Low Raw Count of TOTAL Ovenbird Fatal Collisions as Dep. var.

##############################################################################

###(A) Determine whether to use Poisson or Negative binomial statistical distribution based on null model and likelihood ratio test(Requires lme4, pscl, and MASS packages);Description of methods for running Likelihood ratio tests and calculating Chi-Square statistics/probabilities is at: http://stats.stackexchange.com/questions/127505/compare-poisson-and-negative-binomial-regression-with-lr-test

OVENLowRawFatal_POISSON <- glm(OVENLowRawFatal ~ 1, family = "poisson") ##Runs Poisson model on the null model (Requires lme4 package)

OVENLowRawFatal_NEGBIN <- glm.nb(OVENLowRawFatal ~ 1) ##Runs Negative Binomial model on the null model (requires MASS package)

##Likelihood ratio test to determine if Negative Binomial fits better than Poisson

2 * (logLik(OVENLowRawFatal_NEGBIN) - logLik(OVENLowRawFatal_POISSON)) ##Returns Chi-square statistic for comparison of two models

pchisq(2 * (logLik(OVENLowRawFatal_NEGBIN) - logLik(OVENLowRawFatal_POISSON)), df = 1, lower.tail = FALSE) ##Returns probability of chi-square statistic for comparison of two models

#Negative binomial supported

###(B) For strongly correlated predictor variable pairs, identify which more strongly associated with response variable

###HeightM and GlassAreaSqM

cor(HeightM, OVENLowRawFatal) ## r = 0.40

cor(GlassAreaSqM, OVENLowRawFatal) ## r = 0.43

##Retain GlassAreaSqM (exclude HeightM)

###GlassAreaSqM and AreaLight

cor(GlassAreaSqM, OVENLowRawFatal) ## r = 0.43

cor(AreaLight, OVENLowRawFatal) ## r = 0.53

##Retain both GlassAreaSqm and AreaLight because of frequent inclusion of window area in past studies just barely uncorrelated with area lit (r=0.698) depending on rounding

###AreaLight and Footprint

cor(AreaLight, OVENLowRawFatal) ## r = 0.53

cor(Footprint, OVENLowRawFatal) ## r = 0.07

##Retain AreaLight (exclude Footprint)

###Vegetation50 and Vegetation100

cor(Vegetation50, OVENLowRawFatal) ## r = 0.57

cor(Vegetationd100, OVENLowRawFatal) ## r = 0.55

##Retain Undevloped50 (exclude Vegetation100)

###(C) Conduct model selection exercise using remaining non-correlated predictor variables

###NULL MODEL

OVENLowRawFatal_NULL<- glm.nb(OVENLowRawFatal ~ 1)

###GLOBAL MODEL (Additive terms only)

OVENLowRawFatal_GLOBAL <- glm.nb(OVENLowRawFatal ~ GlassAreaSqM + PropLit + AreaLight + DistanceRiver + Vegetation50) ###Excludes HeightM, Footprint, Vegetation100 for strong correlations

###RUN STEPWISE BACKWARD AIC FUNCTION (Requires MASS Package)

OVENLowRawFatalSTEP <- stepAIC(OVENLowRawFatal_GLOBAL, scope = list(lower = ~1), Trace = FALSE, k=2)

OVENLowRawFatalSTEP

###Results in 2 variables remaining (GlassAreaSqM and Vegetation50)

###Inspect Glass Area + Vegetation50 Model

OVENLowRawFatal_GLASS_VEG50 <- glm.nb(OVENLowRawFatal ~ GlassAreaSqM + Vegetation50)

OVENLowRawFatal_GLASS_VEG50

summary(OVENLowRawFatal_GLASS_VEG50) ##Generates coefficient estimates along with Standard errors

confint(OVENLowRawFatal_GLASS_VEG50) ##Generates 95% confidence intervals of coefficient estimates

lm.beta(OVENLowRawFatal_GLASS_VEG50) ##Generatess standardized coefficient values (requires QuantPsyc package)

###(D)Plot effects of supported variables

par(mfrow = c(1,1), mai = c(0.5, 0.6, 0.5, 0.6))

OVENLowRawFatal_GLASS <- glm.nb(OVENLowRawFatal ~ GlassAreaSqM)

plot(GlassAreaSqM, OVENLowRawFatal, xlab = "", ylab = "", col = "black", cex = 2, cex.axis = 2.0, lwd = 2.5)

curve(predict(OVENLowRawFatal_GLASS, data.frame(GlassAreaSqM=x), type="resp"),add=TRUE, col="black", lwd = 3)

OVENLowRawFatal_VEG50 <- glm.nb(OVENLowRawFatal ~ Vegetation50)

plot(Vegetation50, OVENLowRawFatal, xlab = "", ylab = "", col = "black", cex = 2, cex.axis = 2.0, lwd = 2.5)

curve(predict(OVENLowRawFatal_VEG50, data.frame(Vegetation50=x), type="resp"),add=TRUE, col="black", lwd = 3)

##############################################################################

###(8)Model selection analysis (Low Raw Count of TOTAL Common Yellowthroat Fatal Collisions as Dep. var.

##############################################################################

###(A) Determine whether to use Poisson or Negative binomial statistical distribution based on null model and likelihood ratio test(Requires lme4, pscl, and MASS packages);Description of methods for running Likelihood ratio tests and calculating Chi-Square statistics/probabilities is at: http://stats.stackexchange.com/questions/127505/compare-poisson-and-negative-binomial-regression-with-lr-test

COYELowRawFatal_POISSON <- glm(COYELowRawFatal ~ 1, family = "poisson") ##Runs Poisson model on the null model (Requires lme4 package)

COYELowRawFatal_NEGBIN <- glm.nb(COYELowRawFatal ~ 1) ##Runs Negative Binomial model on the null model (requires MASS package)

##Likelihood ratio test to determine if Negative Binomial fits better than Poisson

2 * (logLik(COYELowRawFatal_NEGBIN) - logLik(COYELowRawFatal_POISSON)) ##Returns Chi-square statistic for comparison of two models

pchisq(2 * (logLik(COYELowRawFatal_NEGBIN) - logLik(COYELowRawFatal_POISSON)), df = 1, lower.tail = FALSE) ##Returns probability of chi-square statistic for comparison of two models

#Negative binomial supported

###(B) For strongly correlated predictor variable pairs, identify which more strongly associated with response variable

###HeightM and GlassAreaSqM

cor(HeightM, COYELowRawFatal) ## r = 0.34

cor(GlassAreaSqM, COYELowRawFatal) ## r = 0.56

##Retain GlassAreaSqM (exclude HeightM)

###GlassAreaSqM and AreaLight

cor(GlassAreaSqM, COYELowRawFatal) ## r = 0.56

cor(AreaLight, COYELowRawFatal) ## r = 0.83

##Retain both GlassAreaSqm and AreaLight because of frequent inclusion of window area in past studies just barely uncorrelated with area lit (r=0.698) depending on rounding

###AreaLight and Footprint

cor(AreaLight, COYELowRawFatal) ## r = 0.83

cor(Footprint, COYELowRawFatal) ## r = 0.56

##Retain AreaLight (exclude Footprint)

###Vegetation50 and Vegetation100

cor(Vegetation50, COYELowRawFatal) ## r = 0.76

cor(Vegetation100, COYELowRawFatal) ## r = 0.81

##Retain Vegetation100 (exclude Vegetation50)

###(C) Conduct model selection exercise using remaining non-correlated predictor variables

###NULL MODEL

COYELowRawFatal_NULL <- glm.nb(COYELowRawFatal ~ 1)

###GLOBAL MODEL (Additive terms only)

COYELowRawFatal_GLOBAL <- glm.nb(COYELowRawFatal ~ GlassAreaSqM + PropLit + AreaLight + DistanceRiver + Vegetation100) ###Excludes HeightM, Footprint, Vegetation50 for strong correlations

###RUN STEPWISE BACKWARD AIC FUNCTION (Requires MASS Package)

COYELowRawFatalSTEP <- stepAIC(COYELowRawFatal_GLOBAL, scope = list(lower = ~1), Trace = FALSE)

COYELowRawFatalSTEP

###Results in 3 variables remaining (GlassAreaSqM, PropLit, and Vegetation100)

###Inspect Glass Area + PropLit + Vegetation100 Model

COYELowRawFatal_GLASS_PROPLit_VEG100 <- glm.nb(COYELowRawFatal ~ GlassAreaSqM + PropLit + Vegetation100)

COYELowRawFatal_GLASS_PROPLit_VEG100

summary(COYELowRawFatal_GLASS_PROPLit_VEG100) ##Generates coefficient estimates along with Standard errors

confint(COYELowRawFatal_GLASS_PROPLit_VEG100) ##Generates 95% confidence intervals of coefficient estimates

lm.beta(COYELowRawFatal_GLASS_PROPLit_VEG100) ##Generatess standardized coefficient values (requires QuantPsyc package)

##Above model indicates 95% CI of PropLit overlaps zero so do not further include it

###(D)Plot effects of supported variables

par(mfrow = c(1,1), mai = c(0.5, 0.6, 0.5, 0.6))

COYELowRawFatal_GLASS <- glm.nb(COYELowRawFatal ~ GlassAreaSqM)

plot(GlassAreaSqM, COYELowRawFatal, xlab = "", ylab = "", col = "black", cex = 2, cex.axis = 2.0, lwd = 2.5)

curve(predict(COYELowRawFatal_GLASS, data.frame(GlassAreaSqM=x), type="resp"),add=TRUE, col="black", lwd = 3)

COYELowRawFatal_VEG100 <- glm.nb(COYELowRawFatal ~ Vegetation100)

plot(Vegetation100, COYELowRawFatal, xlab = "", ylab = "", col = "black", cex = 2, cex.axis = 2.0, lwd = 2.5)

curve(predict(COYELowRawFatal_VEG100, data.frame(Vegetation100=x), type="resp"),add=TRUE, col="black", lwd = 3)

##############################################################################

###(9)Model selection analysis (Low Raw Count of TOTAL Tennessee Warbler Fatal Collisions as Dep. var

##############################################################################

###(A) Determine whether to use Poisson or Negative binomial statistical distribution based on null model and likelihood ratio test(Requires lme4, pscl, and MASS packages);Description of methods for running Likelihood ratio tests and calculating Chi-Square statistics/probabilities is at: http://stats.stackexchange.com/questions/127505/compare-poisson-and-negative-binomial-regression-with-lr-test

TEWALowRawFatal_POISSON <- glm(TEWALowRawFatal ~ 1, family = "poisson") ##Runs Poisson model on the null model (Requires lme4 package)

TEWADeadLowRaw_NEGBIN <- glm.nb(TEWALowRawFatal ~ 1) ##Runs Negative Binomial model on the null model (requires MASS package)

##Likelihood ratio test to determine if Negative Binomial fits better than Poisson

2 * (logLik(TEWADeadLowRaw_NEGBIN) - logLik(TEWALowRawFatal_POISSON)) ##Returns Chi-square statistic for comparison of two models

pchisq(2 * (logLik(TEWADeadLowRaw_NEGBIN) - logLik(TEWALowRawFatal_POISSON)), df = 1, lower.tail = FALSE) ##Returns probability of chi-square statistic for comparison of two models

#Negative binomial supported

###(B) For strongly correlated predictor variable pairs, identify which more strongly associated with response variable

###HeightM and GlassAreaSqM

cor(HeightM, TEWALowRawFatal) ## r = 0.14

cor(GlassAreaSqM, TEWALowRawFatal) ## r = 0.36

##Retain GlassAreaSqM (exclude HeightM)

###GlassAreaSqM and AreaLight

cor(GlassAreaSqM, TEWALowRawFatal) ## r = 0.36

cor(AreaLight, TEWALowRawFatal) ## r = 0.75

##Retain both GlassAreaSqm and AreaLight because of frequent inclusion of window area in past studies just barely uncorrelated with area lit (r=0.698) depending on rounding

###AreaLight and Footprint

cor(AreaLight, TEWALowRawFatal) ## r = 0.75

cor(Footprint, TEWALowRawFatal) ## r = 0.52

##Retain AreaLight (exclude Footprint)

###Vegetation50 and Vegetation100

cor(Vegetation50, TEWALowRawFatal) ## r = 0.76

cor(Vegetation100, TEWALowRawFatal) ## r = 0.66

##Retain Vegetation50 (exclude exclude)

###(C) Conduct model selection exercise using remaining non-correlated predictor variables

###NULL MODEL

TEWALowRawFatal_NULL<- glm.nb(TEWALowRawFatal ~ 1)

###GLOBAL MODEL (Additive terms only)

TEWALowRawFatal_GLOBAL <- glm.nb(TEWALowRawFatal ~ GlassAreaSqM + PropLit + AreaLight + DistanceRiver + Vegetation50) ###Excludes HeightM, Footprint, Vegetation100 for strong correlations

###RUN STEPWISE BACKWARD AIC FUNCTION (Requires MASS Package)

TEWALowRawFatalSTEP <- stepAIC(TEWALowRawFatal_GLOBAL, scope = list(lower = ~1), Trace = FALSE)

TEWALowRawFatalSTEP

###Results in 1 variable remaining (UndeVegetation50veloped50)

###Investigate Vegetation50 Model

TEWALowRawFatal_VEG50 <- glm.nb(TEWALowRawFatal ~ Vegetation50)

TEWALowRawFatal_VEG50

summary(TEWALowRawFatal_VEG50) ##Generates coefficient estimates along with Standard errors

confint(TEWALowRawFatal_VEG50) ##Generates 95% confidence intervals of coefficient estimates

lm.beta(TEWALowRawFatal_VEG50) ##Generatess standardized coefficient values (requires QuantPsyc package)

#Results in PropLit 95% confidence interval overlapping zero, so only use Vegetation50

###(D)Plot effects of supported variables

par(mfrow = c(1,1), mai = c(0.5, 0.6, 0.5, 0.6))

TEWALowRawFatal_VEG50 <- glm.nb(TEWALowRawFatal ~ Vegetation50)

plot(Vegetation50, TEWALowRawFatal, xlab = "", ylab = "", col = "black", cex = 2, cex.axis = 2.0, lwd = 2.5)

curve(predict(TEWALowRawFatal_VEG50, data.frame(Vegetation50=x), type="resp"),add=TRUE, col="black", lwd = 3)

##############################################################################

###(10 Model selection analysis (Number of Speices Colliding across entire study as Dep. var.)

##############################################################################

###(A) Determine whether to use Poisson or Negative binomial statistical distribution based on null model and likelihood ratio test(Requires lme4, pscl, and MASS packages);Description of methods for running Likelihood ratio tests and calculating Chi-Square statistics/probabilities is at: http://stats.stackexchange.com/questions/127505/compare-poisson-and-negative-binomial-regression-with-lr-test

SpeciesAll_POISSON <- glm(SpeciesAll ~ 1, family = "poisson") ##Runs Poisson model on the null model (Requires lme4 package)

SpeciesAll_NEGBIN <- glm.nb(SpeciesAll ~ 1) ##Runs Negative Binomial model on the null model (requires MASS package)

##Likelihood ratio test to determine if Negative Binomial fits better than Poisson

2 * (logLik(SpeciesAll_NEGBIN) - logLik(SpeciesAll_POISSON)) ##Returns Chi-square statistic for comparison of two models

pchisq(2 * (logLik(SpeciesAll_NEGBIN) - logLik(SpeciesAll_POISSON)), df = 1, lower.tail = FALSE) ##Returns probability of chi-square statistic for comparison of two models

#Negative binomial supported

###(B) For strongly correlated predictor variable pairs, identify which more strongly associated with response variable

###HeightM and GlassAreaSqM

cor(HeightM, SpeciesAll) ## r = 0.59

cor(GlassAreaSqM, SpeciesAll) ## r = 0.77

##Retain GlassAreaSqM (exclude HeightM)

###GlassAreaSqM and AreaLight

cor(GlassAreaSqM, SpeciesAll) ## r = 0.77

cor(AreaLight, SpeciesAll) ## r = 0.78

##Retain both GlassAreaSqm and AreaLight because of frequent inclusion of window area in past studies just barely uncorrelated with area lit (r=0.698) depending on rounding

###AreaLight and Footprint

cor(AreaLight, SpeciesAll) ## r = 0.78

cor(Footprint, SpeciesAll) ## r = 0.49

##Retain AreaLight (exclude Footprint)

###Vegetation50 and Vegetation100

cor(Vegetation50, SpeciesAll) ## r = 0.75

cor(Vegetation100, SpeciesAll) ## r = 0.72

##Retain Vegetation50 (exclude Vegetation100)

###(C) Conduct model selection exercise using remaining non-correlated predictor variables

###NULL MODEL

SpeciesAll_NULL<- glm.nb(SpeciesAll ~ 1)

###GLOBAL MODEL (Additive terms only)

SpeciesAll_GLOBAL <- glm.nb(SpeciesAll ~ GlassAreaSqM + PropLit + AreaLight + DistanceRiver + Vegetation50) ###Excludes HeightM, Footprint, Vegetation100 for strong correlations

###RUN STEPWISE BACKWARD AIC FUNCTION (Requires MASS Package)

SpeciesAllSTEP <- stepAIC(SpeciesAll_GLOBAL, scope = list(lower = ~1), Trace = FALSE)

SpeciesAllSTEP

###Results in 5 variables remaining (GlassAreaSqM, PropLit, AreaLight, DistanceRiver and Vegetation50)

###Inspect this 5 variable model

SpeciesAll_GLOBAL <- glm.nb(SpeciesAll ~ GlassAreaSqM + PropLit + AreaLight + DistanceRiver + Vegetation50)

SpeciesAll_GLOBAL

summary(SpeciesAll_GLOBAL) ##Generates coefficient estimates along with Standard errors

confint(SpeciesAll_GLOBAL) ##Generates 95% confidence intervals of coefficient estimates

lm.beta(SpeciesAll_GLOBAL) ##Generatess standardized coefficient values (requires QuantPsyc package)

#Results in 95% CIs for AreaLight and DistRiver to overlap zero so only go with remaining 3

###(D)Plot effects of supported variables

par(mfrow = c(1,1), mai = c(0.5, 0.6, 0.5, 0.6))

SpeciesAll_GLASS <- glm.nb(SpeciesAll ~ GlassAreaSqM)

plot(GlassAreaSqM, SpeciesAll, xlab = "", ylab = "", col = "black", cex = 2, cex.axis = 2.0, lwd = 2.5)

curve(predict(SpeciesAll_GLASS, data.frame(GlassAreaSqM=x), type="resp"),add=TRUE, col="black", lwd = 3)

SpeciesAll_PROPLit <- glm.nb(SpeciesAll ~ PropLit)

plot(PropLit, SpeciesAll, xlab = "", ylab = "", col = "black", cex = 2, cex.axis = 2.0, lwd = 2.5)

curve(predict(SpeciesAll_PROPLit, data.frame(PropLit=x), type="resp"),add=TRUE, col="black", lwd = 3)

SpeciesAll_VEG50 <- glm.nb(SpeciesAll ~ Vegetation50)

plot(Vegetation50, SpeciesAll, xlab = "", ylab = "", col = "black", cex = 2, cex.axis = 2.0, lwd = 2.5)

curve(predict(SpeciesAll_VEG50, data.frame(Vegetation50=x), type="resp"),add=TRUE, col="black", lwd = 3)

##############################################################################

###(11)Model selection analysis (Number of Speices Colliding in spring as Dependent variable

##############################################################################

###(A) Determine whether to use Poisson or Negative binomial statistical distribution based on null model and likelihood ratio test(Requires lme4, pscl, and MASS packages);Description of methods for running Likelihood ratio tests and calculating Chi-Square statistics/probabilities is at: http://stats.stackexchange.com/questions/127505/compare-poisson-and-negative-binomial-regression-with-lr-test

SpeciesAllSpring_POISSON <- glm(SpeciesAllSpring ~ 1, family = "poisson") ##Runs Poisson model on the null model (Requires lme4 package)

SpeciesAllSpring_NEGBIN <- glm.nb(SpeciesAllSpring ~ 1) ##Runs Negative Binomial model on the null model (requires MASS package)

##Likelihood ratio test to determine if Negative Binomial fits better than Poisson

2 * (logLik(SpeciesAllSpring_NEGBIN) - logLik(SpeciesAllSpring_POISSON)) ##Returns Chi-square statistic for comparison of two models

pchisq(2 * (logLik(SpeciesAllSpring_NEGBIN) - logLik(SpeciesAllSpring_POISSON)), df = 1, lower.tail = FALSE) ##Returns probability of chi-square statistic for comparison of two models

#Negative binomial supported

###(B) For strongly correlated predictor variable pairs, identify which more strongly associated with response variable

###HeightM and GlassAreaSqM

cor(HeightM, SpeciesAllSpring) ## r = 0.39

cor(GlassAreaSqM, SpeciesAllSpring) ## r = 0.60

##Retain GlassAreaSqM (exclude HeightM)

###GlassAreaSqM and AreaLight

cor(GlassAreaSqM, SpeciesAllSpring) ## r = 0.60

cor(AreaLight, SpeciesAllSpring) ## r = 0.83

##Retain both GlassAreaSqm and AreaLight because of frequent inclusion of window area in past studies just barely uncorrelated with area lit (r=0.698) depending on rounding

###AreaLight and Footprint

cor(AreaLight, SpeciesAllSpring) ## r = 0.83

cor(Footprint, SpeciesAllSpring) ## r = 0.55

##Retain AreaLight (exclude Footprint)

###Vegetation50 and Vegetation100

cor(Vegetation50, SpeciesAllSpring) ## r = 0.79

cor(Vegetation100, SpeciesAllSpring) ## r = 0.80

##Retain Undevloped100 (exclude Vegetation50)

###(C) Conduct model selection exercise using remaining non-correlated predictor variables

###NULL MODEL

SpeciesAllSpring_NULL<- glm.nb(SpeciesAllSpring ~ 1)

###GLOBAL MODEL (Additive terms only)

SpeciesAllSpring_GLOBAL <- glm.nb(SpeciesAllSpring ~ GlassAreaSqM + PropLit + AreaLight + DistanceRiver + Vegetation100) ###Excludes HeightM, Footprint, Vegetation50 for strong correlations

###RUN STEPWISE BACKWARD AIC FUNCTION (Requires MASS Package)

SpeciesAllSpringSTEP <- stepAIC(SpeciesAllSpring_GLOBAL, scope = list(lower = ~1), Trace = FALSE)

SpeciesAllSpringSTEP

###Results in 4 variables remaining (GlassAreaSqM, PropLit, AreaLight and Vegetation100)

###Investigate GlassAreaSqM + PropLit + Area Light + Vegetation100 Model

SpeciesAllSpring_GLASS_PROPLit_AREALight_VEG100 <- glm.nb(SpeciesAllSpring ~ GlassAreaSqM + PropLit + AreaLight + Vegetation100)

SpeciesAllSpring_GLASS_PROPLit_AREALight_VEG100

summary(SpeciesAllSpring_GLASS_PROPLit_AREALight_VEG100) ##Generates coefficient estimates along with Standard errors

confint(SpeciesAllSpring_GLASS_PROPLit_AREALight_VEG100) ##Generates 95% confidence intervals of coefficient estimates

lm.beta(SpeciesAllSpring_GLASS_PROPLit_AREALight_VEG100) ##Generatess standardized coefficient values (requires QuantPsyc package)

#Results in 95% CIs for AreaLight to overlap zero so only go with remaining variables

###(D)Plot effects of supported variables

par(mfrow = c(1,1), mai = c(0.5, 0.6, 0.5, 0.6))

SpeciesAllSpring_GLASS <- glm.nb(SpeciesAllSpring ~ GlassAreaSqM)

plot(GlassAreaSqM, SpeciesAllSpring, xlab = "", ylab = "", col = "black", cex = 2, cex.axis = 2.0, lwd = 2.5)

curve(predict(SpeciesAllSpring_GLASS, data.frame(GlassAreaSqM=x), type="resp"),add=TRUE, col="black", lwd = 3)

SpeciesAllSpring_PROPLit <- glm.nb(SpeciesAllSpring ~ PropLit)

plot(PropLit, SpeciesAllSpring, xlab = "", ylab = "", col = "black", cex = 2, cex.axis = 2.0, lwd = 2.5)

curve(predict(SpeciesAllSpring_PROPLit, data.frame(PropLit=x), type="resp"),add=TRUE, col="black", lwd = 3)

SpeciesAllSpring_VEG100 <- glm.nb(SpeciesAllSpring ~ Vegetation100)

plot(Vegetation100, SpeciesAllSpring, xlab = "", ylab = "", col = "black", cex = 2, cex.axis = 2.0, lwd = 2.5)

curve(predict(SpeciesAllSpring_VEG100, data.frame(Vegetation100=x), type="resp"),add=TRUE, col="black", lwd = 3)

##############################################################################

###(12)Model selection analysis (Number of Speices Colliding in fall as Dependent variable

##############################################################################

###(A) Determine whether to use Poisson or Negative binomial statistical distribution based on null model and likelihood ratio test(Requires lme4, pscl, and MASS packages);Description of methods for running Likelihood ratio tests and calculating Chi-Square statistics/probabilities is at: http://stats.stackexchange.com/questions/127505/compare-poisson-and-negative-binomial-regression-with-lr-test

SpeciesAllFall_POISSON <- glm(SpeciesAllFall ~ 1, family = "poisson") ##Runs Poisson model on the null model (Requires lme4 package)

SpeciesAllFall_NEGBIN <- glm.nb(SpeciesAllFall ~ 1) ##Runs Negative Binomial model on the null model (requires MASS package)

##Likelihood ratio test to determine if Negative Binomial fits better than Poisson

2 * (logLik(SpeciesAllFall_NEGBIN) - logLik(SpeciesAllFall_POISSON)) ##Returns Chi-square statistic for comparison of two models

pchisq(2 * (logLik(SpeciesAllFall_NEGBIN) - logLik(SpeciesAllFall_POISSON)), df = 1, lower.tail = FALSE) ##Returns probability of chi-square statistic for comparison of two models #Negative binomial supported

###(B) For strongly correlated predictor variable pairs, identify which more strongly associated with response variable

###HeightM and GlassAreaSqM

cor(HeightM, SpeciesAllFall) ## r = 0.62

cor(GlassAreaSqM, SpeciesAllFall) ## r = 0.82

##Retain GlassAreaSqM (exclude HeightM)

###GlassAreaSqM and AreaLight

cor(GlassAreaSqM, SpeciesAllFall) ## r = 0.82

cor(AreaLight, SpeciesAllFall) ## r = 0.76

##Retain both GlassAreaSqm and AreaLight because of frequent inclusion of window area in past studies just barely uncorrelated with area lit (r=0.698) depending on rounding

###AreaLight and Footprint

cor(AreaLight, SpeciesAllFall) ## r = 0.76

cor(Footprint, SpeciesAllFall) ## r = 0.48

##Retain AreaLight (exclude Footprint)

###Vegetation50 and Vegetation100

cor(Vegetation50, SpeciesAllFall) ## r = 0.70

cor(Vegetation100, SpeciesAllFall) ## r = 0.68

##Retain Undevloped50 (exclude Vegetation100)

###(C) Conduct model selection exercise using remaining non-correlated predictor variables

###NULL MODEL

SpeciesAllFall_NULL<- glm.nb(SpeciesAllFall ~ 1)

###GLOBAL MODEL (Additive terms only)

SpeciesAllFall_GLOBAL <- glm.nb(SpeciesAllFall ~ GlassAreaSqM + PropLit + AreaLight + DistanceRiver + Vegetation50) ####Excludes HeightM, Footprint, Vegetation100 for strong correlations

###RUN STEPWISE BACKWARD AIC FUNCTION (Requires MASS Package)

SpeciesAllFallSTEP <- stepAIC(SpeciesAllFall_GLOBAL, scope = list(lower = ~1), Trace = FALSE)

###Results in 2 variables remaining (GlassAreaSqM and Vegetation100)

###Investigate GlassArea + Vegetation50 Model

SpeciesAllFall_GLASS_VEG50 <- glm.nb(SpeciesAllFall ~ GlassAreaSqM + Vegetation50)

SpeciesAllFall_GLASS_VEG50

summary(SpeciesAllFall_GLASS_VEG50) ##Generates coefficient estimates along with Standard errors

confint(SpeciesAllFall_GLASS_VEG50) ##Generates 95% confidence intervals of coefficient estimates

lm.beta(SpeciesAllFall_GLASS_VEG50) ##Generatess standardized coefficient values (requires QuantPsyc package)

###(D)Plot effects of supported variables

par(mfrow = c(1,1), mai = c(0.5, 0.6, 0.5, 0.6))

SpeciesAllFall_GLASS <- glm.nb(SpeciesAllFall ~ GlassAreaSqM)

plot(GlassAreaSqM, SpeciesAllFall, xlab = "", ylab = "", col = "black", cex = 2, cex.axis = 2.0, lwd = 2.5)

curve(predict(SpeciesAllFall_GLASS, data.frame(GlassAreaSqM=x), type="resp"),add=TRUE, col="black", lwd = 3)

SpeciesAllFall_VEG50 <- glm.nb(SpeciesAllFall ~ Vegetation50)

plot(Vegetation50, SpeciesAllFall, xlab = "", ylab = "", col = "black", cex = 2, cex.axis = 2.0, lwd = 2.5)

curve(predict(SpeciesAllFall_VEG50, data.frame(Vegetation50=x), type="resp"),add=TRUE, col="black", lwd = 3)
