# Supplementary material for: Factors influencing bird-building collisions in the downtown area of a major North American city
Source: PLoS One. 2019 Nov 6;14(11):e0224164. doi: 10.1371/journal.pone.0224164 (PMC6834121; doi:10.1371/journal.pone.0224164)
Supplement: S3 Appendix — R code for analyses of building-related variables associated with bird collisions (based on 17 buildings with 4 outliers excluded; data in S3 Dataset). (DOCX) [file pone.0224164.s012.docx]

**S3 Appendix**. **R code for analysis with outliers excluded.** R code for analyses of building-related variables associated with bird collisions based on subset of 17 of 21 buildings (excluding potential outliers: stadium, #3, #4, and #17) in downtown Minneapolis, Minnesota, USA. Data used for these analyses are in S3 Dataset.

###Analyses below require the following packages to be installed/loaded:

#lme4

#MASS

#pscl

#AICcmodavg

#QuantPsyc(to generate standardized coefficients)

###Load data

BuildingCompareOUTLIERS_EXCLUDED <- read.table("MN_Building_Collision_Analysis_OUTLIERS_EXCLUDED.txt", header = TRUE) #Loads data to R data frame

attach(BuildingCompareOUTLIERS_EXCLUDED) #Attaches data to R data frame so do not need to reference data object name in remainder of analysis

str(BuildingCompareOUTLIERS_EXCLUDED) #Displays data

###KEY for variable names referenced in this file

#BuildingNumber - Building ID code assigned for study purposes (US Bank Stadium was excluded from this analysis along with three other outlier buildings)

#Quintile - The quintile each building was originally placed into based on total number of collisions observed for Project Birdsafe (2007-2015); this information was used to select 16 study buildings from a larger set of 64 buildings monitored for Project BirdSafe (NA indicates newly selected buildings with no past monitoring history)

#HeightFt - Estimated height of main roof (in feet)

#HeightM - Estimated height of main roof (in meters)

#GlassAreaSqFt - Total estimated area of glass on all building facades (square ft)

#GlassAreaSqM - Total estimated area of glass on all building facades (square m)

#AreaLight - Area of lighted windows across entire building (square m)

#PropLit - Proportion of glass surfaces lit across entire building (calculated by dividing AreaLight by GlassArea)

#Footprint - Horizontal ground area covered by the building (square m); calculated using the building polygon file and ArcGIS's Calculate Geometry option in the shape file's attribute table

#DistanceRiver - Distance (m) from building centroid to nearest edge of Mississippi River corridor (calculated using Euclidian Distance Tool in GIS's Spatial Analyst Tools Directory)

#Vegetation50 - Proportion of vegetated land within 50 m of building edge (includes grass/shrub, deciduous tree cover, and coniferous tree cover; excludes buildings, roads/paved surfaces, and bare soil)

#Vegetation100 - Proportion of vegetated land within 100 m of building edge (includes grass/shrub, deciduous tree cover, and coniferous tree cover; excludes buildings, roads/paved surfaces, and bare soil)

#LowRawFatal2017 - 2017 raw counts of fatal collisions (count is considered low because it excludes birds that possibly collided with skyways connecting buildings, not buildings themselves, and potential predation events, not collisions)

#LowRawFatal2018 - 2018 raw counts of fatal collisions (count is considered low because it excludes birds that possibly collided with skyways connecting buildings, not buildings themselves, and potential predation events, not collisions)

#LowRawAll2017 - 2017 raw counts of all fatal and non-fatal collisions (count is considered low because it excludes birds that possibly collided with skyways connecting buildings, not buildings themselves, and potential predation events, not collisions)

#LowRawAll2018 - 2018 raw counts of all fatal and non-fatal collisions (count is considered low because it excludes birds that possibly collided with skyways connecting buildings, not buildings themselves, and potential predation events, not collisions)

#LowRawNonFatal - Total raw counts of non-fatal collision sacross both 2017 and 2018 (count is considered low because it excludes birds that possibly collided with skyways connecting buildings, not buildings themselves, and potential predation events, not collisions)

#HighRawNonFatal - Total raw counts of non-fatal collisions across both 2017 and 2018 (count is considered high because it includes birds that possibly collided with skyways connecting buildings, not buildings themselves, and potential predation events, not collisions)

#LowRawFatal - Total raw count of fatal collisions across both 2017 and 2018 (count is considered low because it excludes birds that possibly collided with skyways connecting buildings, not buildings themselves, and potential predation events, not collisions)

#HighRawFatal - Total raw count of fatal collisions across both 2017 and 2018 (count is considered high because it includes birds that possibly collided with skyways connecting buildings, not buildings themselves, and potential predation events, not collisions)

#LowAdjustedFatal - Median estimate of bias-adjusted fatalities (i.e., accounting for searcher detection rate and human and animal removal of carcasses) across both 2017 and 2018 based on low raw count of fatal collisions

#HighAdjustedFatal - Median estimate of bias-adjusted fatalities (i.e., accounting for searcher detection rate and human and animal removal of carcasses) across both 2017 and 2018 based on high raw count of fatal collisions

#SpringLowRawFatal - Low raw count of all spring fatal collisions across both 2017 and 2018 (count is considered low because it excludes birds that possibly collided with skyways connecting buildings, not buildings themselves, and potential predation events, not collisions)

#FallLowRawFatal - Low raw count of all fall fatal collisions across both 2017 and 2018 (count is considered low because it excludes birds that possibly collided with skyways connecting buildings, not buildings themselves, and potential predation events, not collisions)

#WTSPLowRawFatal - Low raw count of all White-throated Sparrow fatal collisions across both 2017 and 2018 (count is considered low because it excludes birds that possibly collided with skyways connecting buildings, not buildings themselves, and potential predation events, not collisions)

#NAWALowRawFatal - Low raw count of all Nashville Warbler fatal collisions across both 2017 and 2018 (count is considered low because it excludes birds that possibly collided with skyways connecting buildings, not buildings themselves, and potential predation events, not collisions)

#OVENLowRawFatal - Low raw count of all Ovenbird fatal collisions across both 2017 and 2018 (count is considered low because it excludes birds that possibly collided with skyways connecting buildings, not buildings themselves, and potential predation events, not collisions)

#COYELowRawFatal - Low raw count of all Common Yellowthroat fatal collisions across both 2017 and 2018 (count is considered low because it excludes birds that possibly collided with skyways connecting buildings, not buildings themselves, and potential predation events, not collisions)

#TEWALowRawFatal - Low raw count of all Tennessee Warbler fatal collisions across both 2017 and 2018 (count is considered low because it excludes birds that possibly collided with skyways connecting buildings, not buildings themselves, and potential predation events, not collisions)

#SpeciesFatal - Total number of species observed as fatal collisions across across both 2017 and 2018

#SpeciesNonFatal - Total number of species observed as non-fatal collisions across across both 2017 and 2018

#SpeciesAll - Total number of species observed as fatal and non-fatal collisions across across both 2017 and 2018

#SpeciesAllSpring - Total number of species observed as spring fatal collisions during both 2017 and 2018

#SpeciesAllFall - Total number of species observed as fall fatal collisions during both 2017 and 2018

#ttest to see if collisions differ between 2017 and 2018

t.test(LowRawFatal2017, LowRawFatal2018, alternative = "two.sided", paired = TRUE) # t=-0.29, df = 16, p = 0.78

t.test(LowRawAll2017, LowRawAll2018, alternative = "two.sided", paired = TRUE) # t=-0.05 df = 16, p = 0.96

##NO DIFFERENCE IN FATAL COLLISIONS OR ALL COLLISIONS BETWEEN YEARS, SO FATALITIES FOR BOTH YEARS INCLUDED AS RESPONSE VARIABLE

###Conduct correlation analyses to identify strongly correlated variable pairs for later model selection analyses

AllPredictors <- BuildingCompareOUTLIERS_EXCLUDED[,c(4, 6, 7, 8, 9, 10, 11, 12)] ###Makes data frame with all 8 predictor variables (HeightM, GlassAreaSqM, AreaLight, PropLit, Footprint, DistanceRiver, Vegetation50, Vegetation100)

head(AllPredictors,6) #Print 1st 6 rows to check new data frame

cor(AllPredictors) #Runs 8x8 correlation matrix using all of the predictors

###Only GlassAreaSqM and AreaLight strongly correlated (0.77)

##############################################################################

###(1) Model selection analysis (Low Raw Count of TOTAL Fatal Collisions as Dep. Variable)

##############################################################################

###(A) Determine whether to use Poisson or Negative binomial statistical distribution based on null model and likelihood ratio test(Requires lme4, pscl, and MASS packages);Description of methods for running Likelihood ratio tests and calculating Chi-Square statistics/probabilities is at: http://stats.stackexchange.com/questions/127505/compare-poisson-and-negative-binomial-regression-with-lr-test

LowRawFatal_POISSON <- glm(LowRawFatal ~ 1, family = "poisson") ##Runs Poisson model on the null model (Requires lme4 package)

LowRawFatal_NEGBIN <- glm.nb(LowRawFatal ~ 1) ##Runs Negative Binomial model on the null model (requires MASS package)

###Likelihood ratio test to determine if Negative Binomial fits better than Poisson

2 * (logLik(LowRawFatal_NEGBIN) - logLik(LowRawFatal_POISSON)) ##Returns Chi-square statistic for comparison of two models

pchisq(2 * (logLik(LowRawFatal_NEGBIN) - logLik(LowRawFatal_POISSON)), df = 1, lower.tail = FALSE) ##Returns probability of chi-square statistic for comparison of two models

#Negative binomial supported

B) Conduct model selection exercise using remaining non-correlated predictor variables

###NULL MODEL

LowRawFatal_NULL<- glm.nb(LowRawFatal ~ 1)

###GLOBAL MODEL (Additive terms only)

LowRawFatal_GLOBAL <- glm.nb(LowRawFatal ~ HeightM + GlassAreaSqM + PropLit + Footprint + DistanceRiver + Vegetation50 + Vegetation100) #Excludes AreaLight as described above under part (1)

###RUN STEPWISE BACKWARD AIC FUNCTION (Requires MASS Package)

LowRawFatalSTEP <- stepAIC(LowRawFatal_GLOBAL, scope = list(lower = ~1), Trace = FALSE)

LowRawFatalSTEP

###Results in 3 variables remaining (GlassAreaSqM and Vegetation100)

###Inspect GlassAreaSqM + Vegetation100 Model

LowRawFatal_GLASS_VEG100 <- glm.nb(LowRawFatal ~ GlassAreaSqM + Vegetation100)

LowRawFatal_GLASS_VEG100

summary(LowRawFatal_GLASS_VEG100) ##Generates coefficient estimates along with Standard errors

confint(LowRawFatal_GLASS_VEG100) ##Generates 95% confidence intervals of coefficient estimates

lm.beta(LowRawFatal_GLASS_VEG100) ##Generatess standardized coefficient values (requires QuantPsyc package)

###(C)Plot effects of supported variables

par(mfrow = c(1,1), mai = c(0.5, 0.6, 0.5, 0.6))

LowRawFatal_GLASS <- glm.nb(LowRawFatal ~ GlassAreaSqM)

plot(GlassAreaSqM, LowRawFatal, xlab = "", ylab = "", col = "black", cex = 2, cex.axis = 2.0, lwd = 2.5)

curve(predict(LowRawFatal_GLASS, data.frame(GlassAreaSqM=x), type="resp"),add=TRUE, col="black", lwd = 3)

LowRawFatal_VEG100 <- glm.nb(LowRawFatal ~ Vegetation100)

plot(Vegetation100, LowRawFatal, xlab = "", ylab = "", col = "black", cex = 2, cex.axis = 2.0, lwd = 2.5)

curve(predict(LowRawFatal_VEG100, data.frame(Vegetation100=x), type="resp"),add=TRUE, col="black", lwd = 3)

#################################################################################(2) Model selection analysis (High Median Bias-adjusted Estimates as Dependent Variable)

##############################################################################

###(A) Determine whether to use Poisson or Negative binomial statistical distribution based on null model and likelihood ratio test(Requires lme4, pscl, and MASS packages);Description of methods for running Likelihood ratio tests and calculating Chi-Square statistics/probabilities is at: http://stats.stackexchange.com/questions/127505/compare-poisson-and-negative-binomial-regression-with-lr-test

HighAdjustedFatal_POISSON <- glm(HighAdjustedFatal ~ 1, family = "poisson") ##Runs Poisson model on the null model (Requires lme4 package)

HighAdjustedFatal_NEGBIN <- glm.nb(HighAdjustedFatal ~ 1) ##Runs Negative Binomial model on the null model (requires MASS package)

##Likelihood ratio test to determine if Negative Binomial fits better than Poisson

2 * (logLik(HighAdjustedFatal_NEGBIN) - logLik(HighAdjustedFatal_POISSON)) ##Returns Chi-square statistic for comparison of two models

pchisq(2 * (logLik(HighAdjustedFatal_NEGBIN) - logLik(HighAdjustedFatal_POISSON)), df = 1, lower.tail = FALSE) ##Returns probability of chi-square statistic for comparison of two models

#Negative binomial supported

###(B) Conduct model selection exercise using remaining non-correlated predictor variables

###NULL MODEL

HighAdjustedFatal_NULL <- glm.nb(HighAdjustedFatal ~ 1)

###GLOBAL MODEL (Additive terms only)

HighAdjustedFatal_GLOBAL <- glm.nb(HighAdjustedFatal ~ HeightM + GlassAreaSqM + PropLit + Footprint + DistanceRiver + Vegetation50 + Vegetation100) #Excludes AreaLight as described above under part (1)

HighAdjustedFatal_GLOBAL <- glm.nb(HighAdjustedFatal ~ HeightM + GlassAreaSqM + PropLit + Footprint + DistanceRiver + Vegetation50 + Vegetation100, control = list(trace=TRUE))

###RUN STEPWISE BACKWARD AIC FUNCTION (Requires MASS Package)

HighAdjustedFatalSTEP <- stepAIC(HighAdjustedFatal_GLOBAL, scope = list(lower = ~1), Trace = FALSE)

HighAdjustedFatalSTEP

###RESULTS IN MODEL NOT CONVERGING

##############################################################################

###(3) Model selection analysis (Low Raw Count of SPRING Fatal Collisions as Depend. Var.)

##############################################################################

###(A) Determine whether to use Poisson or Negative binomial statistical distribution based on null model and likelihood ratio test(Requires lme4, pscl, and MASS packages);Description of methods for running Likelihood ratio tests and calculating Chi-Square statistics/probabilities is at: http://stats.stackexchange.com/questions/127505/compare-poisson-and-negative-binomial-regression-with-lr-test

SpringLowRawFatal_POISSON <- glm(SpringLowRawFatal ~ 1, family = "poisson") ##Runs Poisson model on the null model (Requires lme4 package)

SpringLowRawFatal_NEGBIN <- glm.nb(SpringLowRawFatal ~ 1) ##Runs Negative Binomial model on the null model (requires MASS package)

##Likelihood ratio test to determine if Negative Binomial fits better than Poisson

2 * (logLik(SpringLowRawFatal_NEGBIN) - logLik(SpringLowRawFatal_POISSON)) ##Returns Chi-square statistic for comparison of two models

pchisq(2 * (logLik(SpringLowRawFatal_NEGBIN) - logLik(SpringLowRawFatal_POISSON)), df = 1, lower.tail = FALSE) ##Returns probability of chi-square statistic for comparison of two models

#Negative Binomial Supported

###(B) Conduct model selection exercise using remaining non-correlated predictor variables

###NULL MODEL

SpringLowRawFatal_NULL<- glm.nb(SpringLowRawFatal ~ 1)

###GLOBAL MODEL (Additive terms only)

SpringLowRawFatal_GLOBAL <- glm.nb(SpringLowRawFatal ~ HeightM + GlassAreaSqM + PropLit + Footprint + DistanceRiver + Vegetation50 + Vegetation100) #Excludes AreaLight as described above under part (1)

###RUN STEPWISE BACKWARD AIC FUNCTION (Requires MASS Package)

SpringLowRawFatalSTEP <- stepAIC(SpringLowRawFatal_GLOBAL, scope = list(lower = ~1), Trace = FALSE)

SpringLowRawFatalSTEP

###Results in 3 variables remaining (Prop Lit, Vegetation50, and Vegetation100)

###Investigating the PropLit + Vegetation50 + Vegetation100 Model

SpringLowRawFatal_PROPLit_VEG50_VEG100 <- glm.nb(SpringLowRawFatal ~ PropLit + Vegetation50 + Vegetation100)

SpringLowRawFatal_PROPLit_VEG50_VEG100

summary(SpringLowRawFatal_PROPLit_VEG50_VEG100) ##Generates coefficient estimates along with Standard errors

confint(SpringLowRawFatal_PROPLit_VEG50_VEG100) ##Generates 95% confidence intervals of coefficient estimates

lm.beta(SpringLowRawFatal_PROPLit_VEG50_VEG100) ##Generatess standardized coefficient values (requires QuantPsyc package)

###(C)Plot effects of supported variables

par(mfrow = c(1,1), mai = c(0.5, 0.6, 0.5, 0.6))

SpringLowRawFatal_PROPLit <- glm.nb(SpringLowRawFatal ~ PropLit)

plot(PropLit, SpringLowRawFatal, xlab = "", ylab = "", col = "black", cex = 2, cex.axis = 2.0, lwd = 2.5)

curve(predict(SpringLowRawFatal_PROPLit, data.frame(PropLit=x), type="resp"),add=TRUE, col="black", lwd = 3)

SpringLowRawFatal_VEG50 <- glm.nb(SpringLowRawFatal ~ Vegetation50)

plot(Vegetation50, SpringLowRawFatal, xlab = "", ylab = "", col = "black", cex = 2, cex.axis = 2.0, lwd = 2.5)

curve(predict(SpringLowRawFatal_VEG50, data.frame(Vegetation50=x), type="resp"),add=TRUE, col="black", lwd = 3)

SpringLowRawFatal_VEG100 <- glm.nb(SpringLowRawFatal ~ Vegetation100)

plot(Vegetation100, SpringLowRawFatal, xlab = "", ylab = "", col = "black", cex = 2, cex.axis = 2.0, lwd = 2.5)

curve(predict(SpringLowRawFatal_VEG100, data.frame(Vegetation100=x), type="resp"),add=TRUE, col="black", lwd = 3)

##############################################################################

###(4) Model selection analysis (Low Raw Count of FALL Fatal Collisions as Depend. Var.)

##############################################################################

###(A) Determine whether to use Poisson or Negative binomial statistical distribution based on null model and likelihood ratio test(Requires lme4, pscl, and MASS packages);Description of methods for running Likelihood ratio tests and calculating Chi-Square statistics/probabilities is at: http://stats.stackexchange.com/questions/127505/compare-poisson-and-negative-binomial-regression-with-lr-test

FallLowRawFatal_POISSON <- glm(FallLowRawFatal ~ 1, family = "poisson") ##Runs Poisson model on the null model (Requires lme4 package)

FallLowRawFatal_NEGBIN <- glm.nb(FallLowRawFatal ~ 1) ##Runs Negative Binomial model on the null model (requires MASS package)

##Likelihood ratio test to determine if Negative Binomial fits better than Poisson

2 * (logLik(FallLowRawFatal_NEGBIN) - logLik(FallLowRawFatal_POISSON)) ##Returns Chi-square statistic for comparison of two models

pchisq(2 * (logLik(FallLowRawFatal_NEGBIN) - logLik(FallLowRawFatal_POISSON)), df = 1, lower.tail = FALSE) ##Returns probability of chi-square statistic for comparison of two models

##Negative Binomial supported

###(B) Conduct model selection exercise using remaining non-correlated predictor variables

###NULL MODEL

FallLowRawFatal_NULL<- glm.nb(FallLowRawFatal ~ 1)

###GLOBAL MODEL (Additive terms only)

FallLowRawFatal_GLOBAL <- glm.nb(FallLowRawFatal ~ HeightM + GlassAreaSqM + PropLit + Footprint + DistanceRiver + Vegetation50 + Vegetation100) #Excludes AreaLight as described above under part (1)

###RUN STEPWISE BACKWARD AIC FUNCTION (Requires MASS Package)

FallLowRawFatalSTEP <- stepAIC(FallLowRawFatal_GLOBAL, scope = list(lower = ~1), Trace = FALSE)

FallLowRawFatalSTEP

###Results in 3 variables remaining (GlassAreaSqM, PropLit, and Vegetation100)

###Investigating GlassAreaSqM + PropLit + Vegetation 100 Model

FallLowRawFatal_GLASS_PROPLit_VEG100 <- glm.nb(FallLowRawFatal ~ GlassAreaSqM + PropLit + Vegetation100)

FallLowRawFatal_GLASS_PROPLit_VEG100

summary(FallLowRawFatal_GLASS_PROPLit_VEG100) ##Generates coefficient estimates along with Standard errors

confint(FallLowRawFatal_GLASS_PROPLit_VEG100) ##Generates 95% confidence intervals of coefficient estimates

lm.beta(FallLowRawFatal_GLASS_PROPLit_VEG100) ##Generatess standardized coefficient values (requires QuantPsyc package)

###(C)Plot effects of supported variables

par(mfrow = c(1,1), mai = c(0.5, 0.6, 0.5, 0.6))

FallLowRawFatal_GLASS <- glm.nb(FallLowRawFatal ~ GlassAreaSqM)

plot(GlassAreaSqM, FallLowRawFatal, xlab = "", ylab = "", col = "black", cex = 2, cex.axis = 2.0, lwd = 2.5)

curve(predict(FallLowRawFatal_GLASS, data.frame(GlassAreaSqM=x), type="resp"),add=TRUE, col="black", lwd = 3)

FallLowRawFatal_PROPLit <- glm.nb(FallLowRawFatal ~ PropLit)

plot(PropLit, FallLowRawFatal, xlab = "", ylab = "", col = "black", cex = 2, cex.axis = 2.0, lwd = 2.5)

curve(predict(FallLowRawFatal_PROPLit, data.frame(PropLit=x), type="resp"),add=TRUE, col="black", lwd = 3)

FallLowRawFatal_VEG100 <- glm.nb(FallLowRawFatal ~ Vegetation100)

plot(Vegetation100, FallLowRawFatal, xlab = "", ylab = "", col = "black", cex = 2, cex.axis = 2.0, lwd = 2.5)

curve(predict(FallLowRawFatal_VEG100, data.frame(Vegetation100=x), type="resp"),add=TRUE, col="black", lwd = 3)

##############################################################################

###(5)Model selection analysis (Low Raw Count of TOTAL White-throated Sparrow Fatal Collisions as Dep. var)

##############################################################################

###(A) Determine whether to use Poisson or Negative binomial statistical distribution based on null model and likelihood ratio test(Requires lme4, pscl, and MASS packages);Description of methods for running Likelihood ratio tests and calculating Chi-Square statistics/probabilities is at: http://stats.stackexchange.com/questions/127505/compare-poisson-and-negative-binomial-regression-with-lr-test

WTSPLowRawFatal_POISSON <- glm(WTSPLowRawFatal ~ 1, family = "poisson") ##Runs Poisson model on the null model (Requires lme4 package)

WTSPLowRawFatal_NEGBIN <- glm.nb(WTSPLowRawFatal~ 1) ##Runs Negative Binomial model on the null model (requires MASS package)

##Likelihood ratio test to determine if Negative Binomial fits better than Poisson

2 * (logLik(WTSPLowRawFatal_NEGBIN) - logLik(WTSPLowRawFatal_POISSON)) ##Returns Chi-square statistic for comparison of two models

pchisq(2 * (logLik(WTSPLowRawFatal_NEGBIN) - logLik(WTSPLowRawFatal_POISSON)), df = 1, lower.tail = FALSE) ##Returns probability of chi-square statistic for comparison of two models

#Negative binomial supported

###(B) Conduct model selection exercise using remaining non-correlated predictor variables

###NULL MODEL

WTSPLowRawFatal_NULL<- glm.nb(WTSPLowRawFatal ~ 1)

###GLOBAL MODEL (Additive terms only)

WTSPLowRawFatal_GLOBAL <- glm.nb(WTSPLowRawFatal ~ HeightM + GlassAreaSqM + PropLit + Footprint + DistanceRiver + Vegetation50 + Vegetation100) #Excludes AreaLight as described above under part (1)

###RUN STEPWISE BACKWARD AIC FUNCTION (Requires MASS Package)

WTSPLowRawFatalSTEP <- stepAIC(WTSPLowRawFatal_GLOBAL, scope = list(lower = ~1), Trace = FALSE)

WTSPLowRawFatalSTEP

###Results in 1 variable remaining (GlassAreaSqM)

###Inspect GlassAreaSqM Model

WTSPLowRawFatal_GLASS <- glm.nb(WTSPLowRawFatal ~ GlassAreaSqM)

WTSPLowRawFatal_GLASS

summary(WTSPLowRawFatal_GLASS) ##Generates coefficient estimates along with Standard errors

confint(WTSPLowRawFatal_GLASS) ##Generates 95% confidence intervals of coefficient estimates

lm.beta(WTSPLowRawFatal_GLASS) ##Generatess standardized coefficient values (requires QuantPsyc package)

###(C)Plot effects of supported variables

par(mfrow = c(1,1), mai = c(0.5, 0.6, 0.5, 0.6))

WTSPLowRawFatal_GLASS <- glm.nb(WTSPLowRawFatal ~ GlassAreaSqM)

plot(GlassAreaSqM, WTSPLowRawFatal, xlab = "", ylab = "", col = "black", cex = 2, cex.axis = 2.0, lwd = 2.5)

curve(predict(WTSPLowRawFatal_GLASS, data.frame(GlassAreaSqM=x), type="resp"),add=TRUE, col="black", lwd = 3)

##############################################################################

###(6)Model selection analysis (Low Raw Count of TOTAL Nashville Warbler Fatal Collisions as Dep var

##############################################################################

###(A) Determine whether to use Poisson or Negative binomial statistical distribution based on null model and likelihood ratio test(Requires lme4, pscl, and MASS packages);Description of methods for running Likelihood ratio tests and calculating Chi-Square statistics/probabilities is at: http://stats.stackexchange.com/questions/127505/compare-poisson-and-negative-binomial-regression-with-lr-test

NAWALowRawFatal_POISSON <- glm(NAWALowRawFatal ~ 1, family = "poisson") ##Runs Poisson model on the null model (Requires lme4 package)

NAWALowRawFatal_NEGBIN <- glm.nb(NAWALowRawFatal ~ 1) ##Runs Negative Binomial model on the null model (requires MASS package)

##Likelihood ratio test to determine if Negative Binomial fits better than Poisson

2 * (logLik(NAWALowRawFatal_NEGBIN) - logLik(NAWALowRawFatal_POISSON)) ##Returns Chi-square statistic for comparison of two models

pchisq(2 * (logLik(NAWALowRawFatal_NEGBIN) - logLik(NAWALowRawFatal_POISSON)), df = 1, lower.tail = FALSE) ##Returns probability of chi-square statistic for comparison of two models

#Negative binomial supported

###(B) Conduct model selection exercise using remaining non-correlated predictor variables#####NULL MODEL

NAWALowRawFatal_NULL<- glm.nb(NAWALowRawFatal ~ 1)

###GLOBAL MODEL (Additive terms only)

NAWALowRawFatal_GLOBAL <- glm.nb(NAWALowRawFatal ~ HeightM + GlassAreaSqM + PropLit + Footprint + DistanceRiver + Vegetation50 + Vegetation100) #Excludes AreaLight as described above under part (1)

###RUN STEPWISE BACKWARD AIC FUNCTION (Requires MASS Package)

NAWALowRawFatalSTEP <- stepAIC(NAWALowRawFatal_GLOBAL, scope = list(lower = ~1), Trace = FALSE)

NAWALowRawFatalSTEP

###Results in 3 variables remaining (Footprint, HeightM, and Vegetation50)

###Inspect Footprint + HeightM + Vegetation50 model

NAWALowRawFatal_FOOT_HEIGHT_VEG50 <- glm.nb(NAWALowRawFatal ~ Footprint + HeightM + Vegetation50)

NAWALowRawFatal_FOOT_HEIGHT_VEG50

summary(NAWALowRawFatal_FOOT_HEIGHT_VEG50) ##Generates coefficient estimates along with Standard errors

confint(NAWALowRawFatal_FOOT_HEIGHT_VEG50) ##Generates 95% confidence intervals of coefficient estimates

lm.beta(NAWALowRawFatal_FOOT_HEIGHT_VEG50) ##Generatess standardized coefficient values (requires QuantPsyc package)

###(C)Plot effects of supported variables

par(mfrow = c(1,1), mai = c(0.5, 0.6, 0.5, 0.6))

NAWALowRawFatal_FOOTPRINT <- glm.nb(NAWALowRawFatal ~ Footprint)

plot(Footprint, NAWALowRawFatal, xlab = "", ylab = "", col = "black", cex = 2, cex.axis = 2.0, lwd = 2.5)

curve(predict(NAWALowRawFatal_FOOTPRINT, data.frame(Footprint=x), type="resp"),add=TRUE, col="black", lwd = 3)

NAWALowRawFatal_HEIGHT <- glm.nb(NAWALowRawFatal ~ HeightM)

plot(HeightM, NAWALowRawFatal, xlab = "", ylab = "", col = "black", cex = 2, cex.axis = 2.0, lwd = 2.5)

curve(predict(NAWALowRawFatal_HEIGHT, data.frame(HeightM=x), type="resp"),add=TRUE, col="black", lwd = 3)

NAWALowRawFatal_VEG50 <- glm.nb(NAWALowRawFatal ~ Vegetation50)

plot(Vegetation50, NAWALowRawFatal, xlab = "", ylab = "", col = "black", cex = 2, cex.axis = 2.0, lwd = 2.5)

curve(predict(NAWALowRawFatal_VEG50, data.frame(Vegetation50=x), type="resp"),add=TRUE, col="black", lwd = 3)

##############################################################################

###(7)Model selection analysis (Low Raw Count of TOTAL Ovenbird Fatal Collisions as Dep. var.

##############################################################################

###(A) Determine whether to use Poisson or Negative binomial statistical distribution based on null model and likelihood ratio test(Requires lme4, pscl, and MASS packages);Description of methods for running Likelihood ratio tests and calculating Chi-Square statistics/probabilities is at: http://stats.stackexchange.com/questions/127505/compare-poisson-and-negative-binomial-regression-with-lr-test

OVENLowRawFatal_POISSON <- glm(OVENLowRawFatal ~ 1, family = "poisson") ##Runs Poisson model on the null model (Requires lme4 package)

OVENLowRawFatal_NEGBIN <- glm.nb(OVENLowRawFatal ~ 1) ##Runs Negative Binomial model on the null model (requires MASS package)

##Likelihood ratio test to determine if Negative Binomial fits better than Poisson

2 * (logLik(OVENLowRawFatal_NEGBIN) - logLik(OVENLowRawFatal_POISSON)) ##Returns Chi-square statistic for comparison of two models

pchisq(2 * (logLik(OVENLowRawFatal_NEGBIN) - logLik(OVENLowRawFatal_POISSON)), df = 1, lower.tail = FALSE) ##Returns probability of chi-square statistic for comparison of two models

#Negative binomial supported

###(B) Conduct model selection exercise using remaining non-correlated predictor variables

###NULL MODEL

OVENLowRawFatal_NULL<- glm.nb(OVENLowRawFatal ~ 1)

###GLOBAL MODEL (Additive terms only)

OVENLowRawFatal_GLOBAL <- glm.nb(OVENLowRawFatal ~ HeightM + GlassAreaSqM + PropLit + Footprint + DistanceRiver + Vegetation50 + Vegetation100) #Excludes AreaLight as described above under part (1)

###RUN STEPWISE BACKWARD AIC FUNCTION (Requires MASS Package)

OVENLowRawFatalSTEP <- stepAIC(OVENLowRawFatal_GLOBAL, scope = list(lower = ~1), Trace = FALSE, k=2)

OVENLowRawFatalSTEP

###Results in 1 variable remaining (GlassAreaSqM)

###Inspect GlassAreaSqM Model

OVENLowRawFatal_GLASS <- glm.nb(OVENLowRawFatal ~ GlassAreaSqM)

OVENLowRawFatal_GLASS

summary(OVENLowRawFatal_GLASS) ##Generates coefficient estimates along with Standard errors

confint(OVENLowRawFatal_GLASS) ##Generates 95% confidence intervals of coefficient estimates

lm.beta(OVENLowRawFatal_GLASS) ##Generatess standardized coefficient values (requires QuantPsyc package)

###(C)Plot effects of supported variables

par(mfrow = c(1,1), mai = c(0.5, 0.6, 0.5, 0.6))

OVENLowRawFatal_GLASS <- glm.nb(OVENLowRawFatal ~ GlassAreaSqM)

plot(GlassAreaSqM, OVENLowRawFatal, xlab = "", ylab = "", col = "black", cex = 2, cex.axis = 2.0, lwd = 2.5)

curve(predict(OVENLowRawFatal_GLASS, data.frame(GlassAreaSqM=x), type="resp"),add=TRUE, col="black", lwd = 3)

#############################################################################

###(8)Model selection analysis (Low Raw Count of TOTAL Common Yellowthroat Fatal Collisions as Dep. var.

##############################################################################

###(A) Determine whether to use Poisson or Negative binomial statistical distribution based on null model and likelihood ratio test(Requires lme4, pscl, and MASS packages);Description of methods for running Likelihood ratio tests and calculating Chi-Square statistics/probabilities is at: http://stats.stackexchange.com/questions/127505/compare-poisson-and-negative-binomial-regression-with-lr-test

COYELowRawFatal_POISSON <- glm(COYELowRawFatal ~ 1, family = "poisson") ##Runs Poisson model on the null model (Requires lme4 package)

COYELowRawFatal_NEGBIN <- glm.nb(COYELowRawFatal ~ 1) ##Runs Negative Binomial model on the null model (requires MASS package)

##Likelihood ratio test to determine if Negative Binomial fits better than Poisson

2 * (logLik(COYELowRawFatal_NEGBIN) - logLik(COYELowRawFatal_POISSON)) ##Returns Chi-square statistic for comparison of two models

pchisq(2 * (logLik(COYELowRawFatal_NEGBIN) - logLik(COYELowRawFatal_POISSON)), df = 1, lower.tail = FALSE) ##Returns probability of chi-square statistic for comparison of two models

#Negative binomial supported

###(B) Conduct model selection exercise using remaining non-correlated predictor variables

###NULL MODEL

COYELowRawFatal_NULL<- glm.nb(COYELowRawFatal ~ 1)

###GLOBAL MODEL (Additive terms only)

COYELowRawFatal_GLOBAL <- glm.nb(COYELowRawFatal ~ HeightM + GlassAreaSqM + PropLit + Footprint + DistanceRiver + Vegetation50 + Vegetation100) #Excludes AreaLight as described above under part (1)

###RUN STEPWISE BACKWARD AIC FUNCTION (Requires MASS Package)

COYELowRawFatalSTEP <- stepAIC(COYELowRawFatal_GLOBAL, scope = list(lower = ~1), Trace = FALSE)

COYELowRawFatalSTEP

###Results in 5 variables remaining (GlassAreaSqM, PropLit, Footprint, Vegetation50, and Vegetation100)

###Insepct GlassAreaSqM + PropLit + Footprint + Vegetation50 + Vegetation100 Model

COYELowRawFatal_GLASS_PROPLit_FOOTPRINT_VEG50_VEG100 <- glm.nb(COYELowRawFatal ~ GlassAreaSqM + PropLit + Footprint + Vegetation50 + Vegetation100)

COYELowRawFatal_GLASS_PROPLit_FOOTPRINT_VEG50_VEG100

summary(COYELowRawFatal_GLASS_PROPLit_FOOTPRINT_VEG50_VEG100) ##Generates coefficient estimates along with Standard errors

confint(COYELowRawFatal_GLASS_PROPLit_FOOTPRINT_VEG50_VEG100) ##Generates 95% confidence intervals of coefficient estimates

lm.beta(COYELowRawFatal_GLASS_PROPLit_FOOTPRINT_VEG50_VEG100) ##Generatess standardized coefficient values (requires QuantPsyc package)

###(C)Plot effects of supported variables

par(mfrow = c(1,1), mai = c(0.5, 0.6, 0.5, 0.6))

COYELowRawFatal_GLASS <- glm.nb(COYELowRawFatal ~ GlassAreaSqM)

plot(GlassAreaSqM, COYELowRawFatal, xlab = "", ylab = "", col = "black", cex = 2, cex.axis = 2.0, lwd = 2.5)

curve(predict(COYELowRawFatal_GLASS, data.frame(GlassAreaSqM=x), type="resp"),add=TRUE, col="black", lwd = 3)

COYELowRawFatal_PROPLit <- glm.nb(COYELowRawFatal ~ PropLit)

plot(PropLit, COYELowRawFatal, xlab = "", ylab = "", col = "black", cex = 2, cex.axis = 2.0, lwd = 2.5)

curve(predict(COYELowRawFatal_PROPLit, data.frame(PropLit=x), type="resp"),add=TRUE, col="black", lwd = 3)

COYELowRawFatal_FOOTPRINT <- glm.nb(COYELowRawFatal ~ Footprint)

plot(Footprint, COYELowRawFatal, xlab = "", ylab = "", col = "black", cex = 2, cex.axis = 2.0, lwd = 2.5)

curve(predict(COYELowRawFatal_FOOTPRINT, data.frame(Footprint=x), type="resp"),add=TRUE, col="black", lwd = 3)

COYELowRawFatal_VEG50 <- glm.nb(COYELowRawFatal ~ Vegetation50)

plot(Vegetation50, COYELowRawFatal, xlab = "", ylab = "", col = "black", cex = 2, cex.axis = 2.0, lwd = 2.5)

curve(predict(COYELowRawFatal_VEG50, data.frame(Vegetation50=x), type="resp"),add=TRUE, col="black", lwd = 3)

COYELowRawFatal_VEG100 <- glm.nb(COYELowRawFatal ~ Vegetation50)

plot(Vegetation50, COYELowRawFatal, xlab = "", ylab = "", col = "black", cex = 2, cex.axis = 2.0, lwd = 2.5)

curve(predict(COYELowRawFatal_VEG100, data.frame(Vegetation50=x), type="resp"),add=TRUE, col="black", lwd = 3)

##############################################################################

###(9)Model selection analysis (Low Raw Count of TOTAL Tennessee Warbler Fatal Collisions as Dep. var

##############################################################################

###(A) Determine whether to use Poisson or Negative binomial statistical distribution based on null model and likelihood ratio test(Requires lme4, pscl, and MASS packages);Description of methods for running Likelihood ratio tests and calculating Chi-Square statistics/probabilities is at: http://stats.stackexchange.com/questions/127505/compare-poisson-and-negative-binomial-regression-with-lr-test

TEWALowRawFatal_POISSON <- glm(TEWALowRawFatal ~ 1, family = "poisson") ##Runs Poisson model on the null model (Requires lme4 package)

TEWADeadLowRaw_NEGBIN <- glm.nb(TEWALowRawFatal ~ 1) ##Runs Negative Binomial model on the null model (requires MASS package)

##Likelihood ratio test to determine if Negative Binomial fits better than Poisson

2 * (logLik(TEWADeadLowRaw_NEGBIN) - logLik(TEWALowRawFatal_POISSON)) ##Returns Chi-square statistic for comparison of two models

pchisq(2 * (logLik(TEWADeadLowRaw_NEGBIN) - logLik(TEWALowRawFatal_POISSON)), df = 1, lower.tail = FALSE) ##Returns probability of chi-square statistic for comparison of two models

#Negative binomial supported

###(B) Conduct model selection exercise using remaining non-correlated predictor variables

###NULL MODEL

TEWALowRawFatal_NULL<- glm.nb(TEWALowRawFatal ~ 1)

###GLOBAL MODEL (Additive terms only)

TEWALowRawFatal_GLOBAL <- glm.nb(TEWALowRawFatal ~ HeightM + GlassAreaSqM + PropLit + Footprint + DistanceRiver + Vegetation50 + Vegetation100) #Excludes AreaLight as described above under part (1)

###RUN STEPWISE BACKWARD AIC FUNCTION (Requires MASS Package)

TEWALowRawFatalSTEP <- stepAIC(TEWALowRawFatal_GLOBAL, scope = list(lower = ~1), Trace = FALSE)

TEWALowRawFatalTEP

###Results in NULL MODEL BEING MOST SUPPORTED

##############################################################################

###(10 Model selection analysis (Number of Speices Colliding across entire study as Dep. var.)

##############################################################################

###(A) Determine whether to use Poisson or Negative binomial statistical distribution based on null model and likelihood ratio test(Requires lme4, pscl, and MASS packages);Description of methods for running Likelihood ratio tests and calculating Chi-Square statistics/probabilities is at: http://stats.stackexchange.com/questions/127505/compare-poisson-and-negative-binomial-regression-with-lr-test

SpeciesAll_POISSON <- glm(SpeciesAll ~ 1, family = "poisson") ##Runs Poisson model on the null model (Requires lme4 package)

SpeciesAll_NEGBIN <- glm.nb(SpeciesAll ~ 1) ##Runs Negative Binomial model on the null model (requires MASS package)

##Likelihood ratio test to determine if Negative Binomial fits better than Poisson

2 * (logLik(SpeciesAll_NEGBIN) - logLik(SpeciesAll_POISSON)) ##Returns Chi-square statistic for comparison of two models

pchisq(2 * (logLik(SpeciesAll_NEGBIN) - logLik(SpeciesAll_POISSON)), df = 1, lower.tail = FALSE) ##Returns probability of chi-square statistic for comparison of two models

#Negative binomial supported

###(B) Conduct model selection exercise using remaining non-correlated predictor variables

###NULL MODEL

SpeciesAll_NULL<- glm.nb(SpeciesAll ~ 1)

###GLOBAL MODEL (Additive terms only)

SpeciesAll_GLOBAL <- glm.nb(SpeciesAll ~ HeightM + GlassAreaSqM + PropLit + Footprint + DistanceRiver + Vegetation50 + Vegetation100) #Excludes AreaLight as described above under part (1)

###RUN STEPWISE BACKWARD AIC FUNCTION (Requires MASS Package)

SpeciesAllSTEP <- stepAIC(SpeciesAll_GLOBAL, scope = list(lower = ~1), Trace = FALSE)

SpeciesAllSTEP

###Results in 3 variables remaining (GlassAreaSqM, PropLit, and Vegetation100)

###Insepect GlasswAreaSqM + PropLit + Vegetation100 model

SpeciesAll_GLASS_PROPLit_VEG100 <- glm.nb(SpeciesAll ~ GlassAreaSqM + PropLit + Vegetation100)

SpeciesAll_GLASS_PROPLit_VEG100

summary(SpeciesAll_GLASS_PROPLit_VEG100) ##Generates coefficient estimates along with Standard errors

confint(SpeciesAll_GLASS_PROPLit_VEG100) ##Generates 95% confidence intervals of coefficient estimates

lm.beta(SpeciesAll_GLASS_PROPLit_VEG100) ##Generatess standardized coefficient values (requires QuantPsyc package)

###95% CI for Roof Height overlaps zero, so only include Footprint and Vegetation50

###(C)Plot effects of supported variables

par(mfrow = c(1,1), mai = c(0.5, 0.6, 0.5, 0.6))

SpeciesAll_GLASS <- glm.nb(SpeciesAll ~ GlassAreaSqM)

plot(GlassAreaSqM, SpeciesAll, xlab = "", ylab = "", col = "black", cex = 2, cex.axis = 2.0, lwd = 2.5)

curve(predict(SpeciesAll_GLASS, data.frame(GlassAreaSqM=x), type="resp"),add=TRUE, col="black", lwd = 3)

SpeciesAll_PROPLit <- glm.nb(SpeciesAll ~ PropLit)

plot(PropLit, SpeciesAll, xlab = "", ylab = "", col = "black", cex = 2, cex.axis = 2.0, lwd = 2.5)

curve(predict(SpeciesAll_PROPLit, data.frame( PropLit=x), type="resp"),add=TRUE, col="black", lwd = 3)

SpeciesAll_VEG100 <- glm.nb(SpeciesAll ~ Vegetation100)

plot(Vegetation100, SpeciesAll, xlab = "", ylab = "", col = "black", cex = 2, cex.axis = 2.0, lwd = 2.5)

curve(predict(SpeciesAll_VEG100, data.frame(Vegetation100=x), type="resp"),add=TRUE, col="black", lwd = 3)

##############################################################################

###(11)Model selection analysis (Number of Speices Colliding in spring as Dependent variable

##############################################################################

###(A) Determine whether to use Poisson or Negative binomial statistical distribution based on null model and likelihood ratio test(Requires lme4, pscl, and MASS packages);Description of methods for running Likelihood ratio tests and calculating Chi-Square statistics/probabilities is at: http://stats.stackexchange.com/questions/127505/compare-poisson-and-negative-binomial-regression-with-lr-test

SpeciesAllSpring_POISSON <- glm(SpeciesAllSpring ~ 1, family = "poisson") ##Runs Poisson model on the null model (Requires lme4 package)

SpeciesAllSpring_NEGBIN <- glm.nb(SpeciesAllSpring ~ 1) ##Runs Negative Binomial model on the null model (requires MASS package)

##Likelihood ratio test to determine if Negative Binomial fits better than Poisson

2 * (logLik(SpeciesAllSpring_NEGBIN) - logLik(SpeciesAllSpring_POISSON)) ##Returns Chi-square statistic for comparison of two models

pchisq(2 * (logLik(SpeciesAllSpring_NEGBIN) - logLik(SpeciesAllSpring_POISSON)), df = 1, lower.tail = FALSE) ##Returns probability of chi-square statistic for comparison of two models

#Negative binomial supported

###(B) Conduct model selection exercise using remaining non-correlated predictor variables

###NULL MODEL

SpeciesAllSpring_NULL <- glm.nb(SpeciesAllSpring ~ 1)

###GLOBAL MODEL (Additive terms only)

SpeciesAllSpring_GLOBAL <- glm.nb(SpeciesAllSpring ~ HeightM + GlassAreaSqM + PropLit + Footprint + DistanceRiver + Vegetation50 + Vegetation100) #Excludes AreaLight as described above under part (1)

###RUN STEPWISE BACKWARD AIC FUNCTION (Requires MASS Package)

SpeciesAllSpringSTEP <- stepAIC(SpeciesAllSpring_GLOBAL, scope = list(lower = ~1), Trace = FALSE)

SpeciesAllSpringSTEP

###Results in 2 variables remaining (PropLit + Vegetation100)

###Inspect PropLit + Vegetation100 Model

SpeciesAllSpring_PROPLit_VEG100 <- glm.nb(SpeciesAllSpring ~ PropLit + Vegetation100)

SpeciesAllSpring_PROPLit_VEG100

summary(SpeciesAllSpring_PROPLit_VEG100) ##Generates coefficient estimates along with Standard errors

confint(SpeciesAllSpring_PROPLit_VEG100) ##Generates 95% confidence intervals of coefficient estimates

lm.beta(SpeciesAllSpring_PROPLit_VEG100) ##Generatess standardized coefficient values (requires QuantPsyc package)

###(C) Plot effects of supported variables

par(mfrow = c(1,1), mai = c(0.5, 0.6, 0.5, 0.6))

SpeciesAllSpring_PROPLit <- glm.nb(SpeciesAllSpring ~ PropLit)

plot(PropLit, SpeciesAllSpring, xlab = "", ylab = "", col = "black", cex = 2, cex.axis = 2.0, lwd = 2.5)

curve(predict(SpeciesAllSpring_PROPLit, data.frame(PropLit=x), type="resp"),add=TRUE, col="black", lwd = 3)

SpeciesAllSpring_VEG100 <- glm.nb(SpeciesAllSpring ~ Vegetation100)

plot(Vegetation100, SpeciesAllSpring, xlab = "", ylab = "", col = "black", cex = 2, cex.axis = 2.0, lwd = 2.5)

curve(predict(SpeciesAllSpring_VEG100, data.frame(Vegetation100=x), type="resp"),add=TRUE, col="black", lwd = 3)

##############################################################################

###(12)Model selection analysis (Number of Speices Colliding in fall as Dependent variable

##############################################################################

###(A) Determine whether to use Poisson or Negative binomial statistical distribution based on null model and likelihood ratio test(Requires lme4, pscl, and MASS packages);Description of methods for running Likelihood ratio tests and calculating Chi-Square statistics/probabilities is at: http://stats.stackexchange.com/questions/127505/compare-poisson-and-negative-binomial-regression-with-lr-test

SpeciesAllFall_POISSON <- glm(SpeciesAllFall ~ 1, family = "poisson") ##Runs Poisson model on the null model (Requires lme4 package)

SpeciesAllFall_NEGBIN <- glm.nb(SpeciesAllFall ~ 1) ##Runs Negative Binomial model on the null model (requires MASS package)

##Likelihood ratio test to determine if Negative Binomial fits better than Poisson

2 * (logLik(SpeciesAllFall_NEGBIN) - logLik(SpeciesAllFall_POISSON)) ##Returns Chi-square statistic for comparison of two models

pchisq(2 * (logLik(SpeciesAllFall_NEGBIN) - logLik(SpeciesAllFall_POISSON)), df = 1, lower.tail = FALSE) ##Returns probability of chi-square statistic for comparison of two models

#Negative binomial supported

###(B) Conduct model selection exercise using remaining non-correlated predictor variables

###NULL MODEL

SpeciesAllFall_NULL<- glm.nb(SpeciesAllFall ~ 1)

###GLOBAL MODEL (Additive terms only)

SpeciesAllFall_GLOBAL <- glm.nb(LowRawFatal ~ HeightM + GlassAreaSqM + PropLit + Footprint + DistanceRiver + Vegetation50 + Vegetation100) #Excludes AreaLight as described above under part (1)

###RUN STEPWISE BACKWARD AIC FUNCTION (Requires MASS Package)

SpeciesAllFallSTEP <- stepAIC(SpeciesAllFall_GLOBAL, scope = list(lower = ~1), Trace = FALSE)

SpeciesAllFallSTEP

###Results in 3 variables remaining (GlassAreaSqM, PropLit, and Vegetation100)

###Inspect GlassAreaSqM + PropLit + Vegetation100 Model

SpeciesAllFall_GLASS_PROPLit_VEG100 <- glm.nb(SpeciesAllFall ~ GlassAreaSqM + PropLit + Vegetation100)

SpeciesAllFall_GLASS_PROPLit_VEG100

summary(SpeciesAllFall_GLASS_PROPLit_VEG100) ##Generates coefficient estimates along with Standard errors

confint(SpeciesAllFall_GLASS_PROPLit_VEG100) ##Generates 95% confidence intervals of coefficient estimates

lm.beta(SpeciesAllFall_GLASS_PROPLit_VEG100) ##Generatess standardized coefficient values (requires QuantPsyc package)

###95% CI for Vegetation50 overlaps zero, so only include GlassAreaSqM and Footprint

###(C)Plot effects of supported variables

par(mfrow = c(1,1), mai = c(0.5, 0.6, 0.5, 0.6))

SpeciesAllFall_GLASS <- glm.nb(SpeciesAllFall ~ GlassAreaSqM)

plot(GlassAreaSqM, SpeciesAllFall, xlab = "", ylab = "", col = "black", cex = 2, cex.axis = 2.0, lwd = 2.5)

curve(predict(SpeciesAllFall_GLASS, data.frame(GlassAreaSqM=x), type="resp"),add=TRUE, col="black", lwd = 3)

SpeciesAllFall_PROPLit <- glm.nb(SpeciesAllFall ~ PropLit)

plot(PropLit, SpeciesAllFall, xlab = "", ylab = "", col = "black", cex = 2, cex.axis = 2.0, lwd = 2.5)

curve(predict(SpeciesAllFall_PROPLit, data.frame(PropLit=x), type="resp"),add=TRUE, col="black", lwd = 3)

SpeciesAllFall_VEG100 <- glm.nb(SpeciesAllFall ~ Vegetation100)

plot(Vegetation100, SpeciesAllFall, xlab = "", ylab = "", col = "black", cex = 2, cex.axis = 2.0, lwd = 2.5)

curve(predict(SpeciesAllFall_VEG100, data.frame(Vegetation100=x), type="resp"),add=TRUE, col="black", lwd = 3)
